# Trends in the contribution of greenhouse gas emissions from food and beverage purchases in Mexico: 1989–2020

**DOI:** 10.1186/s12937-024-00955-z

**Published:** 2024-05-18

**Authors:** Francisco Canto-Osorio, Brent A. Langellier, Mishel Unar-Munguia, Tonatiuh Barrientos-Gutiérrez, Juan A. Rivera, Ana V. Diez-Roux, Dalia Stern, Nancy López-Olmedo

**Affiliations:** 1 Center for Research in Population Health, National Institute of Public Health, Av. Universidad 655, Santa María Ahuacatitlán, Cuernavaca, Morelos 62100 Mexico; 2 Dornsife School of Public Health, Drexel University, Philadelphia, PA USA; 3 Center for Research on Health and Nutrition, National Institute of Public Health, Cuernavaca, Morelos Mexico; 4 CONAHCyT–Center for Research on Population Health, National Institute of Public Health, Cuernavaca, Morelos Mexico

**Keywords:** Greenhouse gas emissions, Diet, Trends, Socioeconomic status, Mexico

## Abstract

**Background:**

Assessing the trends in dietary GHGE considering the social patterning is critical for understanding the role that food systems have played and will play in global emissions in countries of the global south. Our aim is to describe dietary greenhouse gas emissions (GHGE) trends (overall and by food group) using data from household food purchase surveys from 1989 to 2020 in Mexico, overall and by education levels and urbanicity.

**Methods:**

We used cross-sectional data from 16 rounds of Mexico’s National Income and Expenditure Survey, a nationally representative survey. The sample size ranged from 11,051 in 1989 to 88,398 in 2020. We estimated the mean total GHGE per adult-equivalent per day (kg CO2-eq/ad-eq/d) for every survey year. Then, we estimated the relative GHGE contribution by food group for each household. These same analyses were conducted stratifying by education and urbanicity.

**Results:**

The mean total GHGE increased from 3.70 (95%CI: 3.57, 3.82) to 4.90 (95% CI 4.62, 5.18) kg CO2-eq/ad-eq/d between 1989 and 2014 and stayed stable between 4.63 (95% CI: 4.53, 4.72) and 4.89 (95% CI: 4.81, 4.96) kg CO2-eq/ad-eq/d from 2016 onwards. In 1989, beef (19.89%, 95% CI: 19.18, 20.59), dairy (16.87%, 95% CI: 16.30, 17.42)), corn (9.61%, 95% CI: 9.00, 10.22), legumes (7.03%, 95% CI: 6.59, 7.46), and beverages (6.99%, 95% CI: 6.66, 7.32) had the highest relative contribution to food GHGE; by 2020, beef was the top contributor (17.68%, 95%CI: 17.46, 17.89) followed by fast food (14.17%, 95% CI: 13.90, 14.43), dairy (11.21%, 95%CI: 11.06, 11.36), beverages (10.09%, 95%CI: 9.94, 10.23), and chicken (10.04%, 95%CI: 9.90, 10.17). Households with higher education levels and those in more urbanized areas contributed more to dietary GHGE across the full period. However, households with lower education levels and those in rural areas had the highest increase in these emissions from 1989 to 2020.

**Conclusions:**

Our results provide insights into the food groups in which the 2023 Mexican Dietary Guidelines may require to focus on improving human and planetary health.

**Supplementary Information:**

The online version contains supplementary material available at 10.1186/s12937-024-00955-z.

## Introduction

Greenhouse gas emissions (GHGE) are a critical driver of global climate change. An estimated one-third of all GHGE comes from the food systems, including agricultural production, storage and distribution, processing and packaging, consumption, waste, and food disposal [1, 2]. Understanding the links between food-related GHGE and how these have changed over time is critical for developing a global strategy to address climate change.

In many countries like Mexico, populations have shifted from traditional to more Westernized diets, and these changes have affected subgroups of the population at different rates [3]. The relative contribution of fruits and vegetables to total daily energy purchases increased from 1984 to 2016 (25.0% for fruits, 75% for vegetables) in Mexican households, but the relative contribution of ultra-processed food increased even more in the same period (120%) [4]. Although the relative contribution of unprocessed red meat, one of the main contributors to GHGE, decreased by 26.5% from 1984 to 2016, the corresponding contribution of processed and ultra-processed meat increased by 83.3% and 225%, respectively [4]. However, we do not know how changes in food purchases affect dietary GHGE for the overall population and by socioeconomic strata (SES).

Research has been limited to describing overall GHGE and the social patterning of dietary GHGE using cross-sectional data. In Mexico, animal products were the main contributors to GHGE across SES groups in 2018. Yet, the relative contribution was higher among the highest SES groups, compared to the lower SES group. In contrast, the relative contribution of plant-based foods was higher among lower SES groups, compared to high SES groups [5]. Assessing the trends in dietary GHGE considering the social patterning is critical for understanding the role that food systems have played and will play in global emissions. This information will be useful to inform future food policies aimed at improving human and planetary health.

Mexico is an important country to study because it ranks 10th worldwide in terms of GHGE, and agriculture is the third most important contributor of GHGE in the country [6]. The objective of our study is to describe dietary GHGE trends (overall and by food group) through household food purchase surveys from 1989 to 2020 in Mexico, overall and by education levels and city size.

## Methods

### Data sources

#### National Income and Expenditure Survey (ENIGH)

We used data from 16 rounds of ENIGH, including 1989 and every other year from 1992 to 2020. The ENIGH is a cross-sectional study of Mexican households conducted by the National Institute of Statistics and Geography of Mexico. Each ENIGH is based on a probability sample using a two-stage stratified clustered sampling design, representative at the national level and among both urban and rural areas. All ENIGHs were collected between August and November, except for ENIGH 1994, which was collected between September and December. Detailed information about data collection is available elsewhere [7]. 

For every survey cycle, each household’s daily food and beverage purchasing was collected for seven consecutive days using a food diary. Food and beverage purchasing was reported by the household member responsible for the purchases. The food diary includes the name of food or beverage items, the quantity purchased (liters or kilograms), and the expenditure (MXN). Over time, ENIGH has collected different numbers of items: the 1989–1992 surveys include 170 items; 1994–2000: 177 items; 2002–2004: 230 items; and 2006–2020: 234 items. The increase in items is characterized by a disaggregation of foods (i.e., in the first survey, the item was general pork while in the most recent surveys, the items were meat from parts of the pork) (Supplementary Table 1).

From rounds 1989 to 2020, the smallest sample size was 8,899 households in 2012 and the largest was 88,398 households in 2020. We excluded households that did not report purchases of food or beverages, and households that only reported purchases in restaurants, cafes, bars, and low-budget restaurants (*n* = 5,962, 1.32% across surveys over time; with the lowest 0.66% in 2018 and the highest 3.42% in 1998), given that purchases at those establishments only include the amount of money spent, but not what was purchased. The final analytical sample for each survey is presented in Supplemental Table 2.

### Household-level GHGEs

We linked each of the 170–234 food and beverage items from ENIGHs food diary to the most suitable item in the SHARP-Indicators Database (SHARP-ID) life cycle database to determine each item’s GHGE, that is, the kilograms of carbon dioxide equivalent (kg CO2-eq). This database was developed to quantify the environmental impact of diets in four European countries by using life cycle inventory data from the Agri-footprint 2.0, Ecoinvent 3.3, and CAPRI databases [8]. SHARP-ID includes estimated GHGE values (kg CO2-eq per kilogram of food as eaten) from production to consumption, including food losses and waste at production, preparation, and consumption phases. For foods that were not in the SHARP-ID database, we searched the literature for the most appropriate GHGE factor. We summarized the GHGE values at two levels: first, we summed GHGE values across all purchased items to determine the total dietary GHGE for each household. Second, we summed the values for every food and beverage product (e.g., unsweetened milk, cheese) in each food group (e.g., unsweetened dairy) to determine group-level GHGEs. Then, we estimated the percentage share by specific food groups.

### Food and beverage group-level GHGEs

We classified each food or beverage item in the ENIGH data into 17 mutually exclusive, nutritionally important food groups (Supplementary Table 1): (1) beef, (2) dairy, (3) corn, (4) legumes, (5) beverages, (6) sweet or salty snacks, (7) chicken, (8) oil, (9) vegetables, (10) pork, 11) grains,12) seafood, 13) eggs, 14) fruits, 15) fast food (instant soup, prepared pizzas and a brief category of other prepared foods, such as *tacos*, *tamales*, and hot dogs), 16) seafood, and 17) others.

### Education and urbanicity

We estimated dietary GHGE by educational attainment and urbanicity. We used educational attainment of the head of household (hereafter, education) as a proxy for household SES. We categorized education into 6 mutually exclusive categories: (1) no formal education; (2) completed pre-elementary; (3) completed elementary; (4) completed middle school; (5) completed high school; (6) completed college or higher. Locality size or urbanicity is defined by the National Institute of Statistics and Geography of Mexico according to the number of inhabitants since 1992: rural (< 2,500), small cities (2,500 − 14,999), medium-sized cities (15,000–99,999), and large cities (≥ 100,000) [9].

### Statistical analysis

We first estimated adult equivalent by dividing the recommended dietary allowance (RDA) for energy intake of each household member according to their age and sex by 2,550 kcal, which is the energy intake recommended for an average adult [10]. Then, we estimated the mean total volume of food purchases per adult-equivalent per day (kg/ad-eq/d) and the mean total GHGE per adult-equivalent per day (kg CO2-eq/ad-eq/d) for every survey year to understand the extent to which the changes in GHGE were aligned with changes in food purchases [11]. We obtained the estimations in the overall sample and by education and urbanicity. Then, we estimated the relative contribution for each food group to the total GHGE. Households that did not report food or beverage purchases in a specific item were included in the analysis with a contribution of zero. These same analyses were conducted stratifying by education and urbanicity. Differences were estimated with their confidence intervals of means and proportions, between the last and first survey. All analyses were conducted in Stata 17 using the SVY command, to account for survey design and weights to generate nationally representative results [12]. 

## Results

The proportion of households with the lower education levels decreased over time, while households with the highest education levels increased. From 1992 to 2020, the distribution of households by urbanicity remained stable, with around 50% of the households located in metropolitan cities. (Supplementary Table 2)

### Trends in total GHGE, overall and by education and urbanicity

The volume of food purchases in Mexican households increased linearly over time, from 1.27 (95%CI: 1.24, 1.30) kg/ad-eq/d in 1989 to 2.05 (95%CI: 2.03, 2.07) kg/ad-eq/d in 2020. The beverages group was the one that increased the most, from 0.19 (95%CI 0.17, 0.19) to 0.86 (95%CI 0.84, 0.87) kg/ad-eq/d. Dairy was the group that decreased the most, from 0.20 (95%CI 0.19, 0.21) to 0.13 (95%CI 0.12, 0.13) kg/ad-eq/d in the same period. (Supplementary Table 3) Total GHGE also increased over time, but not linearly: from 3.70 (95%CI: 3.57, 3.82) to 4.90 (95%CI: 4.62, 5.18) kg CO2-eq/ad-eq/d between 1989 and 2014 and stayed relatively stable between 4.63 (95%CI: 4.53, 4.72) and 4.89 (95%CI: 4.81, 4.96) kg CO2-eq/ad-eq/d from 2016 onwards. (Supplementary Table 4)

Across the period studied (1992–2020), the volume of food purchases was higher at higher education levels, however, the gap narrowed over time. The difference in total mean food purchases between college and no education levels was 0.36 (95%CI: 0.35, 0.37) in 1989 and 0.20 (95%CI: 0.19, 0.21) kg in 2020 because of a pronounced increase in food purchases in households with very low levels of education. (Supplementary Table 5) Across the period studied, the volume of food purchases was also higher in households from metropolitan versus rural areas, but households in rural areas increased their food purchases in such a way that the difference was − 0.54 (95%CI: -0.55, -0.52) in 1992 and − 0.15 (95%CI: -0.16, -0.13) kg in 2020. (Supplementary Table 6)

The gap in total GHGE from food purchases across education levels has become smaller over time as a result of a sharp increase in emissions in households with no education or pre-elementary education while GHGE remained stable in households with high school or college. (Supplementary Table 7) Total GHGE increased across all categories of urbanicity but especially in rural areas. In 1992, total GHGE was almost double in metropolitan versus rural areas, while in 2020 total GHGE in metropolitan areas was 18% higher than the observed in rural areas. (Supplementary Table 8)

### Overall trends in the relative contribution of food groups to total GHGE from food purchases (GHGE-FP)

In 1989, the food groups with the highest relative contribution to GHGE-FP were beef (19.89%, 95%CI: 19.18, 20.59), dairy (16.87%, 95%CI: 16.30, 17.42), corn (9.61%, 95%CI: 9.00, 10.22), legumes (7.03%, 95%CI: 6.59, 7.46), and beverages (6.99%, 95%CI: 6.66, 7.32). By 2020, beef was still the top contributor of GHGE-FP but decreased by 2.21 (95%CI: -3.0, -1.42) percentage points (p.p.) compared to 1989. Fast food became the second main contributor of GHGE-FP with 14.17%, increasing 13.44 p.p. (95%CI: 13.16, 13.72) from 1989 to 2020. GHGE-FP of dairy decreased by 5.66 p.p. (95%CI: -6.39, -4.93) from 1989 to 2020, contributing 11.21% (95%CI: 11.06, 11.36) of total GHGE-FP in 2020, followed by beverages (10.09%, 95%CI: 9.94, 10.23), and chicken (10.04%, 95%CI: 9.9, 10.7), which increased 3.10 p.p. (95%CI: 2.58, 3.62) and 4.17 p.p. (95%CI: 3.69, 4.65), respectively, in the same period. The relative contribution of corn and legumes to total GHGE-FP decreased by 4.16 p.p. (95%CI: -4.73, -3.59) and 3.82 p.p. (95%CI: -4.31, -3.33), respectively, from 1989 to 2020 (Table 1, Supplementary Table 9).

**Table 1:**
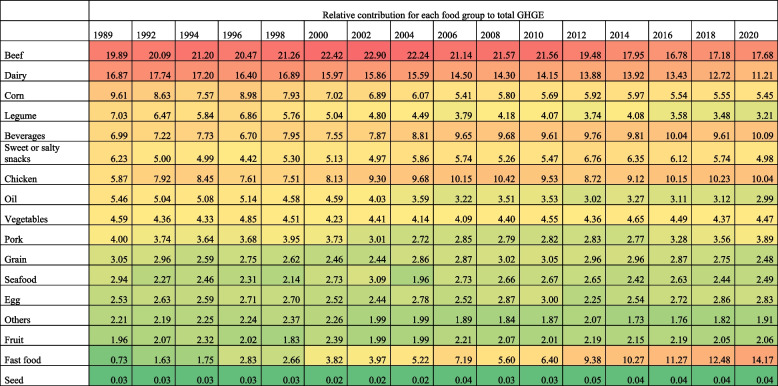
Relative contribution of GHGE by food group in Mexican households, 1989–2020 ENIGHS

### Trends in the relative contribution of food groups to total GHGE-FP stratified by education

In 1989, among households without formal education (Table 2), the food groups with the highest relative contribution to total GHGE-FP were corn (15.81%, 95%CI: 14.07, 17.55), dairy (13.25%, 95%CI: 12.02, 14.47), beef (12.06%, 95%CI: 10.52, 13.59), legumes (11.61%, 95%CI: 10.34, 12.87), and beverages (8.12%, 95%CI: 7.23, 8.99). The food groups with the highest relative increase in GHGE-FP from 1989 to 2020 among these households were fast food (+ 12.56 p.p., 95%CI: 12.30, 12.82) and chicken (+ 5.33 p.p., 95%CI: 4.89, 5.77), while GHGE from dairy (-3.99 p.p., 95%CI: -4.65, -3.33), but also from legumes (-5.53 p.p., 95%CI: -6.15, -4.91), and corn (-5.20 p.p., 95%CI: -5.91, -4.49) decreased relatively. Thus, in 2020, the food groups with the highest relative contribution to total GHGE-FP were fast food (13.08%, 95%CI: 12.23, 13.92), beef (12.24%, 95%CI:11.62, 12.84), corn (10.61%, 95%CI: 10.06, 11.16), and beverages (10.06%, 95%CI:9.55, 10.55).

**Table 2:**
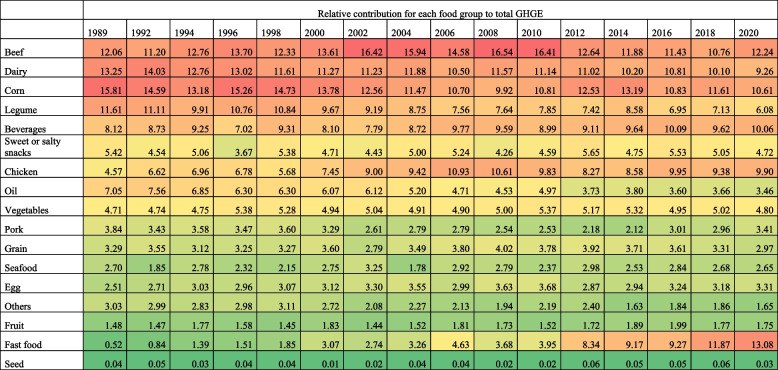
Relative contribution of GHGE by food group among households no formal education, 1989–2020 ENIGHS

Among households with a college degree or more (Table 3), the food groups with the highest relative contribution to total GHGE-FP in 1989 were beef (27.56%, 95%CI: 25.07, 30.04), dairy (24.63%, 95%CI: 22.17, 27.08), sweet or salty snacks (8.07%, 95%CI: 6.69, 9.43), chicken (6.30%, 95%CI: 5.33, 7.25), and beverages (5.86%, 95%CI: 4.7, 7.02). The highest relative increases in GHGE-FP were also from fast food (+ 15.11 p.p, 95%CI: 14.78, 15.44) and chicken (+ 2.78 p.p., 95%CI: 2.29, 3.27), but also beverages (+ 4.90 p.p, 95%CI: 4.42, 5.38), while the GHGE-FP from dairy (-12.83 p.p, 95%CI: -13.66, -12.0), beef (-7.23 p.p, 95%CI: -8.10, -6.36), and sweet or salty snacks (-2.33 p.p, 95%CI: -2.86, -1.80) decreased relatively. Yet, in 2020, beef (20.33%, 95%CI: 19.72, 20.92) was still the top contributor to total GHGE-FP, followed by fast food (16.57%, 95%CI: 15.86, 17.27), dairy (11.80%, 95%CI: 11.38, 12.2), beverages (10.76%, 95%CI: 10.35, 11.16), and chicken (9.08%, 95%CI: 8.72, 9.44). Supplementary Tables 10–15 show estimates for all education levels.

**Table 3:**
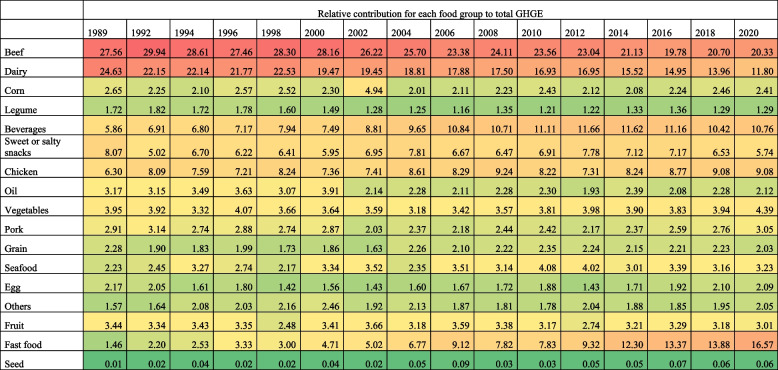
Relative contribution of GHGE by food group among households with a college education, 1989–2020 ENIGHS

### Trends in the contribution of food groups to total GHGE-FP stratified by urbanicity

In 1992, among households in rural areas (Table 4), the food groups with the highest relative contribution to total GHGE-FP were corn (17.29%, 95%CI: 14.95, 19.62), dairy (12.11%, 95%CI: 10.69, 13.51), legumes (11.67%, 95%CI: 10.37, 12.95), beverages (9.29%, 95%CI: 7.71, 10.85), and beef (8.79%, 95%CI:7.34, 10.22). The food groups with the highest relative increase in GHGE-FP from 1992 to 2020 among these households were fast food (+ 12.11 p.p., 95%CI: 11.86, 12.36), chicken (+ 3.60 p.p., 95%CI: 3.13, 4.07) and beef (+ 3.46 p.p., 95%CI: 2.89, 4.03). Only rural areas showed a relative increase in GHGE from beef purchases. The food groups with the highest relative decrease in GHGE-FP were corn (-6.59 p.p., 95%CI: -7.32, -5.86), legumes (-6.18 p.p., 95%CI: -6.8, -5.56), and grains (-1.40 p.p., 95%CI: -1.8, -1.0). Therefore, the food groups with the highest relative contribution to total GHGE-FP in 2020 were fast food (12.49%, 95%CI: 11.99, 12.99), beef (12.25%, 95%CI: 11.91, 12.58), corn (10.70%, 95%CI: 10.25, 11.13), dairy (10.55%, 95%CI: 10.29, 10.79) and beverages (9.66%, 95%CI: 9.36, 9.96).

**Table 4:**
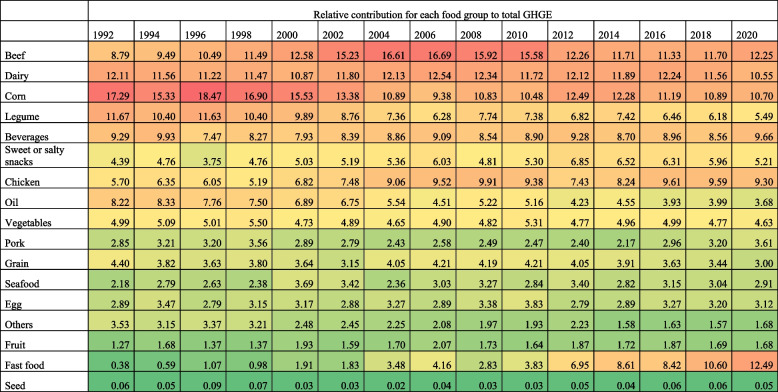
Relative contribution of GHGE by food group among households with a college education, 1989–2020 ENIGHS

Among households in metropolitan areas (Table 5), the food groups with the highest relative contribution to total GHGE-FP in 1992 were beef (26.17%, 95%CI: 25.17, 27.17), dairy (19.97%, 95%CI: 19.16, 20.76), chicken (9.11%, 95%CI: 8.63, 9.58), beverages (6.86%, 95%CI: 6.37, 7.35), and sweet or salty snacks (5.22%, 95%CI: 4.69, 5.74). Among these households, the highest relative increase in GHGE-FP from 1992 to 2020 were fast food (+ 12.38 p.p., 95%CI: 12.03, 12.73), beverages (+ 3.70 p.p., 95%CI: 3.19, 4.21), and chicken (+ 1.02 p.p., 95%CI: 0.45, 1.59). In contrast, GHGE-FP relative decreased from dairy (-8.38 p.p., 95%CI: -9.15, -7.61), beef (-5.78 p.p., 95%CI: -6.64, -4.92), legumes (-1.72 p.p., 95%CI: -2.09, -1.35), and corn (-1.04 p.p., 95%CI: -1.44, -0.64 p.p.). Yet, in 2020, beef (20.39%, 95%CI: 20.04, 20.73) was still the top contributor to total GHGE-FP, followed by fast food (14.46%, 95%CI: 14.06, 14.84), dairy (11.59%, 95%CI: 11.36, 11.81), beverages (10.56%, 95%CI: 10.34, 10.77), and chicken (10.13%, 95%CI: 9.93, 10.32). Supplementary Tables 16–19 show estimates for all urbanicity strata.

**Table 5:**
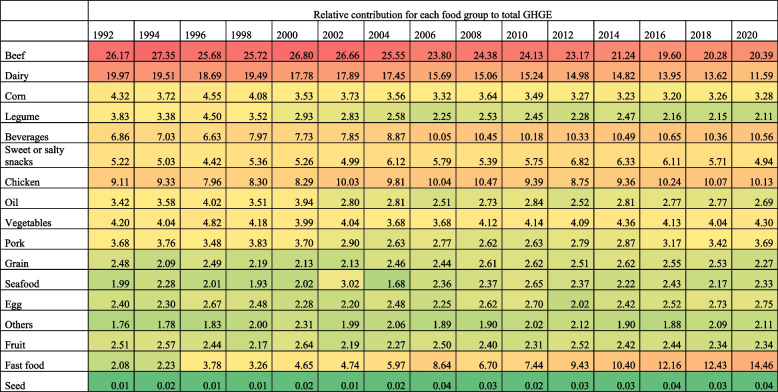
Relative contribution of GHGE by food group among households with a college education, 1989–2020 ENIGHS

## Discussion

Our study improves the understanding of the changes in GHGE from food and beverage purchases among Mexican households from 1989 to 2020, overall and by sociodemographic variables. The top 3 contributors to GHGE in 1989 were beef, dairy, and corn. By 2020, beef and dairy were also top contributors, and fast food replaced corn.

Overall, dietary GHGE increased over time in Mexico, from 3.70 kg in 1989 to 4.90 CO2-eq/ad-eq/d in 2014. From 2014 onwards, GHGE remained stable ranging from 4.63 to 4.89 kg CO2-eq/ad-eq/d. Similarly, a study conducted in metropolitan areas in Brazil found an increase in GHGE by food purchases from 1987 to 1988 to 1995–1996, from ~ 1550 to ~ 1800 g CO2-eq/1000 kcal/d; the value in the next period (2002–2003) was ~ 1600 g CO2-eq/1000 kcal/d, and finally in 2017–2018 was ~ 1900 g CO2-eq/1000 kcal/d) [13]. Different patterns have been observed in other countries. In Beijing, dietary GHGE increased from 2.15 kg CO2-eq/per capita/d in 1980 to ~ 3.60 kg CO2-eq/per capita/d in 2006 before slightly declining to 3.04 kg CO2-eq/per capita/d in 2017 [14]. In contrast, in the US, Sweden, and Spain, dietary GHGE has decreased over time: from 4.02 to 2.45 kg CO2-eq/per capita/d in the 2003–2018 period in the US, from 3.42 to 2.48 kg CO2-eq/per person/d from 2001 to 2004 to 2014–2018 in Sweden, and from 2.81 to 2.38 CO2-eq/per person/d in the 2008–2017 period in Spain [15–17]. The increasing trends and high levels observed in Mexico compared to other countries are concerning. Overall differences across countries result from changes in dietary consumption and can also reflect very different levels and changes over time in different sociodemographic groups. Future studies will be needed to further understand the rationale of such changes.

Although households with higher education levels and those in more urbanized areas contributed more to dietary GHGE than lower education and more rural households in Mexico across the period studied, households with lower education level and those living in rural areas showed the highest increase in these emissions from 1989 to 2020, reducing the gap between education levels and rural/urban areas. These trends were in line with changes in the volume of food purchases over time by education and urbanicity, which could indicate that the increases in dietary GHGE are at least partially explained by the rise in food purchases. Unlike our study, Bassi et al., using data from the National Health and Nutrition Surveys from 2003 to 2018, found small differences in dietary GHGE across time among US adults with different SES. The authors posit that the small difference across SES groups can be explained by the fact that beef is the main contributor to dietary GHGE, and in this population, the consumption of meat is not influenced by SES [15]. 

As in other countries [14–16], meat, specifically beef, is the main contributor to total dietary GHGE in Mexico, although the contribution of beef has declined somewhat over time. The most striking increase in dietary GHGE contribution was from fast food, which in 1989 was one of the food groups that least contributed to GHGE, while in 2020, it was the second contributor. The increase in GHGE from chicken was also notable. On the other hand, corn and legumes were the food groups that reduced most of their contribution to GHGE from 1989 to 2020. Except for Sweden, previous studies from other countries focused on analyzing the contribution of specific or broad food categories or the main food contributors to dietary GHGE; therefore, none of these studies analyzed a category of fast food. In a randomly selected sample of adults from Gothenburg, Sweden, the consumption of fast food decreased by around 15 kg/person/year from 2001 to 2004 to 2014–2018, resulting in a discrete decrease in GHGE by this food type of around 5 kg CO2-eq/person/year in the same period [16]. In Mexico, the increased contribution of fast food to dietary GHGE is part of the well-established nutrition transition characterized by a shift to diets high in saturated fat, sodium, sugar, and refined carbohydrates [18]. Therefore, the nutrition transition can have not only negative effects on human health but also on the environment.

Our results by education level are also in line with the nutrition transition model, which indicates that such transition can differ by SES and urbanization. We observed that fast food displaced corn as the main contributor to dietary GHGE among households with lower education levels. It was also notable that legumes were not among the top contributors to dietary GHGE in 2020 in comparison to 1989. Among households with the highest education level, beef was the main GHGE contributor from 1989 to 2020, although its contribution decreased over time. Also, fast food displaced dairy as the second contributor to dietary GHGE in this group. The differences observed between rural and metropolitan areas were similar to the differences observed by education. These findings suggest that the nutrition transition affected higher education levels and more urbanized areas first, as observed in other countries [18, 19], but now the entire population is equally affected.

Our finding that beef is the main contributor of GHGE regardless of education can be explained by the fact that consumption decreased among households with higher levels of education, but remained stable among households with lower education over time. Theoretically, higher education is not only related to greater access to a variety of foods, it is also related to nutritional knowledge [20, 21]. Social theory suggests that compared to those with lower levels of education, people with higher levels of education are the first to adopt new recommendations. Since 2015, the Mexican Dietary Guidelines recommend limiting the consumption of red meat for cancer prevention [22], while the 2023 Healthy and Sustainable Dietary Guidelines for the Mexican population recommend the reduction of red meat intake for human and planetary health [23]. Future analyses will be needed to understand the potential impact of the current dietary guidelines on food patterns and their contribution to GHGE.

The increase of dietary GHGE by fast food over time across education strata can be explained by different reasons to those provided for beef. First, it is important to consider that the category of fast food includes traditional Mexican fast food, which is widely consumed by the general population [24]. However, it would be expected that the consumption of traditional Mexican fast food does not change over time. Therefore, increases in fast food consumption and in its contribution to GHGE across education strata might be explained by the consumption of Western fast food. Unfortunately, this cannot be explored because fast food in the ENIGH only includes three items and one of them englobes several traditional and non-traditional Mexican fast foods. More research will be needed to better understand why fast food has increased its contribution to GHGE regardless of education.

Our study has limitations, especially those related to dietary GHGE estimation. First, we used a database developed to estimate the dietary carbon footprint in European countries, one of the most complete public databases available at the time of our analyses. It is likely that with globalization, food systems (and associated GHGE emissions) are becoming more similar across countries. Similar estimations of dietary GHGE were obtained by Curi-Quinto et al. [25] in cross-sectional data, despite using different GHGE sources. However, important local differences in the GHGE linked to specific foods may still exist and can impact the validity of our estimates. Future studies using more comprehensive GHGE estimations, such as those recently published by Scarborough et al. [26], will increase our understanding of the contribution of the food systems to total GHGE. Second, we assumed the GHGE contribution of each food was the same over time since there is no information on the dietary carbon footprint for different years. This is a strong assumption considering that supply chains and other factors can vary over time and be different by food type, political, and socioeconomic factors, among others [27]. Specifically, fast food product offerings may have increased, which could partially explain the rise in their relative contribution to dietary GHGE over time. Yet, we could not analyze this category in detail given the limited items included in ENIGH. Third, we cannot rule out the possibility that dietary GHGE estimates for 2020 were atypical given the COVID-19 pandemic, in particular, the GHGE for fast food. Finally, the ENIGH surveys added food items over time. The added items were derived by disaggregating large food groups into small groups (i.e., beef was disaggregated into flank steak, breaded beef, and beef steak). Thus, we believe that this would have minimal impacts on our estimates. However, it is important to note that starting in 2006, ENIGH has used the same methodology, with no new items added. Trends from 2006 onwards seem to be consistent with estimates from previous years, strengthening our belief that results are comparable over time.

## Conclusion

This study suggests that less healthy and sustainable foods and beverages, such as fast food and beverages, have replaced more healthy and sustainable foods in Mexico, such as corn and legumes, resulting in an increase in GHGE, especially amongst the more vulnerable subgroups of the population. Scientific evidence related to the impact of food systems on the environment has served to update recommendations to the population, such as the dietary guidelines. Our findings provide empirical evidence on the food groups that may require focus to comply with the recommendations established in the recently published 2023 Dietary Guidelines for the Mexican Population [23], which seek to improve the population’s health, prevent diseases, and protect the planet.

### Supplementary Information

Supplementary Material 1.

## Data Availability

Raw data available: Data on food and beverage purchases in Mexican households is available at: https://en.www.inegi.org.mx/programas/enigh/nc/2022/Data on greenhouse gas emissions is available at: https://lifesciences.datastations.nl/dataset.xhtml? persistentId=doi:10.17026/dans-xvh-x9wz.

## Abbreviations

GHGE: Greenhouse Gas Emissions
SES: Socioeconomic Status
ENIGH: National Income and Expenditure Survey
SHARP-ID: SHARP- Indicators Database
RDA: Recommended Dietary Allowance
GHGE-FP: Greenhouse Gas Emissions from food purchases
