# Supplementary material for: Trends in the contribution of greenhouse gas emissions from food and beverage purchases in Mexico: 1989–2020
Source: Nutr J. 2024 May 18;23:55. doi: 10.1186/s12937-024-00955-z (PMC11102158; doi:10.1186/s12937-024-00955-z)
Supplement: Supplementary file 1 — Supplementary Material 1. [file 12937_2024_955_MOESM1_ESM.pdf]

Supplementary table	Description	Page
1	Overtime food items and GHGE factors	2
2	Descriptive characteristics in Mexican households, 1989, 2020 ENIGHS.	8
3	Purchases by food group per equivalent adult in kilograms, 1989, 2020 ENIGHS	9
4	GHGE by food group per equivalent adult in kilograms of CO_2, 1989, 2020 ENIGHS	11
5	Purchases by educational level of the head of household in kilograms per equivalent adult, 1989, 2020 ENIGHS	13
6	Purchases by urbanicity in kilograms per equivalent adult, 1992, 2020 ENIGHS	14
7	GHGE by educational level of the head of household per equivalent adult in kg of CO_2, 1989, 2020 ENIGHS	15
8	GHGE by urbanicity per equivalent adult in kg of CO_2, 1992, 2020 ENIGHS	16
9	Relative contribution of GHGE by food group in Mexican households, 1989, 2020 ENIGHS	17
10	Relative contribution of GHGE by food group among households with no formal education, 1989, 2020 ENIGHS.	19
11	Relative contribution of GHGE by food group among households with completed pre elementary education, 1989, 2020 ENIGHS.	21
12	Relative contribution of GHGE by food group among households with completed elementary education, 1989, 2020 ENIGHS.	23
13	Relative contribution of GHGE by food group among households with completed middle school, 1989, 2020 ENIGHS.	25
14	Relative contribution of GHGE by food group among households with completed high schooll education, 1989, 2020 ENIGHS.	27
15	Relative contribution of GHGE by food group among households with completed college education, 1989, 2020 ENIGHS.	29
16	Relative contribution of GHGE by food group among households living in rural aeras, 1992, 2020 ENIGHS.	31
17	Relative contribution of GHGE by food group among households living in small aeras, 1992, 2020 ENIGHS.	33
18	Relative contribution of GHGE by food group among households living in medium aeras, 1992, 2020 ENIGHS.	35
19	Relative contribution of GHGE by food group among households living in metropolitan aeras, 1992, 2020 ENIGHS.	37

Supplementary Table 1. Overtime food items and GHGE factors

	Food	Food group	1989, 1992	1994, 2000	2002, 2004	2006, 2020	Factor	SHARP ID
1	Corn in grain (of all types and colors)	Corn	YES	YES	YES	YES	0.522	A000T
2	Corn flour, cornstarch, starch, nixtamalized for tortillas, for atole and flour for tamales	Corn	YES	YES	YES	YES	0.992	A002Q
3	Corn dough (of all types and colors)	Corn	YES	YES	YES	YES	0.992	A002Q
4	Corn tortilla (of all types and colors)	Corn	YES	YES	YES	YES	0.419	Guzman Soria[1]
5	Tostadas, raspadas, tostitos, tortilla chips, tlayudas	Corn	NO	YES	YES	YES	0.419	Guzman Soria[1]
6	Other corn products (except cereal)	Corn	YES	YES	YES	YES	0.419	Guzman Soria[1]
7	Wheat flour	Grains	YES	YES	YES	YES	0.568	A003X
8	Flour tortilla	Grains	YES	YES	YES	YES	0.419	Guzman Soria[1]
9	Pasta for soup	Grains	YES	YES	YES	YES	0.456	A007L
10	Sweet cookies	Sweet or salty	YES	YES	YES	YES	3.124	A009V
11	Pretzels	Sweet or salty	YES	YES	YES	YES	0.966	A005Y
12	White bread: bolillo, telera, baguette, etc.	Grains	YES	YES	YES	YES	0.966	A004Y
13	Sweet bread in pieces (of all kinds)	Sweet or salty	YES	YES	YES	YES	3.124	A009V
14	Packaged sweet bread	Sweet or salty	NO	NO	NO	YES	3.124	A009V
15	Bread for sandwich, hamburger, hot dog and toast	Grains	NO	YES	YES	YES	0.966	A0BB2
16	Cakes and cakes in pieces or in bulk, homemade cake	Sweet or salty	YES	YES	YES	YES	12.428	A00AN
17	Packaged cakes and cupcakes	Sweet or salty	NO	NO	NO	YES	12.428	A00AN
18	Wheat grain, other wheat products (except cereal)	Grains	YES	YES	YES	YES	1.062	A001D
19	Grain rice	Grains	YES	YES	YES	YES	1.062	A001D
20	Other rice products	Grains	YES	YES	YES	YES	0.845	A003F
21	Corn cereal, wheat, rice, oats, granola, etc.	Grains	NO	YES	YES	YES	1.044	A00CV
22	Snacks: chips, popcorn, cheetos, doritos, etc. (except paps)	Sweet or salty	NO	YES	YES	YES	0.667	A00DC
23	Instant soup	Fast food	NO	NO	YES	YES	6.321	A0B9R
24	Other cereals (except cereal)	Grains	YES	NO	YES	YES	1.4	Average (barley and amaranth)
25	Beef Steak (anywhere pulled)	Beef	YES	YES	YES	YES	34.041	A020Z
26	Flank steak, fillet	Beef	NO	NO	YES	YES	34.041	A020Z
27	Breaded beef	Beef	NO	NO	YES	YES	34.041	A020Z
28	Beef shank	Beef	NO	NO	YES	YES	34.041	A020Z
29	Beef Rib Chop	Beef	YES	YES	YES	YES	34.041	A020Z
30	Agujas, aldilla, shank, tithe, backbone, beef strip for roasting, remnant, tampiqueña	Beef	NO	NO	YES	YES	34.041	A020Z
31	Beef stew	Beef	YES	YES	YES	YES	34.041	A020Z
32	Special cuts of beef	Beef	YES	YES	YES	YES	34.041	A020Z
33	Beef Burgers for Grilling	Beef	NO	NO	YES	YES	34.041	A049S
34	Ground beef	Beef	NO	NO	YES	YES	34.041	A049S
35	Chunk beef pulp	Beef	YES	YES	YES	YES	34.041	A020Z
36	Meat from other parts of the beef	Beef	NO	NO	YES	YES	34.041	A020Z
37	Beef innards	Beef	YES	YES	YES	YES	34.041	A01ZM
38	Pork steak (from whatever part is taken)	Pork	YES	YES	YES	YES	12.101	A01ZP
39	Piece of pork leg	Pork	NO	NO	YES	YES	12.101	A021G
40	Chunk pork pulp	Pork	YES	YES	YES	YES	12.101	A01RG
41	Ground pork	Pork	NO	NO	YES	YES	12.101	A01RG

42	Rib and pork chop	Pork	YES	YES	YES	YES	12.101	A01ZP
43	Pork shoulder	Pork	NO	NO	YES	YES	12.101	A01RG
44	Pork knuckle	Pork	NO	NO	YES	YES	12.101	A01ZP
45	Meat from other parts of the pork	Pork	NO	NO	YES	YES	12.101	A01RG
46	Pork offal	Pork	YES	YES	YES	YES	12.101	A01YM
47	Beef enchilada	Beef	NO	YES	YES	YES	18.566	A01QR
48	Pork rind	Beef	NO	NO	YES	YES	12.101	A01RG
49	Chorizo with any seasoning and color and sausage	Beef	YES	YES	YES	YES	12.101	A024J
50	Smoked Pork Chop	Beef	NO	NO	YES	YES	18.566	A01QR
51	Machaca and dried meat	Beef	YES	YES	YES	YES	18.566	A01QR
52	Pork ham	Beef	YES	YES	YES	YES	10.24	A022T
53	Mortadella, pork cheese and salami, assorted meat bologna	Beef	YES	YES	YES	YES	12.101	A024X
54	Processed lardo (bacon)	Beef	YES	YES	YES	YES	10.245	A022X
55	Sausages and salami	Beef	YES	YES	YES	YES	12.101	A024F
56	Other processed meats, leather	Beef	NO	NO	YES	YES	12.101	A01RG
57	Bone, in chicken leg, thigh or breast	Chicken	YES	YES	YES	YES	8.582	A01SP
58	Boneless chicken leg, thigh or breast	Chicken	NO	NO	YES	YES	8.582	A01SP
59	Whole chicken or pieces (except leg, thigh and breast)	Chicken	YES	YES	YES	YES	8.582	A01SP
60	Viscera and other parts of the chicken	Chicken	YES	YES	YES	YES	8.582	A020F
61	Other birds	Chicken	YES	YES	YES	YES	8.582	A01SQ
62	Chicken chorizo, ham and nugget, sausage, mortadella, etc.	Beef	NO	NO	YES	YES	12.101	A024F
63	Sheep and ram	Others	YES	YES	YES	YES	27.389	A01RL
64	Goat and kid	Others	YES	YES	YES	YES	27.389	A01RL
65	Other meats: horse, rabbit, iguana, wild boar, frog, turtle, deer	Others	YES	YES	YES	YES	10.341	Average (beef and chicken)
66	Clean and Uncleaned Whole Fish	Seafood	YES	YES	YES	YES	11.621	A026V
67	Fish steak	Seafood	NO	NO	YES	YES	11.621	A026V
68	Canned tuna	Seafood	YES	YES	YES	YES	7.931	A0FBT
69	Processed cod and salmon	Seafood	NO	NO	YES	YES	4.648	A0C75
70	Smoked, dried fish, nuggets, sardines, etc.	Seafood	NO	NO	YES	YES	17.524	A02KH
71	Eels, eels, fish roe, manta ray, gar, etc.	Seafood	NO	NO	YES	YES	8.375	A02DQ
72	Fresh shrimp	Seafood	NO	NO	NO	YES	15.975	A02GB
73	Fresh seafood	Seafood	NO	NO	YES	YES	11.621	A026T
74	Processed seafood	Seafood	NO	NO	YES	YES	11.621	A026T
75	Pasteurized cow's milk	Dairy	YES	YES	YES	YES	1.585	A02LT
76	Condensed milk	Dairy	YES	YES	YES	YES	4.502	A02PG
77	Evaporated milk	Dairy	YES	YES	YES	YES	5.586	A02PF
78	Whole or skimmed powdered milk	Dairy	YES	YES	YES	YES	7.764	A02PJ
79	Modified or formula milk	Dairy	YES	YES	YES	YES	7.764	A02PJ
80	Unpasteurized milk (rough milk)	Dairy	YES	YES	YES	YES	1.585	A02LV
81	Other milk: donkey, goat, soy	Dairy	YES	YES	YES	YES	3.046	A02MB
82	Sliced or spreadable yellow cheese	Dairy	YES	YES	YES	YES	13.142	A02QE
83	Aged cheese and cotija	Dairy	YES	YES	YES	YES	13.142	A02QE
84	Chihuahua cheese	Dairy	YES	YES	YES	YES	13.142	A02QE
85	Fresh cheese	Dairy	YES	YES	YES	YES	13.142	A02QE
86	Manchego cheese	Dairy	YES	YES	YES	YES	13.142	A02QE
87	Oaxaca or asadero cheese	Dairy	YES	YES	YES	YES	13.142	A02QE
88	Other cheeses	Dairy	YES	YES	YES	YES	13.142	A02QE

89	Cream	Oil	YES	YES	YES	YES	10.682	A02MK
90	Butter	Oil	YES	YES	YES	YES	1.59	A02MV
91	Fermented milk drinks	Beverages	YES	YES	YES	YES	1.73	A02NE
92	Other milk derivatives	Oil	NO	NO	YES	YES	2.2	HEAlabel[2]
93	White and red chicken egg	Eggs	YES	YES	YES	YES	1.903	A031G
94	Other eggs: quail, leg, turkey, etc.	Eggs	YES	YES	YES	YES	1.903	A031G
95	Vegetable oil: canola, safflower, sunflower, corn, etc.	Oil	YES	YES	YES	YES	3.518	A036Y
96	Coconut, olive, soy oil	Oil	YES	YES	YES	YES	3.791	A036P
97	Margarine	Oil	YES	YES	YES	YES	4.26	A0F1G
98	Lard	Oil	YES	YES	YES	YES	11.515	A037V
99	Vegetable shortening	Oil	YES	YES	YES	YES	3.858	A036N
100	Other oils: cod, shark, turtle, jerk	Oil	NO	NO	YES	YES	4.954	A038Q
101	Beets and sweet potatoes	Grains	YES	YES	YES	YES	0.513	A00QG
102	Potato	Grains	YES	YES	YES	YES	0.503	A00ZT
103	Radish	Grains	NO	NO	YES	YES	0.173	A00QV
104	Other tubers	Grains	YES	YES	YES	YES	0.503	A00ZT
105	Flour for mashed potatoes	Grains	YES	YES	YES	YES	0.939	A011G
106	Potato chips in bag or in bulk	Grains	NO	YES	YES	YES	1.855	A00FC
107	Chard, spinach and purslane, quintolines, quelites and pipica	Vegetables	YES	YES	YES	YES	0.6525	A00MJ
108	Avocado	Vegetables	YES	YES	YES	YES	1.34	A01LB
109	Garlic	Vegetables	NO	YES	YES	YES	0.382	A00GZ
110	Broccoli	Vegetables	NO	NO	YES	YES	0.985	A00FN
111	Zucchini and pumpkin	Vegetables	YES	YES	YES	YES	0.581	A00KH
112	Onion	Vegetables	YES	YES	YES	YES	0.423	A00HC
113	Squash	Vegetables	YES	YES	YES	YES	0.538	A00JS
114	Pea	Vegetables	YES	YES	YES	YES	0.243	A01DP
115	Jalapeno pepper	Vegetables	YES	YES	YES	YES	0.66	CLUNE STEPHEN[3]
116	Poblano pepper	Vegetables	YES	YES	YES	YES	0.66	CLUNE STEPHEN[3]
117	Serrano chili	Vegetables	YES	YES	YES	YES	0.66	CLUNE STEPHEN[3]
118	Other chillies	Vegetables	YES	YES	YES	YES	0.66	CLUNE STEPHEN[3]
119	Cilantro	Vegetables	YES	YES	YES	YES	0.729	A00YE
120	Cabbage and cabbage	Vegetables	YES	YES	YES	YES	0.452	A00GC
121	Green bean	Vegetables	YES	YES	YES	YES	0.44	HEAlabel[2]
122	Corn	Corn	YES	YES	YES	YES	2.559	A00KP
123	Epazote	Vegetables	YES	YES	YES	YES	0.729	A00YE
124	Tomato	Vegetables	YES	YES	YES	YES	0.919	A0DMX
125	Lettuce	Vegetables	YES	YES	YES	YES	326	A00KX
126	Prickly pear	Vegetables	YES	YES	YES	YES	0.562	VERGEL RANGEL[4]
127	Cucumber	Vegetables	YES	YES	YES	YES	3.861	A00JM
128	Parsley and mint	Vegetables	YES	YES	YES	YES	0.729	A00YE
129	Green tomato	Vegetables	YES	YES	YES	YES	0.919	A00HQ
130	Carrot	Vegetables	YES	YES	YES	YES	0.391	A00QH
131	Other vegetables, papalo, olives, squash flower, chaya	Vegetables	NO	NO	YES	YES	1.363	A00FJ
132	Corn, soybean, wheat, alfalfa sprouts	Vegetables	NO	NO	YES	YES	0.59	Average (wheat germ and soybeans sprouts)
133	Packaged chillies	Vegetables	YES	YES	YES	YES	0.66	CLUNE STEPHEN[3]
134	Dried or powdered chili	Vegetables	YES	YES	YES	YES	0.66	CLUNE STEPHEN[3]
135	Packaged vegetables and legumes, olives, pickled vegetables	Vegetables	YES	YES	YES	YES	2.191	A0ETQ

136	Frozen vegetables and legumes	Vegetables	NO	YES	YES	YES	1.363	A00FJ
137	Bean grain	Legumes	YES	YES	YES	YES	1.109	A012S
138	Chickpea grain	Legumes	YES	YES	YES	YES	1.141	A013M
139	Yellow or green broad beans Lentils	Legumes	YES	YES	YES	YES	0.961	A013H
140	Lentil grain	Legumes	NO	NO	YES	YES	0.669	A013Q
141	Other grain legumes	Legumes	NO	NO	YES	YES	0.476	A012G
142	Processed bean	Legumes	YES	YES	YES	YES	1.228	A01BB
143	Other processed legumes	Legumes	YES	YES	YES	YES	1.228	A01BB
144	Bulk seeds	Seeds	YES	YES	YES	YES	1.41	CLUNE STEPHEN[3]
145	Packaged seeds	Seeds	YES	YES	YES	YES	1.41	CLUNE STEPHEN[3]
146	Processed seeds, textured soybeans	Seeds	NO	NO	YES	YES	1.41	CLUNE STEPHEN[3]
147	Custard apple, custard apple, soursop	Fruits	NO	NO	YES	YES	0.83	Average (all fruits)
148	Cherry, raspberry, strawberry, blackberry	Fruits	YES	YES	YES	YES	0.51	A01EA
149	Apricot, peach, peach	Fruits	YES	YES	YES	YES	0.323	A01GL
150	Chicozapote and mamey	Fruits	YES	YES	YES	YES	0.83	Average (all fruits)
151	Plum and jobo	Fruits	NO	NO	YES	YES	0.184	A01GQ
152	Guava	Fruits	YES	YES	YES	YES	0.28	CLUNE STEPHEN[3]
153	Lime	Fruits	NO	NO	YES	YES	0.347	A01CA
154	Lemon	Fruits	YES	YES	YES	YES	0.488	A01BY
155	Tangerine, nectarine, tangerine	Fruits	YES	YES	YES	YES	0.46	A01CD
156	Pink grapefruit	Fruits	NO	NO	YES	YES	0.992	A01CY
157	Mango	Fruits	YES	YES	YES	YES	1.438	A01LF
158	Apple and peron	Fruits	YES	YES	YES	YES	0.742	A01DJ
159	Melon	Fruits	YES	YES	YES	YES	0.381	A00KF
160	Orange	Fruits	YES	YES	YES	YES	0.738	A01CR
161	Papaya	Fruits	YES	YES	YES	YES	1.474	A01LG
162	Pear	Fruits	YES	YES	YES	YES	0.243	A01DP
163	Pineapple	Fruits	YES	YES	YES	YES	0.466	A01LP
164	Pitahaya and tuna	Fruits	NO	NO	YES	YES	2.15	CLUNE STEPHEN[3]
165	Plantain and castilla	Fruits	NO	NO	YES	YES	0.72	CLUNE STEPHEN[3]
166	Green banana and tabasco	Fruits	YES	YES	YES	YES	0.72	CLUNE STEPHEN[3]
167	Other plantains (Chiapas, Dominico, Guinea, Manzano, Dorado, Portalimón and Roatan)	Fruits	YES	YES	YES	YES	0.72	CLUNE STEPHEN[3]
168	Watermelon	Fruits	YES	YES	YES	YES	0.367	A00KJ
169	Grape	Fruits	YES	YES	YES	YES	0.991	A01CY
170	Other fruits: garambullo, pomegranate, fig, jicama, kiwi, etc., fruit arrangement	Fruits	NO	NO	YES	YES	0.509	A01HG
171	Fruits in syrup and preserves	Fruits	YES	YES	YES	YES	2.435	A01PS
172	Crystallized, enchiladas and dried fruits	Fruits	YES	YES	YES	YES	1.964	A01MA
173	White and brown sugar	Others	YES	YES	YES	YES	0.6	A032J
174	Honey bee	Others	YES	YES	YES	YES	1.181	A033J
175	Other sugars and honeys	Others	YES	YES	YES	YES	0.6	A032Q
176	Ground roasted coffee beans	Beverages	YES	YES	YES	YES	0.572	A03GN
177	Instant roasted coffee, cappuccino	Beverages	YES	YES	YES	YES	0.572	A03KE
178	Flower and leaves for tea	Beverages	YES	YES	YES	YES	0.699	A03LC
179	Soluble tea (any flavor)	Beverages	YES	YES	YES	YES	0.699	A03LB
180	Tablet chocolate	Others	YES	YES	YES	YES	5.994	AOEQD
181	Powdered chocolate	Others	NO	NO	YES	YES	5.994	AOEQD

182	Other chocolates	Others	YES	YES	YES	YES	5.994	AOEQD
183	Cinnamon	Others	YES	YES	YES	YES	1.6	HEAlabel[2]
184	Clove	Others	NO	NO	YES	YES	1.6	HEAlabel[2]
185	Scented herbs	Others	NO	NO	YES	YES	0.1	HEAlabel[2]
186	Chicken, tomato, shrimp and beef concentrates	Others	YES	YES	YES	YES	0.1	HEAlabel[2]
187	Mayonnaise	Others	YES	YES	YES	YES	4.12	A044X
188	Mole into paste or powder	Others	YES	YES	YES	YES	0.1	A043F
189	Mustard	Others	YES	YES	YES	YES	0.1	A044G
190	Pepper	Others	YES	YES	YES	YES	0.1	HEAlabel[2]
191	Salt	Others	YES	YES	YES	YES	0.1	A042P
192	Sweet and spicy sauces	Others	YES	YES	YES	YES	0.1	HEAlabel[2]
193	Vinegar	Others	YES	YES	YES	YES	0.1	A044L
194	Other dressings, spices and sauces, vanilla	Others	YES	YES	YES	YES	2	HEAlabel[2]
195	Rice cereal, oatmeal, banana, apple, mixed for baby	Grains	NO	NO	YES	YES	1.044	A00CV
196	Baby food	Grains	YES	YES	YES	YES	1.32	A00EQ
197	Fruit and vegetable juices of any combination for babies	Beverages	YES	YES	YES	YES	1.44	A03DB
198	Prepared pizzas	Fast food	YES	YES	YES	YES	5.561	A03ZN
199	Carnitas	Beef	YES	YES	YES	YES	17.648	A03VV
200	Roasted chicken	Chicken	YES	YES	YES	YES	8.582	A01SP
201	Barbecue and birria	Others	YES	YES	YES	YES	17.648	A03VV
202	Other prepared foods: atole, flautas, stews, hot dogs, soups, tacos, tamales, cakes, sopes, menudo, pozole, smoothies, jellies, corn, etc.	Fast food	YES	YES	YES	YES	8.073	A03ZK
203	Fresh mushrooms: mushrooms, huitlacoche and mushrooms	Vegetables	NO	NO	YES	YES	4.927	A0ETG
204	Insects: grasshoppers, chinicuiles, escamoles, maguey worms, ants (chicatana), jumiles	Others	YES	YES	YES	YES	0.1	Low
205	Flans, jellies and powdered puddings	Sweet or salty	YES	YES	YES	YES	12.428	A00BX
206	Caramels, milk caramels, jamoncillos and custard	Sweet or salty	YES	YES	YES	YES	2.127	A02PX
207	Ates, peanut butter, jellies, jam	Sweet or salty	YES	YES	YES	YES	2.435	A01MM
208	Ice creams, ice creams and popsicles	Sweet or salty	YES	YES	YES	YES	10.568	A02PZ
209	Other sweets, basket of sweets	Sweet or salty	YES	YES	YES	YES	0.767	A0EQQ
210	Nixtamal grinding	Corn	YES	YES	YES	YES	0.992	A002Q
211	Other expenses related to food preparation, lime for nixtamal	Not included	..	..	..	..	..	..
212	Packaged food and/or drinks, food pantry provided by other households	Not included	..	..	..	..	..	..
213	Pet food	Not included	..	..	..	..	..	..
214	Animal feed for home use	Not included	..	..	..	..	..	..
215	Bottled natural water	Beverages	YES	YES	NO	YES	0.272	A03DT
216	Mineral water, quina, demineralized with or without flavor	Beverages	YES	YES	YES	YES	0.272	A03DQ
217	Prepared water and natural juices	Beverages	NO	NO	YES	YES	0.514	A03GE
218	Packaged juices and nectars	Beverages	YES	YES	YES	YES	0.272	A03DY
219	Concentrates and powders to prepare beverages	Beverages	NO	YES	YES	YES	0.514	A03GE
220	Cola and flavored soft drinks	Beverages	YES	YES	YES	YES	0.514	A03DZ
221	Energy drink	Beverages	NO	NO	YES	YES	0.514	A03GA
222	Ice, natural syrup, lechugilla, sangrita, tascalate	Others	YES	YES	YES	YES	0.1	Low
223	cognac and brandy	Others	YES	YES	YES	YES	1.153	A03PE
224	Beer	Others	YES	YES	YES	YES	0.43	A03MB
225	Anise (liquor)	Others	NO	NO	YES	YES	1.055	A03NS
226	Sherry	Others	NO	NO	YES	YES	1.055	A03NS
227	Fruit liqueur or creams	Others	NO	NO	YES	YES	1.055	A03NS

228	Mead, pulque, tlachique, tepache, tuba and fermented corn drinks	Others	YES	YES	YES	YES	1.055	A03NS
229	Aguardiente, cane alcohol, charanda, mezcal	Others	YES	YES	YES	YES	1.055	A03NS
230	Aged rum, white, with lemon	Others	YES	YES	YES	YES	1.292	A03PJ
231	Eggnog	Others	NO	NO	YES	YES	1.397	A03PS
232	white and rosé cider	Others	NO	NO	YES	YES	0.959	A03ND
233	Añejo, blue and white tequila	Others	YES	YES	YES	YES	1.055	A03NS
234	White, rosé, red table wine	Others	YES	YES	YES	YES	1.397	A03MT
235	Vodka	Others	YES	YES	YES	YES	1.224	A03PH
236	Whiskey	Others	YES	YES	YES	YES	1.272	A03PF
237	Prepared alcoholic drink	Others	YES	YES	YES	YES	1.292	A03PN
238	Other alcoholic beverages: champagne	Others	NO	NO	YES	YES	1.291	A03NA

Supplementary table 2. Descriptive characteristics in Mexican households, 1989, 2020 ENIGHS.

	1989	1992	1994	1996	1998	2000	2002	2004	2006	2008	2010	2012	2014	2016	2018	2020
n, households	11051	10075	12389	13708	10577	9792	16769	22029	20331	29034	27233	8899	19347	69750	74156	88398
Education																
No formal education	20.13	17.69	19.14	16.01	12.51	12.56	13.53	11.24	9.99	9.36	8.96	8.99	7.81	7.16	6.69	6.37
Completed Pre elementary	28.17	27.96	25.57	25.72	25.25	23.22	22.62	23.34	19.01	21.99	19.49	18.52	17.31	15.77	14.70	14.30
Completed Elementary	24.13	24.49	24.28	24.22	24.44	24.58	23.41	23.05	38.03	22.73	23.11	21.53	20.75	20.46	19.81	19.38
Completed Medium school	12.45	14.74	14.78	16.81	17.91	19.99	20.76	22.94	14.85	24.36	25.56	26.65	27.59	29.00	29.29	29.63
Completed High school	8.65	8.50	9.34	10.34	9.88	10.05	11.18	9.16	7.26	10.73	11.12	13.07	13.86	14.55	15.67	16.30
Completed College	6.46	6.63	6.89	6.92	7.35	9.61	8.50	10.28	10.95	10.84	11.76	11.24	12.69	13.06	13.83	14.01
Urbanicity																
Rural	..	23.08	23.46	22.33	22.52	22.51	23.35	22.44	22.28	21.36	21.36	22.02	22.04	21.63	23.03	21.59
Small	..	13.31	13.47	13.51	13.38	13.34	13.43	13.64	13.19	13.84	13.80	13.30	13.46	13.82	13.97	13.67
Medium	..	13.58	13.97	13.36	13.31	13.47	13.47	13.94	14.73	14.53	14.44	14.39	14.75	14.44	14.65	14.87
Metropolitan	..	50.03	49.09	50.80	50.80	50.69	49.76	49.98	49.80	50.27	50.39	50.29	49.75	50.12	48.35	49.87

Supplementary table 3. Purchases by food group per equivalent adult in kilograms, 1989, 2020 ENIGHS

	1989		1992		1994		1996		1998		2000		2002		2004		2006	
Food group	Mean	95% CI	Mean	95% CI	Mean	95% CI	Mean	95% CI	Mean	95% CI	Mean	95% CI	Mean	95% CI	Mean	95% CI	Mean	95% CI
Beef	0.04	0.03, 0.04	0.04	0.03, 0.04	0.04	0.04, 0.04	0.04	0.03, 0.04	0.04	0.04, 0.04	0.05	0.04, 0.05	0.05	0.04, 0.05	0.05	0.04, 0.04	0.05	0.04, 0.04
Dairy	0.20	0.19, 0.21	0.21	0.19, 0.21	0.20	0.19, 0.21	0.19	0.18, 0.19	0.20	0.19, 0.2	0.20	0.19, 0.21	0.21	0.2, 0.22	0.18	0.17, 0.18	0.16	0.15, 0.17
Corn	0.29	0.28, 0.3	0.27	0.25, 0.28	0.26	0.24, 0.26	0.28	0.27, 0.29	0.26	0.24, 0.26	0.27	0.25, 0.27	0.26	0.24, 0.27	0.24	0.22, 0.24	0.22	0.2, 0.22
Legume	0.04	0.03, 0.04	0.04	0.03, 0.04	0.04	0.03, 0.03	0.04	0.03, 0.04	0.04	0.03, 0.03	0.04	0.03, 0.03	0.03	0.03, 0.03	0.03	0.02, 0.03	0.03	0.02, 0.03
Beverages	0.19	0.17, 0.19	0.21	0.19, 0.22	0.23	0.21, 0.24	0.20	0.18, 0.2	0.26	0.25, 0.27	0.29	0.27, 0.31	0.43	0.4, 0.45	0.50	0.47, 0.52	0.63	0.59, 0.66
Sweet or salty	0.04	0.03, 0.03	0.03	0.02, 0.02	0.03	0.02, 0.02	0.02	0.02, 0.02	0.03	0.02, 0.02	0.03	0.02, 0.03	0.03	0.02, 0.03	0.03	0.02, 0.03	0.04	0.02, 0.04
Chicken	0.03	0.02, 0.03	0.04	0.03, 0.04	0.04	0.03, 0.04	0.04	0.03, 0.03	0.04	0.03, 0.03	0.04	0.04, 0.04	0.04	0.04, 0.04	0.05	0.04, 0.04	0.05	0.04, 0.05
Oil	0.03	0.02, 0.02	0.03	0.02, 0.02	0.03	0.02, 0.03	0.03	0.02, 0.03	0.03	0.02, 0.03	0.04	0.03, 0.03	0.03	0.02, 0.02	0.02	0.02, 0.02	0.02	0.02, 0.02
Vegetables	0.13	0.12, 0.13	0.13	0.12, 0.13	0.13	0.12, 0.13	0.14	0.13, 0.14	0.13	0.12, 0.13	0.14	0.13, 0.14	0.15	0.13, 0.15	0.13	0.12, 0.13	0.13	0.12, 0.14
Pork	0.01	0.01, 0.01	0.01	0.01, 0.01	0.01	0.01, 0.01	0.01	0.01, 0.01	0.01	0.01, 0.01	0.01	0.01, 0.01	0.01	0.01, 0.01	0.01	0, 0.01	0.01	0, 0.01
Grain	0.08	0.08, 0.08	0.08	0.07, 0.08	0.08	0.07, 0.08	0.08	0.07, 0.08	0.08	0.07, 0.07	0.08	0.08, 0.08	0.08	0.07, 0.08	0.08	0.07, 0.07	0.08	0.07, 0.07
Seafood	0.01	0, 0.01	0.01	0, 0.01	0.01	0, 0.01	0.01	0, 0.01	0.01	0, 0.01	0.01	0, 0.01	0.01	0, 0.01	0.01	0, 0.01	0.01	0, 0.01
Egg	0.04	0.03, 0.03	0.04	0.03, 0.03	0.04	0.03, 0.03	0.04	0.03, 0.03	0.04	0.03, 0.03	0.04	0.03, 0.04	0.04	0.03, 0.03	0.04	0.03, 0.03	0.04	0.03, 0.03
Others	0.06	0.05, 0.06	0.05	0.04, 0.05	0.06	0.05, 0.05	0.05	0.04, 0.05	0.05	0.04, 0.05	0.05	0.04, 0.05	0.05	0.04, 0.05	0.05	0.04, 0.05	0.05	0.04, 0.06
Fruit	0.08	0.07, 0.08	0.10	0.09, 0.1	0.10	0.09, 0.11	0.09	0.08, 0.09	0.08	0.07, 0.08	0.12	0.1, 0.12	0.10	0.08, 0.1	0.09	0.08, 0.09	0.10	0.09, 0.1
Fastfood	0.00	0, 0	0.01	0, 0.01	0.01	0, 0.01	0.02	0.01, 0.02	0.02	0.01, 0.01	0.02	0.02, 0.02	0.03	0.02, 0.03	0.03	0.02, 0.03	0.05	0.04, 0.04
Seed	0.00	0, 0	0.00	0, 0	0.00	0, 0	0.00	0, 0	0.00	0, 0	0.00	0, 0	0.00	0, 0	0.00	0, 0	0.00	0, 0
Total	1.27	1.24, 1.30	1.28	1.25, 1.31	1.31	1.28, 1.34	1.28	1.25, 1.30	1.30	1.28, 1.33	1.44	1.40, 1.47	1.55	1.51, 1.59	1.53	1.49, 1.57	1.66	1.61, 1.70

Supplementary table 3. Purchases by food group per equivalent adult in kilograms, 1989, 2020 ENIGHS

	2008		2010		2012		2014		2016		2018		2020		Difference 2020-1989	
Food group	Mean	95% CI	Mean	95% CI	Mean	95% CI	Mean	95% CI	Mean	95% CI	Mean	95% CI	Mean	95% CI		95% CI
Beef	0.05	0.04, 0.04	0.05	0.04, 0.05	0.05	0.04, 0.05	0.05	0.04, 0.04	0.04	0.04, 0.04	0.05	0.04, 0.04	0.05	0.04, 0.04	0.01	0.009, 0.01
Dairy	0.15	0.15, 0.15	0.15	0.15, 0.15	0.16	0.15, 0.17	0.16	0.15, 0.16	0.15	0.14, 0.15	0.14	0.13, 0.14	0.13	0.12, 0.13	-0.07	-0.07, -0.06
Corn	0.23	0.22, 0.23	0.23	0.22, 0.24	0.25	0.24, 0.26	0.26	0.24, 0.26	0.24	0.23, 0.24	0.25	0.24, 0.25	0.25	0.25, 0.25	-0.04	-0.04, -0.03
Legume	0.03	0.03, 0.03	0.03	0.02, 0.03	0.03	0.03, 0.03	0.03	0.03, 0.03	0.03	0.03, 0.03	0.03	0.03, 0.03	0.03	0.03, 0.03	-0.01	-0.01, -0.009
Beverages	0.64	0.61, 0.66	0.62	0.59, 0.64	0.67	0.63, 0.71	0.73	0.7, 0.75	0.73	0.7, 0.74	0.72	0.7, 0.73	0.86	0.84, 0.87	0.67	0.65, 0.68
Sweet or salty	0.03	0.02, 0.03	0.03	0.02, 0.03	0.04	0.03, 0.04	0.04	0.03, 0.04	0.04	0.03, 0.04	0.04	0.03, 0.04	0.04	0.03, 0.03	0.0	0.0, 0.0
Chicken	0.05	0.04, 0.05	0.05	0.04, 0.04	0.05	0.04, 0.04	0.05	0.04, 0.05	0.06	0.05, 0.05	0.06	0.05, 0.05	0.06	0.05, 0.05	0.03	0.02, 0.03
Oil	0.02	0.02, 0.02	0.02	0.02, 0.02	0.02	0.02, 0.02	0.03	0.02, 0.02	0.02	0.02, 0.02	0.02	0.02, 0.02	0.02	0.02, 0.02	-0.01	-0.01, -0.009
Vegetables	0.14	0.14, 0.14	0.15	0.14, 0.15	0.16	0.15, 0.16	0.17	0.16, 0.17	0.16	0.16, 0.16	0.16	0.15, 0.16	0.17	0.16, 0.17	0.04	0.03, 0.04
Pork	0.01	0, 0.01	0.01	0, 0.01	0.01	0.01, 0.01	0.01	0.01, 0.01	0.01	0.01, 0.01	0.01	0.01, 0.01	0.02	0.01, 0.01	0.01	0.009, 0.01
Grain	0.08	0.07, 0.08	0.08	0.07, 0.08	0.09	0.08, 0.09	0.09	0.08, 0.08	0.09	0.08, 0.08	0.08	0.08, 0.08	0.08	0.07, 0.08	0.0	0.0, 0.0
Seafood	0.01	0, 0.01	0.01	0, 0.01	0.01	0.01, 0.01	0.01	0.01, 0.01	0.01	0.01, 0.01	0.01	0.01, 0.01	0.01	0.01, 0.01	0.0	0.0, 0.0
Egg	0.04	0.03, 0.04	0.04	0.04, 0.04	0.04	0.03, 0.03	0.04	0.04, 0.04	0.04	0.04, 0.04	0.05	0.04, 0.04	0.05	0.04, 0.05	0.01	0.009, 0.01
Others	0.04	0.03, 0.04	0.04	0.03, 0.04	0.05	0.04, 0.05	0.04	0.04, 0.04	0.05	0.04, 0.04	0.05	0.04, 0.04	0.05	0.04, 0.04	-0.01	-0.01, -0.008
Fruit	0.10	0.09, 0.1	0.09	0.08, 0.09	0.11	0.1, 0.11	0.11	0.1, 0.11	0.11	0.1, 0.11	0.10	0.1, 0.1	0.11	0.11, 0.11	0.03	0.02, 0.03
Fastfood	0.03	0.02, 0.03	0.04	0.03, 0.04	0.07	0.06, 0.07	0.08	0.07, 0.09	0.08	0.07, 0.08	0.10	0.09, 0.09	0.12	0.11, 0.12	0.11	0.11, 0.11
Seed	0.00	0, 0	0.00	0, 0	0.00	0, 0	0.00	0, 0	0.00	0, 0	0.00	0, 0	0.00	0, 0	0.0	0.0, 0.0
Total	1.66	1.64, 1.69	1.66	1.63, 1.69	1.82	1.77, 1.86	1.89	1.85, 1.93	1.87	1.85, 1.90	1.88	1.86, 1.90	2.05	2.03, 2.07	0.78	0.76. 0.80

Supplementary table 4. GHGE by food group per equivalent adult in kilograms of CO2, 1989, 2020 ENIGHS

	1989		1992		1994		1996		1998		2000		2002		2004		2006	
Food group	Mean	95% CI	Mean	95% CI	Mean	95% CI	Mean	95% CI	Mean	95% CI	Mean	95% CI	Mean	95% CI	Mean	95% CI	Mean	95% CI
Beef	0.76	0.72, 0.8	0.79	0.74, 0.84	0.85	0.8, 0.89	0.76	0.72, 0.79	0.81	0.78, 0.84	0.93	0.88, 0.98	0.94	0.89, 0.97	0.89	0.85, 0.91	0.85	0.81, 0.88
Dairy	0.63	0.58, 0.67	0.69	0.64, 0.73	0.79	0.69, 0.87	0.61	0.56, 0.64	0.65	0.6, 0.69	0.66	0.61, 0.7	0.88	0.71, 1.04	0.61	0.56, 0.65	0.57	0.53, 0.6
Corn	0.21	0.2, 0.22	0.18	0.16, 0.19	0.17	0.15, 0.17	0.18	0.16, 0.19	0.16	0.15, 0.17	0.17	0.15, 0.17	0.16	0.15, 0.17	0.14	0.13, 0.15	0.12	0.11, 0.12
Legume	0.15	0.14, 0.16	0.14	0.13, 0.15	0.14	0.13, 0.14	0.15	0.14, 0.15	0.13	0.12, 0.13	0.13	0.12, 0.13	0.12	0.11, 0.12	0.12	0.11, 0.12	0.10	0.09, 0.11
Beverages	0.22	0.2, 0.23	0.21	0.19, 0.22	0.32	0.15, 0.47	0.20	0.18, 0.21	0.23	0.21, 0.24	0.24	0.22, 0.25	0.27	0.25, 0.28	0.29	0.27, 0.3	0.30	0.28, 0.31
Sweet or salty	0.42	0.35, 0.47	0.39	0.25, 0.52	0.45	0.33, 0.56	0.30	0.24, 0.35	0.37	0.3, 0.42	0.45	0.31, 0.58	0.60	0.48, 0.72	0.54	0.47, 0.61	0.49	0.42, 0.55
Chicken	0.18	0.17, 0.19	0.25	0.23, 0.26	0.26	0.25, 0.27	0.23	0.21, 0.23	0.23	0.22, 0.24	0.28	0.26, 0.29	0.31	0.29, 0.32	0.32	0.3, 0.33	0.33	0.32, 0.34
Oil	0.14	0.13, 0.14	0.13	0.12, 0.13	0.15	0.14, 0.15	0.14	0.13, 0.14	0.13	0.12, 0.14	0.15	0.14, 0.16	0.13	0.11, 0.13	0.12	0.11, 0.12	0.11	0.1, 0.11
Vegetables	0.12	0.11, 0.12	0.12	0.11, 0.12	0.12	0.11, 0.12	0.13	0.12, 0.13	0.12	0.11, 0.12	0.13	0.12, 0.13	0.13	0.12, 0.13	0.12	0.11, 0.12	0.13	0.12, 0.13
Pork	0.12	0.11, 0.12	0.12	0.11, 0.13	0.12	0.11, 0.12	0.11	0.1, 0.11	0.12	0.11, 0.13	0.13	0.11, 0.13	0.10	0.09, 0.11	0.09	0.08, 0.09	0.10	0.09, 0.11
Grain	0.07	0.06, 0.07	0.07	0.06, 0.07	0.07	0.06, 0.06	0.06	0.06, 0.06	0.06	0.06, 0.06	0.07	0.06, 0.07	0.07	0.06, 0.07	0.08	0.07, 0.08	0.08	0.07, 0.08
Seafood	0.11	0.08, 0.13	0.08	0.06, 0.08	0.09	0.07, 0.1	0.08	0.06, 0.08	0.07	0.06, 0.07	0.10	0.08, 0.12	0.12	0.1, 0.12	0.07	0.06, 0.08	0.11	0.09, 0.12
Egg	0.06	0.05, 0.06	0.06	0.06, 0.06	0.06	0.06, 0.06	0.06	0.06, 0.06	0.06	0.05, 0.06	0.07	0.06, 0.07	0.06	0.06, 0.06	0.07	0.06, 0.06	0.06	0.06, 0.06
Others	0.05	0.04, 0.05	0.06	0.05, 0.06	0.07	0.06, 0.07	0.06	0.05, 0.06	0.06	0.05, 0.07	0.07	0.06, 0.07	0.06	0.05, 0.06	0.06	0.05, 0.06	0.06	0.05, 0.06
Fruit	0.41	0.39, 0.43	0.48	0.44, 0.51	0.53	0.48, 0.56	0.44	0.41, 0.46	0.40	0.37, 0.42	0.58	0.54, 0.62	0.50	0.46, 0.52	0.49	0.46, 0.52	0.07	0.07, 0.07
Fastfood	0.03	0.02, 0.04	0.08	0.06, 0.09	0.10	0.07, 0.12	0.16	0.13, 0.18	0.12	0.1, 0.13	0.19	0.16, 0.21	0.22	0.18, 0.25	0.24	0.22, 0.26	0.37	0.34, 0.39
Seed	0.00	0, 0	0.00	0, 0	0.00	0, 0	0.00	0, 0	0.00	0, 0	0.00	0, 0	0.00	0, 0	0.00	0, 0	0.00	0, 0
Total	3.70	3.57, 3.82	3.84	3.63, 4.04	4.27	3.96, 4.56	3.66	3.53, 3.79	3.75	3.63, 3.86	4.36	4.17, 4.54	4.67	4.43, 4.90	4.25	4.13, 4.36	3.87	3.76, 3.97

Supplementary table 4. GHGE by food group per equivalent adult in kilograms of CO2, 1989, 2020 ENIGHS

	2008		2010		2012		2014		2016		2018		2020		Difference 2020-1989	
Food group	Mean	95% CI	Mean	95% CI	Mean	95% CI	Mean	95% CI	Mean	95% CI	Mean	95% CI	Mean	95% CI		95% CI
Beef	0.86	0.83, 0.88	0.89	0.86, 0.91	0.87	0.83, 0.91	0.79	0.76, 0.81	0.73	0.71, 0.74	0.76	0.74, 0.77	0.83	0.81, 0.84	0.07	0.05, 0.08
Dairy	0.55	0.52, 0.58	0.57	0.53, 0.6	0.71	0.63, 0.77	0.72	0.65, 0.78	0.67	0.63, 0.7	0.61	0.58, 0.63	0.57	0.54, 0.59	-0.06	-0.08, -0.03
Corn	0.14	0.13, 0.15	0.14	0.13, 0.15	0.17	0.15, 0.18	0.17	0.16, 0.17	0.16	0.15, 0.16	0.17	0.16, 0.16	0.17	0.16, 0.16	-0.04	-0.04, -0.03
Legume	0.11	0.1, 0.11	0.11	0.1, 0.11	0.11	0.1, 0.12	0.12	0.11, 0.12	0.11	0.1, 0.11	0.11	0.1, 0.11	0.11	0.1, 0.1	-0.04	-0.04, -0.03
Beverages	0.30	0.28, 0.3	0.32	0.3, 0.33	0.38	0.35, 0.4	0.36	0.34, 0.37	0.39	0.37, 0.41	0.38	0.36, 0.38	0.42	0.4, 0.42	0.20	0.19, 0.20
Sweet or salty	0.39	0.34, 0.43	0.54	0.46, 0.61	0.82	0.57, 1.07	0.93	0.68, 1.18	0.69	0.61, 0.75	0.77	0.62, 0.91	0.56	0.5, 0.6	0.14	0.09, 0.18
Chicken	0.34	0.33, 0.35	0.33	0.31, 0.33	0.32	0.3, 0.33	0.34	0.32, 0.34	0.37	0.36, 0.37	0.38	0.37, 0.39	0.40	0.38, 0.4	0.22	0.21, 0.22
Oil	0.11	0.1, 0.11	0.11	0.1, 0.11	0.11	0.1, 0.11	0.12	0.11, 0.12	0.12	0.11, 0.11	0.12	0.11, 0.12	0.12	0.11, 0.12	-0.02	-0.02, -0.01
Vegetables	0.13	0.12, 0.13	0.14	0.13, 0.14	0.15	0.13, 0.15	0.16	0.15, 0.16	0.15	0.14, 0.15	0.15	0.15, 0.15	0.16	0.15, 0.16	0.05	0.04, 0.05
Pork	0.10	0.09, 0.1	0.10	0.09, 0.1	0.11	0.1, 0.12	0.11	0.1, 0.11	0.13	0.12, 0.13	0.14	0.13, 0.14	0.17	0.15, 0.17	0.05	0.03, 0.06
Grain	0.08	0.08, 0.08	0.09	0.08, 0.08	0.09	0.08, 0.09	0.09	0.09, 0.09	0.09	0.08, 0.09	0.09	0.08, 0.09	0.09	0.08, 0.08	0.03	0.02, 0.03
Seafood	0.11	0.09, 0.11	0.11	0.1, 0.11	0.12	0.1, 0.13	0.11	0.1, 0.12	0.12	0.11, 0.12	0.12	0.11, 0.12	0.13	0.12, 0.13	0.02	0.01, 0.02
Egg	0.07	0.06, 0.07	0.07	0.07, 0.07	0.07	0.06, 0.06	0.07	0.06, 0.07	0.08	0.07, 0.07	0.08	0.08, 0.08	0.09	0.08, 0.08	0.03	0.02, 0.03
Others	0.06	0.05, 0.06	0.06	0.05, 0.06	0.08	0.07, 0.08	0.06	0.06, 0.06	0.08	0.05, 0.1	0.07	0.06, 0.07	0.08	0.07, 0.08	0.03	0.02, 0.03
Fruit	0.07	0.06, 0.07	0.07	0.06, 0.07	0.08	0.07, 0.08	0.08	0.07, 0.08	0.08	0.07, 0.08	0.08	0.07, 0.07	0.08	0.08, 0.08	-0.33	-0.33, -0.32
Fastfood	0.25	0.23, 0.26	0.30	0.28, 0.32	0.57	0.52, 0.61	0.67	0.61, 0.73	0.66	0.63, 0.68	0.77	0.73, 0.79	0.94	0.91, 0.96	0.91	0.88, 0.93
Seed	0.00	0, 0	0.00	0, 0	0.00	0, 0	0.00	0, 0	0.00	0, 0	0.00	0, 0	0.00	0, 0	0.0	0.0, 0.0
Total	3.68	3.60, 3.74	3.96	3.85, 4.06	4.76	4.47, 5.04	4.90	4.62, 5.18	4.63	4.53, 4.72	4.79	4.63, 4.95	4.89	4.81, 4.96	1.19	1.12, 1.26

Supplementary table 5. Purchases by educational level of the head of household in kilograms per equivalent adult, 1989, 2020 ENIGHS

	1989		1992		1994		1996		1998		2000		2002		2004		2006	
	Mean	95% CI	Mean	95% CI	Mean	95% CI	Mean	95% CI	Mean	95% CI	Mean	95% CI	Mean	95% CI	Mean	95% CI	Mean	95% CI
No formal education	1.13	1.06, 1.18	1.11	1.03, 1.17	1.09	1.02, 1.15	1.11	1.06, 1.15	1.13	1.07, 1.18	1.28	1.19, 1.37	1.34	1.25, 1.42	1.48	1.32, 1.63	1.61	1.47, 1.75
Completed Pre, Elementary	1.18	1.12, 1.22	1.16	1.11, 1.2	1.17	1.12, 1.21	1.14	1.1, 1.18	1.22	1.18, 1.26	1.34	1.28, 1.39	1.47	1.4, 1.54	1.46	1.39, 1.52	1.54	1.47, 1.6
Completed Elementary	1.30	1.25, 1.33	1.30	1.25, 1.34	1.31	1.26, 1.35	1.27	1.22, 1.3	1.29	1.24, 1.32	1.44	1.39, 1.48	1.54	1.47, 1.6	1.50	1.44, 1.54	1.63	1.55, 1.69
Completed Middle School	1.43	1.33, 1.52	1.40	1.33, 1.45	1.46	1.39, 1.51	1.39	1.33, 1.43	1.38	1.33, 1.43	1.45	1.39, 1.5	1.58	1.51, 1.64	1.52	1.46, 1.56	1.65	1.58, 1.72
Completed High School	1.46	1.38, 1.53	1.49	1.4, 1.58	1.63	1.54, 1.71	1.49	1.43, 1.54	1.47	1.39, 1.53	1.56	1.46, 1.65	1.71	1.59, 1.81	1.64	1.57, 1.71	1.64	1.56, 1.72
Completed College	1.48	1.39, 1.56	1.70	1.57, 1.82	1.66	1.57, 1.74	1.62	1.54, 1.7	1.67	1.57, 1.76	1.81	1.65, 1.96	1.91	1.78, 2.02	1.75	1.67, 1.83	2.02	1.85, 2.18
College – no formal education	0.36	0.35, 0.37	0.59	0.58, 0.60	0.56	0.54, 0.58	0.52	0.50, 0.54	0.54	0.51, 0.56	0.52	0.49, 0.55	0.57	0.55, 0.59	0.27	0.24, 0.29	0.40	0.35, 0.44

Supplementary table 5. Purchases by educational level of the head of household in kilograms per equivalent adult, 1989, 2020 ENIGHS

	2008		2010		2012		2014		2016		2018		2020		Difference 2020-1989	
	Mean	95% CI	Mean	95% CI	Mean	95% CI	Mean	95% CI	Mean	95% CI	Mean	95% CI	Mean	95% CI		95% CI
No formal education	1.55	1.48, 1.62	1.56	1.47, 1.65	1.78	1.65, 1.89	1.88	1.77, 1.98	1.99	1.9, 2.07	1.99	1.91, 2.06	2.11	2.03, 2.18	0.98	0.96, 0.99
Completed Pre, Elementary	1.59	1.53, 1.64	1.62	1.55, 1.68	1.78	1.67, 1.88	1.92	1.83, 1.99	1.98	1.92, 2.02	1.97	1.91, 2.01	2.15	2.09, 2.19	0.97	0.95, 0.98
Completed Elementary	1.60	1.55, 1.64	1.59	1.53, 1.64	1.79	1.7, 1.87	1.81	1.74, 1.88	1.81	1.77, 1.84	1.85	1.81, 1.88	2.04	1.99, 2.07	0.74	0.72, 0.75
Completed Middle School	1.62	1.57, 1.66	1.61	1.56, 1.65	1.74	1.66, 1.81	1.82	1.75, 1.87	1.77	1.73, 1.8	1.77	1.73, 1.8	1.92	1.88, 1.95	0.49	0.47, 0.50
Completed High School	1.78	1.71, 1.84	1.77	1.69, 1.84	1.82	1.7, 1.94	1.92	1.83, 1.99	1.85	1.8, 1.9	1.84	1.78, 1.89	1.98	1.93, 2.02	0.52	0.50, 0.53
Completed College	2.01	1.91, 2.1	1.95	1.86, 2.03	2.13	1.96, 2.29	2.14	2.03, 2.24	2.03	1.96, 2.1	2.05	1.98, 2.1	2.32	2.25, 2.38	0.84	0.82, 0.85
College – no formal education	0.46	0.43, 0.48	0.38	0.35, 0.40	0.35	0.30, 0.40	0.26	0.22, 0.29	0.04	0.01, 0.06	0.06	0.04, 0.07	0.20	0.19, 0.21		

Supplementary table 6. Purchases by urbanicity in kilograms per equivalent adult, 1992, 2020 ENIGHS

	1992		1994		1996		1998		2000		2002		2004		2006	
	Mean	95% CI	Mean	95% CI	Mean	95% CI	Mean	95% CI	Mean	95% CI	Mean	95% CI	Mean	95% CI	Mean	95% CI
Metropolitan	1.46	1.41, 1.49	1.48	1.43, 1.52	1.42	1.37, 1.45	1.43	1.4, 1.46	1.56	1.5, 1.6	1.64	1.59, 1.68	1.62	1.58, 1.64	1.74	1.68, 1.79
Medium	1.29	1.19, 1.37	1.38	1.29, 1.47	1.30	1.23, 1.37	1.35	1.28, 1.42	1.47	1.41, 1.53	1.71	1.59, 1.82	1.64	1.55, 1.72	1.70	1.62, 1.77
Small	1.26	1.16, 1.35	1.25	1.15, 1.33	1.19	1.11, 1.26	1.25	1.16, 1.34	1.40	1.27, 1.53	1.52	1.39, 1.64	1.46	1.28, 1.62	1.60	1.41, 1.77
Rural	0.92	0.86, 0.97	0.95	0.9, 0.99	1.00	0.94, 1.05	1.02	0.96, 1.06	1.17	1.11, 1.22	1.27	1.16, 1.37	1.31	1.22, 1.4	1.47	1.38, 1.56
Rural - Metropolitan	-0.54	-0.55, -0.52	-0.53	-0.55, -0.50	-0.42	-0.44, -0.39	-0.42	-0.44, -0.39	-0.38	-0.41, -0.35	-0.36	-0.38, -0.33	-0.30	-0.32, -0.27	-0.27	-0.30, -0.23

Supplementary table 6. Purchases by urbanicity in kilograms per equivalent adult, 1992, 2020 ENIGHS

	2008		2010		2012		2014		2016		2018		2020		Difference 2020-1992	
	Mean	95% CI	Mean	95% CI	Mean	95% CI	Mean	95% CI	Mean	95% CI	Mean	95% CI	Mean	95% CI		95% CI
Metropolitan	1.79	1.75, 1.82	1.76	1.72, 1.79	1.91	1.83, 1.97	1.99	1.93, 2.04	1.93	1.89, 1.96	1.92	1.88, 1.95	2.10	2.06, 2.13	0.64	0.62, 0.65
Medium	1.73	1.66, 1.79	1.69	1.6, 1.78	1.76	1.62, 1.89	1.92	1.83, 1.99	1.89	1.83, 1.94	1.93	1.87, 1.97	2.13	2.07, 2.19	0.84	0.82, 0.85
Small	1.61	1.52, 1.7	1.66	1.53, 1.79	1.78	1.64, 1.9	1.89	1.77, 2.01	1.89	1.82, 1.95	1.88	1.83, 1.93	1.99	1.92, 2.04	0.73	0.71, 0.74
Rural	1.34	1.27, 1.4	1.40	1.31, 1.47	1.68	1.58, 1.76	1.66	1.58, 1.73	1.72	1.68, 1.76	1.75	1.71, 1.79	1.94	1.89, 1.98	1.02	0.99, 1.04
Rural - Metropolitan	-0.45	-0.47, -0.42	-0.37	-0.39, -0.34	-0.23	-0.27, -0.18	-0.33	-0.36, -0.29	-0.20	-0.21, -0.18	-0.17	-0.19, -0.15	-0.15	-0.16, -0.13		

Supplementary table 7. GHGE by educational level of the head of household per equivalent adult in kg of CO_2, 1989, 2020 ENIGHS

	1989		1992		1994		1996		1998		2000		2002		2004		2006	
	Mean	95% CI	Mean	95% CI	Mean	95% CI	Mean	95% CI	Mean	95% CI	Mean	95% CI	Mean	95% CI	Mean	95% CI	Mean	95% CI
No formal education	2.88	2.62, 3.14	2.77	2.56, 2.98	3.75	2.41, 5.09	2.67	2.5, 2.82	2.60	2.43, 2.76	3.13	2.83, 3.43	3.34	2.98, 3.7	3.36	3.08, 3.63	3.44	3.09, 3.78
Completed Pre, Elementary	2.95	2.77, 3.11	3.10	2.92, 3.27	3.27	3.08, 3.45	2.98	2.8, 3.16	3.03	2.88, 3.18	3.56	3.37, 3.75	3.94	3.58, 4.3	3.62	3.42, 3.81	3.47	3.31, 3.63
Completed Elementary	3.80	3.57, 4.03	3.70	3.5, 3.88	4.09	3.83, 4.34	3.59	3.37, 3.79	3.60	3.42, 3.77	4.45	3.91, 4.98	4.71	4.26, 5.15	4.00	3.83, 4.16	3.65	3.49, 3.8
Completed Middle School	4.57	4.25, 4.88	4.43	4.15, 4.71	4.62	4.34, 4.89	4.29	4, 4.57	4.16	3.97, 4.35	4.64	4.35, 4.93	4.83	4.45, 5.21	4.60	4.3, 4.89	4.20	3.96, 4.44
Completed High School	5.15	4.69, 5.6	6.30	4.88, 7.72	5.85	5.34, 6.34	4.79	4.51, 5.05	5.24	4.74, 5.74	5.22	4.66, 5.76	5.55	5.04, 6.05	4.91	4.54, 5.28	4.26	3.91, 4.59
Completed College	5.50	5.03, 5.97	5.94	5.3, 6.58	7.08	6.14, 8.02	5.59	5.17, 6	6.07	5.44, 6.69	6.50	5.82, 7.16	6.68	5.75, 7.61	5.87	5.46, 6.26	4.99	4.57, 5.4
College – no formal education	2.62	2.57, 2.67	3.17	3.11, 3.22	3.33	3.10, 3.55	2.93	2.84, 3.01	3.47	3.34, 3.59	3.36	3.22, 3.49	3.34	3.17, 3.50	2.51	2.39, 2.62	1.56	1.42, 1.69

Supplementary table 7. GHGE by educational level of the head of household per equivalent adult in kg of CO_2, 1989, 2020 ENIGHS

	2008		2010		2012		2014		2016		2018		2020		Difference 2020-1989	
	Mean	95% CI	Mean	95% CI	Mean	95% CI	Mean	95% CI	Mean	95% CI	Mean	95% CI	Mean	95% CI		95% CI
No formal education	3.13	2.9, 3.34	3.61	3.07, 4.13	4.11	3.71, 4.5	4.13	3.6, 4.65	4.36	4.08, 4.63	4.57	4.26, 4.86	4.69	4.47, 4.9	1.81	1.74, 1.87
Completed Pre, Elementary	3.32	3.19, 3.44	3.51	3.29, 3.71	4.62	3.98, 5.26	4.63	4.23, 5.03	4.74	4.45, 5.03	4.69	4.48, 4.9	4.82	4.63, 5	1.87	1.78, 1.95
Completed Elementary	3.48	3.33, 3.61	3.64	3.49, 3.78	4.50	4.18, 4.81	4.44	4.12, 4.75	4.29	4.16, 4.4	4.64	4.33, 4.94	4.72	4.57, 4.86	0.92	0.85, 0.98
Completed Middle School	3.69	3.57, 3.81	3.97	3.8, 4.13	4.74	3.91, 5.56	4.78	4.47, 5.08	4.31	4.18, 4.43	4.64	4.21, 5.06	4.58	4.45, 4.69	0.01	-0.05, 0.07
Completed High School	4.09	3.89, 4.27	4.31	4.07, 4.55	4.80	4.35, 5.24	4.94	4.61, 5.27	4.81	4.55, 5.07	4.85	4.6, 5.09	4.95	4.78, 5.11	-0.2	-0.26, -0.13
Completed College	4.84	4.59, 5.09	5.27	4.9, 5.63	6.01	5.43, 6.57	6.73	4.9, 8.55	5.66	5.28, 6.04	5.50	5.26, 5.72	5.87	5.66, 6.06	0.37	0.31, 0.42
College – no formal education	1.72	1.64, 1.79	1.67	1.50, 1.83	1.9	1.70, 2.10	2.61	2.33, 2.88	1.3	1.16, 1.43	0.93	0.85, 1.00	1.18	1.14, 1.21		

Supplementary table 8 GHGE by urbanicity per equivalent adult in kg of CO2, 1989, 2020 ENIGHS

	1992		1994		1996		1998		2000		2002		2004		2006	
	Mean	95% CI	Mean	95% CI	Mean	95% CI	Mean	95% CI	Mean	95% CI	Mean	95% CI	Mean	95% CI	Mean	95% CI
Metropolitan	4.51	4.29, 4.72	4.84	4.55, 5.12	4.31	4.12, 4.49	4.43	4.24, 4.61	5.06	4.73, 5.37	5.14	4.89, 5.38	4.76	4.63, 4.89	4.23	4.07, 4.37
Medium	4.05	3.32, 4.78	5.40	3.61, 7.19	3.66	3.33, 3.97	3.83	3.56, 4.08	4.45	4.15, 4.75	4.77	4.31, 5.22	4.31	3.94, 4.67	3.87	3.69, 4.04
Small	3.65	2.67, 4.62	3.84	3.31, 4.36	3.30	2.89, 3.69	3.22	2.87, 3.57	4.07	3.67, 4.46	4.53	3.77, 5.28	3.96	3.47, 4.44	3.47	3.2, 3.74
Rural	2.39	2.19, 2.57	2.63	2.45, 2.8	2.43	2.26, 2.59	2.49	2.32, 2.66	2.92	2.72, 3.11	3.70	3.02, 4.37	3.25	3.05, 3.43	3.30	3.09, 3.51
Rural - metropolitan	-2.13	-2.17, -2.08	-2.2	-2.31, -2.08	-1.88	-1.96, -1.79	-1.94	-2.04, -1.83	-2.14	-2.26, -2.01	-1.44	-1.70, -1.17	-1.52	-1.61, -1.42	-0.92	-1.02, -0.81

Supplementary table 8 GHGE by urbanicity per equivalent adult in kg of CO2, 1989, 2020 ENIGHS

	2008		2010		2012		2014		2016		2018		2020		Difference 2020-1992	
	Mean	95% CI	Mean	95% CI	Mean	95% CI	Mean	95% CI	Mean	95% CI	Mean	95% CI	Mean	95% CI		95% CI
Metropolitan	4.00	3.89, 4.1	4.45	4.27, 4.62	5.25	4.71, 5.77	5.50	4.97, 6.02	4.89	4.73, 5.04	4.91	4.77, 5.05	5.14	5.01, 5.25	0.63	0.56, 0.69
Medium	3.76	3.59, 3.93	3.93	3.73, 4.12	4.19	3.87, 4.51	4.49	4.14, 4.84	4.56	4.33, 4.77	4.85	4.46, 5.23	4.95	4.76, 5.13	0.90	0.84, 0.95
Small	3.53	3.34, 3.72	3.73	3.43, 4.01	4.41	4.05, 4.76	4.45	4.11, 4.78	4.70	4.4, 4.99	5.19	4.27, 6.1	4.76	4.57, 4.93	1.11	1.03, 1.18
Rural	2.95	2.82, 3.07	2.99	2.84, 3.12	4.23	3.93, 4.53	4.11	3.79, 4.42	4.02	3.89, 4.14	4.27	4.14, 4.38	4.36	4.26, 4.45	1.97	1.89, 2.04
Rural - metropolitan	-1.05	-1.11, -0.98	-1.46	-1.55, -1.36	-1.01	-1.26, -0.75	-1.39	-1.60, -1.17	-0.86	-0.96, -0.75	-0.65	-0.73, -0.56	-0.77	-0.80, -0.73		

Supplementary table 9. Relative contribution of GHGE by food group in Mexican households, 1989, 2020 ENIGHS

	1989		1992		1994		1996		1998		2000		2002		2004		2006
Food group	Mean	95% CI	Mean	95% CI	Mean	95% CI	Mean	95% CI	Mean	95% CI	Mean	95% CI	Mean	95% CI	Mean	95% CI	Mean
Beef	19.89	19.18, 20.59	20.09	19.26, 20.91	21.20	20.44, 21.95	20.47	19.82, 21.11	21.26	20.54, 21.96	22.42	21.66, 23.16	22.90	22.13, 23.66	22.24	21.64, 22.82	21.14
Dairy	16.87	16.3, 17.42	17.74	17.12, 18.35	17.20	16.47, 17.93	16.40	15.85, 16.94	16.89	16.32, 17.45	15.97	15.36, 16.58	15.86	15.32, 16.39	15.59	15.04, 16.14	14.50
Corn	9.61	9, 10.22	8.63	7.89, 9.35	7.57	7.11, 8.02	8.98	8.42, 9.53	7.93	7.4, 8.46	7.02	6.58, 7.45	6.89	6.32, 7.46	6.07	5.65, 6.49	5.41
Legume	7.03	6.59, 7.46	6.47	6.02, 6.92	5.84	5.46, 6.22	6.86	6.5, 7.2	5.76	5.45, 6.07	5.04	4.7, 5.37	4.80	4.47, 5.12	4.49	4.16, 4.8	3.79
Beverages	6.99	6.66, 7.32	7.22	6.72, 7.71	7.73	7.31, 8.13	6.70	6.36, 7.03	7.95	7.55, 8.34	7.55	7.13, 7.96	7.87	7.5, 8.24	8.81	8.49, 9.11	9.65
Sweet or salty	6.23	5.83, 6.61	5.00	4.53, 5.47	4.99	4.56, 5.4	4.42	4.09, 4.75	5.30	4.92, 5.67	5.13	4.72, 5.53	4.97	4.6, 5.34	5.86	5.48, 6.22	5.74
Chicken	5.87	5.58, 6.15	7.92	7.54, 8.29	8.45	7.92, 8.97	7.61	7.28, 7.92	7.51	7.16, 7.86	8.13	7.79, 8.47	9.30	8.93, 9.65	9.68	9.29, 10.05	10.15
Oil	5.46	5.19, 5.73	5.04	4.74, 5.33	5.08	4.78, 5.36	5.14	4.89, 5.37	4.58	4.35, 4.81	4.59	4.37, 4.8	4.03	3.8, 4.26	3.59	3.43, 3.74	3.22
Vegetables	4.59	4.4, 4.77	4.36	4.14, 4.57	4.33	4.12, 4.54	4.85	4.66, 5.04	4.51	4.32, 4.7	4.23	4.01, 4.43	4.41	4.25, 4.55	4.14	3.97, 4.3	4.09
Pork	4.00	3.77, 4.23	3.74	3.46, 4.02	3.64	3.39, 3.88	3.68	3.45, 3.91	3.95	3.71, 4.17	3.73	3.4, 4.05	3.01	2.76, 3.25	2.72	2.53, 2.9	2.85
Grain	3.05	2.9, 3.19	2.96	2.79, 3.12	2.59	2.46, 2.71	2.75	2.62, 2.88	2.62	2.49, 2.74	2.46	2.28, 2.62	2.44	2.32, 2.56	2.86	2.75, 2.96	2.87
Seafood	2.94	2.65, 3.23	2.27	1.95, 2.58	2.46	2.11, 2.79	2.31	2.09, 2.51	2.14	1.89, 2.38	2.73	2.25, 3.19	3.09	2.83, 3.35	1.96	1.74, 2.17	2.73
Egg	2.53	2.41, 2.65	2.63	2.48, 2.77	2.59	2.31, 2.86	2.71	2.58, 2.82	2.70	2.56, 2.83	2.52	2.38, 2.66	2.44	2.32, 2.55	2.78	2.63, 2.92	2.52
Others	2.21	2.02, 2.39	2.19	1.95, 2.41	2.25	2.07, 2.42	2.24	2.09, 2.39	2.37	2.17, 2.55	2.26	2.04, 2.47	1.99	1.84, 2.13	1.99	1.86, 2.1	1.89
Fruit	1.96	1.83, 2.08	2.07	1.92, 2.2	2.32	2.15, 2.48	2.02	1.89, 2.13	1.83	1.72, 1.94	2.39	2.24, 2.54	1.99	1.84, 2.13	1.99	1.88, 2.09	2.21
Fastfood	0.73	0.61, 0.85	1.63	1.36, 1.9	1.75	1.46, 2.03	2.83	2.52, 3.12	2.66	2.33, 2.98	3.82	3.41, 4.21	3.97	3.55, 4.38	5.22	4.86, 5.57	7.19
Seed	0.03	0.02, 0.04	0.03	0.01, 0.04	0.03	0.02, 0.03	0.03	0.01, 0.04	0.03	0.01, 0.03	0.02	0, 0.02	0.02	0.01, 0.02	0.02	0.01, 0.02	0.04

Supplementary table 9. Relative contribution of GHGE by food group in Mexican households, 1989, 2020 ENIGHS

		2008		2010		2012		2014		2016		2018		2020		Difference 2020-1989	
Food group	95% CI	Mean	95% CI	Mean	95% CI	Mean	95% CI	Mean	95% CI	Mean	95% CI	Mean	95% CI	Mean	95% CI		95% CI
Beef	20.58, 21.7	21.57	21.16, 21.98	21.56	21.14, 21.97	19.48	18.83, 20.11	17.95	17.56, 18.34	16.78	16.55, 17	17.18	16.94, 17.41	17.68	17.46, 17.89	-2.21	-3.0, -1.42
Dairy	14.12, 14.88	14.30	14, 14.6	14.15	13.85, 14.44	13.88	13.37, 14.39	13.92	13.56, 14.28	13.43	13.24, 13.6	12.72	12.54, 12.89	11.21	11.06, 11.36	-5.66	-6.39, -4.93
Corn	5.15, 5.67	5.80	5.52, 6.07	5.69	5.45, 5.91	5.92	5.53, 6.29	5.97	5.72, 6.21	5.54	5.4, 5.67	5.55	5.41, 5.68	5.45	5.33, 5.56	-4.16	-4.73, -3.59
Legume	3.58, 3.99	4.18	4.02, 4.33	4.07	3.9, 4.24	3.74	3.52, 3.96	4.08	3.91, 4.25	3.58	3.48, 3.67	3.48	3.39, 3.56	3.21	3.12, 3.29	-3.82	-4.31, -3.33
Beverages	9.28, 10.01	9.68	9.41, 9.93	9.61	9.33, 9.89	9.76	9.34, 10.17	9.81	9.52, 10.09	10.04	9.88, 10.2	9.61	9.45, 9.76	10.09	9.94, 10.23	3.1	2.58, 3.62
Sweet or salty	5.34, 6.13	5.26	5.01, 5.5	5.47	5.23, 5.69	6.76	6.28, 7.23	6.35	6.01, 6.68	6.12	5.95, 6.28	5.74	5.57, 5.9	4.98	4.85, 5.11	-1.25	-1.72, -0.78
Chicken	9.81, 10.49	10.42	10.16, 10.66	9.53	9.27, 9.78	8.72	8.33, 9.09	9.12	8.86, 9.36	10.15	9.99, 10.3	10.23	10.07, 10.38	10.04	9.9, 10.17	4.17	3.69, 4.65
Oil	3.07, 3.36	3.51	3.38, 3.63	3.53	3.39, 3.66	3.02	2.86, 3.18	3.27	3.14, 3.39	3.11	3.04, 3.17	3.12	3.05, 3.17	2.99	2.93, 3.04	-2.47	-2.91, -2.03
Vegetables	3.9, 4.28	4.40	4.29, 4.5	4.55	4.42, 4.67	4.36	4.16, 4.54	4.65	4.51, 4.77	4.49	4.41, 4.56	4.37	4.31, 4.43	4.47	4.4, 4.53	-0.12	-0.53, 0.29
Pork	2.68, 3.02	2.79	2.63, 2.94	2.82	2.68, 2.95	2.83	2.61, 3.03	2.77	2.61, 2.92	3.28	3.18, 3.37	3.56	3.45, 3.65	3.89	3.79, 3.97	-0.11	-0.5, 0.28
Grain	2.76, 2.98	3.02	2.93, 3.11	3.05	2.94, 3.15	2.96	2.81, 3.09	2.96	2.87, 3.05	2.87	2.81, 2.92	2.75	2.7, 2.8	2.48	2.44, 2.52	-0.57	-0.91, -0.23
Seafood	2.42, 3.04	2.66	2.49, 2.83	2.67	2.52, 2.82	2.65	2.35, 2.93	2.42	2.25, 2.59	2.63	2.51, 2.74	2.44	2.34, 2.54	2.49	2.39, 2.57	-0.45	-0.78, -0.12
Egg	2.38, 2.64	2.87	2.78, 2.96	3.00	2.89, 3.09	2.25	2.13, 2.35	2.54	2.46, 2.61	2.72	2.67, 2.77	2.86	2.8, 2.91	2.83	2.78, 2.88	0.3	-0.01, 0.61
Others	1.76, 2.02	1.84	1.73, 1.94	1.87	1.73, 2	2.07	1.89, 2.24	1.73	1.62, 1.83	1.76	1.69, 1.82	1.82	1.75, 1.87	1.91	1.84, 1.97	-0.3	-0.59, -0.01
Fruit	2.07, 2.34	2.07	2, 2.14	2.01	1.92, 2.09	2.19	2.03, 2.34	2.15	2.05, 2.23	2.19	2.13, 2.24	2.05	1.99, 2.1	2.06	2.01, 2.1	0.1	-0.17, 0.37
Fastfood	6.8, 7.56	5.60	5.31, 5.88	6.40	6.1, 6.68	9.38	8.8, 9.94	10.27	9.85, 10.68	11.27	11, 11.53	12.48	12.2, 12.75	14.17	13.9, 14.43	13.44	13.16, 13.72
Seed	0.02, 0.05	0.03	0.01, 0.03	0.03	0.02, 0.03	0.05	0.02, 0.07	0.04	0.02, 0.04	0.04	0.03, 0.04	0.04	0.03, 0.04	0.04	0.03, 0.04	0.01	-0.02, 0.04

Supplementary table 10. Relative contribution of GHGE by food group among households with no formal education, 1989, 2020 ENIGHS.

	1989		1992		1994		1996		1998		2000		2002		2004		2006
Food group	Mean	95% CI	Mean	95% CI	Mean	95% CI	Mean	95% CI	Mean	95% CI	Mean	95% CI	Mean	95% CI	Mean	95% CI	Mean
Beef	12.06	10.52, 13.59	11.20	9.9, 12.49	12.76	11.35, 14.15	13.70	12.46, 14.93	12.33	10.89, 13.77	13.61	11.98, 15.23	16.42	14.77, 18.07	15.94	14.15, 17.71	14.58
Dairy	13.25	12.02, 14.47	14.03	12.43, 15.63	12.76	11.55, 13.95	13.02	11.85, 14.17	11.61	10.23, 12.99	11.27	10.15, 12.39	11.23	10.11, 12.35	11.88	10.85, 12.9	10.50
Corn	15.81	14.07, 17.55	14.59	12.91, 16.25	13.18	11.92, 14.43	15.26	13.88, 16.63	14.73	13.09, 16.35	13.78	12.16, 15.39	12.56	10.73, 14.37	11.47	10.28, 12.66	10.70
Legume	11.61	10.34, 12.87	11.11	9.83, 12.39	9.91	9.01, 10.79	10.76	9.98, 11.53	10.84	9.86, 11.81	9.67	8.28, 11.06	9.19	8.05, 10.32	8.75	7.66, 9.82	7.56
Beverages	8.12	7.23, 8.99	8.73	7.63, 9.82	9.25	8.2, 10.29	7.02	6.14, 7.9	9.31	8.01, 10.59	8.10	6.9, 9.29	7.79	6.55, 9.03	8.72	7.63, 9.8	9.77
Sweet or salty	5.42	4.68, 6.15	4.54	3.6, 5.48	5.06	3.72, 6.39	3.67	3.11, 4.22	5.38	4.27, 6.48	4.71	3.59, 5.81	4.43	3.43, 5.42	5.00	4.21, 5.78	5.24
Chicken	4.57	4, 5.12	6.62	5.82, 7.4	6.96	6.04, 7.87	6.78	6.09, 7.46	5.68	4.97, 6.39	7.45	6.6, 8.3	9.00	8.06, 9.93	9.42	8.36, 10.48	10.93
Oil	7.05	6.28, 7.81	7.56	6.77, 8.34	6.85	6.08, 7.61	6.30	5.71, 6.89	6.30	5.51, 7.07	6.07	5.48, 6.65	6.12	5.34, 6.89	5.20	4.65, 5.74	4.71
Vegetables	4.71	4.28, 5.14	4.74	4.28, 5.18	4.75	4.3, 5.2	5.38	4.89, 5.86	5.28	4.67, 5.87	4.94	4.34, 5.54	5.04	4.62, 5.44	4.91	4.41, 5.4	4.90
Pork	3.84	3.28, 4.39	3.43	2.86, 3.99	3.58	2.91, 4.24	3.47	2.88, 4.05	3.60	2.87, 4.31	3.29	2.51, 4.06	2.61	2.15, 3.05	2.79	2.14, 3.44	2.79
Grain	3.29	2.89, 3.67	3.55	3.14, 3.95	3.12	2.72, 3.5	3.25	2.85, 3.64	3.27	2.91, 3.62	3.60	2.8, 4.39	2.79	2.51, 3.05	3.49	3.15, 3.83	3.80
Seafood	2.70	2.02, 3.36	1.85	1.36, 2.32	2.78	1.76, 3.79	2.32	1.91, 2.72	2.15	1.45, 2.84	2.75	1.87, 3.62	3.25	2.43, 4.06	1.78	1.35, 2.2	2.92
Egg	2.51	2.21, 2.8	2.71	2.4, 3	3.03	2.39, 3.67	2.96	2.68, 3.23	3.07	2.65, 3.49	3.12	2.7, 3.53	3.30	2.93, 3.66	3.55	3.15, 3.95	2.99
Others	3.03	2.51, 3.55	2.99	2.38, 3.59	2.83	2.31, 3.34	2.98	2.47, 3.49	3.11	2.36, 3.85	2.72	2.18, 3.24	2.08	1.79, 2.36	2.27	1.84, 2.68	2.13
Fruit	1.48	1.22, 1.72	1.47	1.23, 1.7	1.77	1.51, 2.01	1.58	1.3, 1.85	1.45	1.15, 1.73	1.83	1.45, 2.2	1.44	1.24, 1.63	1.52	1.21, 1.82	1.81
Fastfood	0.52	0.32, 0.7	0.84	0.53, 1.15	1.39	0.76, 2.02	1.51	1.07, 1.95	1.85	1.1, 2.58	3.07	1.94, 4.19	2.74	1.78, 3.7	3.26	2.5, 4	4.63
Seed	0.04	0.01, 0.05	0.05	0.01, 0.07	0.03	0.01, 0.03	0.04	0, 0.06	0.04	0.01, 0.07	0.01	0, 0.02	0.02	0, 0.02	0.04	0, 0.07	0.04

Supplementary table 10. Relative contribution of GHGE by food group among households with no formal education, 1989, 2020 ENIGHS.

		2008		2010		2012		2014		2016		2018		2020		Difference 2020-1989	
Food group	95% CI	Mean	95% CI	Mean	95% CI	Mean	95% CI	Mean	95% CI	Mean	95% CI	Mean	95% CI	Mean	95% CI		95% CI
Beef	13.26, 15.89	16.54	15.38, 17.68	16.41	15.18, 17.63	12.64	10.9, 14.36	11.88	10.71, 13.04	11.43	10.77, 12.08	10.76	10.09, 11.41	12.24	11.62, 12.84	0.18	-0.46, 0.82
Dairy	9.61, 11.37	11.57	10.7, 12.43	11.14	10.37, 11.89	11.02	9.64, 12.39	10.20	9.33, 11.05	10.81	10.23, 11.38	10.10	9.58, 10.61	9.26	8.79, 9.72	-3.99	-4.65, -3.33
Corn	9.57, 11.82	9.92	9.12, 10.71	10.81	9.83, 11.77	12.53	11.05, 14.01	13.19	12.06, 14.3	10.83	10.22, 11.44	11.61	10.84, 12.36	10.61	10.06, 11.16	-5.2	-5.91, -4.49
Legume	6.77, 8.34	7.64	7.03, 8.24	7.85	7.18, 8.52	7.42	6.31, 8.53	8.58	7.73, 9.42	6.95	6.45, 7.44	7.13	6.68, 7.56	6.08	5.68, 6.47	-5.53	-6.15, -4.91
Beverages	8.81, 10.72	9.59	8.77, 10.4	8.99	8.23, 9.75	9.11	7.9, 10.31	9.64	8.79, 10.49	10.09	9.54, 10.63	9.62	9.08, 10.15	10.06	9.55, 10.55	1.94	1.39, 2.49
Sweet or salty	4.2, 6.27	4.26	3.69, 4.82	4.59	3.97, 5.2	5.65	4.53, 6.75	4.75	4.02, 5.48	5.53	5.03, 6.03	5.05	4.6, 5.49	4.72	4.31, 5.13	-0.7	-1.14, -0.26
Chicken	9.9, 11.94	10.61	9.84, 11.38	9.83	8.97, 10.69	8.27	7.13, 9.4	8.58	7.82, 9.33	9.95	9.41, 10.48	9.38	8.87, 9.88	9.90	9.43, 10.36	5.33	4.89, 5.77
Oil	4.21, 5.2	4.53	4.11, 4.94	4.97	4.37, 5.57	3.73	3.19, 4.25	3.80	3.41, 4.19	3.60	3.38, 3.81	3.66	3.42, 3.88	3.46	3.23, 3.67	-3.59	-4.08, -3.1
Vegetables	4.38, 5.42	5.00	4.67, 5.31	5.37	5, 5.74	5.17	4.65, 5.68	5.32	4.71, 5.92	4.95	4.72, 5.18	5.02	4.73, 5.29	4.80	4.59, 5.01	0.09	-0.33, 0.51
Pork	2.32, 3.26	2.54	2.17, 2.9	2.53	2.1, 2.95	2.18	1.56, 2.78	2.12	1.72, 2.51	3.01	2.72, 3.29	2.96	2.66, 3.25	3.41	3.13, 3.69	-0.43	-0.81, -0.05
Grain	3.4, 4.2	4.02	3.61, 4.42	3.78	3.44, 4.11	3.92	3.26, 4.57	3.71	3.32, 4.1	3.61	3.36, 3.84	3.31	3.12, 3.48	2.97	2.8, 3.14	-0.32	-0.67, 0.03
Seafood	2.09, 3.73	2.79	2.17, 3.4	2.37	1.98, 2.75	2.98	2.12, 3.83	2.53	2.03, 3.03	2.84	2.48, 3.18	2.68	2.36, 2.99	2.65	2.33, 2.95	-0.05	-0.37, 0.27
Egg	2.7, 3.28	3.63	3.3, 3.95	3.68	3.37, 3.97	2.87	2.44, 3.28	2.94	2.62, 3.25	3.24	3.04, 3.44	3.18	3, 3.34	3.31	3.08, 3.52	0.8	0.49, 1.11
Others	1.77, 2.48	1.94	1.55, 2.33	2.19	1.66, 2.7	2.40	1.72, 3.08	1.63	1.3, 1.95	1.84	1.64, 2.03	1.86	1.57, 2.14	1.65	1.47, 1.81	-1.38	-1.71, -1.05
Fruit	1.42, 2.19	1.73	1.51, 1.95	1.52	1.31, 1.71	1.72	1.32, 2.1	1.89	1.6, 2.17	1.99	1.78, 2.19	1.77	1.56, 1.98	1.75	1.6, 1.9	0.27	0.03, 0.51
Fastfood	3.9, 5.35	3.68	3.1, 4.24	3.95	3.28, 4.61	8.34	6.77, 9.9	9.17	7.91, 10.42	9.27	8.51, 10.02	11.87	11, 12.72	13.08	12.23, 13.92	12.56	12.3, 12.82
Seed	0.01, 0.06	0.02	0, 0.02	0.02	0, 0.03	0.06	0, 0.11	0.05	0.02, 0.07	0.05	0.02, 0.06	0.06	0.04, 0.08	0.03	0.02, 0.04	-0.01	-0.05, 0.03

Supplementary table 11. Relative contribution of GHGE by food group among households with a completed pre, elementary education, 1989, 2020 ENIGHS.

	1989		1992		1994		1996		1998		2000		2002		2004		2006
Food group	Mean	95% CI	Mean	95% CI	Mean	95% CI	Mean	95% CI	Mean	95% CI	Mean	95% CI	Mean	95% CI	Mean	95% CI	Mean
Beef	16.08	14.9, 17.26	17.01	15.72, 18.29	17.76	16.42, 19.09	16.35	15.34, 17.35	17.64	16.37, 18.91	19.03	17.77, 20.28	20.52	19.42, 21.61	19.34	18.05, 20.63	22.25
Dairy	13.63	12.78, 14.48	15.15	13.92, 16.37	14.48	13.45, 15.5	13.44	12.68, 14.18	14.22	13.24, 15.2	13.85	12.84, 14.85	13.88	12.89, 14.85	13.91	12.89, 14.93	14.00
Corn	12.63	11.58, 13.68	11.25	9.98, 12.5	10.25	9.52, 10.98	12.43	11.46, 13.4	11.31	10.26, 12.35	10.03	9.13, 10.91	9.18	8.49, 9.86	8.61	7.71, 9.5	5.73
Legume	9.18	8.38, 9.97	8.27	7.54, 8.98	7.63	7.04, 8.21	9.66	8.98, 10.32	7.99	7.32, 8.66	7.10	6.42, 7.76	6.54	5.93, 7.13	6.24	5.66, 6.8	3.99
Beverages	8.13	7.47, 8.77	7.72	6.79, 8.64	8.59	7.82, 9.35	7.47	6.84, 8.08	8.16	7.41, 8.9	8.17	7.45, 8.89	8.26	7.55, 8.96	9.33	8.28, 10.36	8.94
Sweet or salty	5.40	4.79, 5.99	4.37	3.61, 5.12	4.04	3.51, 4.57	3.54	3.16, 3.92	4.42	3.83, 5	4.27	3.67, 4.85	4.52	3.88, 5.15	4.44	3.9, 4.96	5.54
Chicken	5.27	4.69, 5.84	7.73	7.09, 8.36	8.56	7.89, 9.22	7.22	6.67, 7.76	7.21	6.67, 7.73	7.81	7.21, 8.4	9.36	8.65, 10.06	9.62	9, 10.24	10.57
Oil	6.67	6.14, 7.18	5.85	5.3, 6.38	6.19	5.63, 6.74	6.24	5.79, 6.69	5.34	4.91, 5.75	5.50	5, 5.98	4.91	4.44, 5.36	4.57	4.18, 4.95	3.17
Vegetables	4.70	4.34, 5.05	4.61	4.14, 5.06	4.57	4.26, 4.86	5.03	4.64, 5.4	4.88	4.47, 5.29	4.24	3.9, 4.57	4.81	4.48, 5.13	4.46	4.02, 4.89	4.17
Pork	4.03	3.64, 4.42	4.10	3.56, 4.62	3.99	3.58, 4.39	3.62	3.21, 4.02	3.89	3.48, 4.28	3.76	3.24, 4.27	2.91	2.32, 3.49	2.87	2.53, 3.2	3.19
Grain	3.55	3.29, 3.81	3.48	3.17, 3.79	2.95	2.71, 3.18	3.16	2.96, 3.35	2.97	2.73, 3.21	2.78	2.51, 3.05	2.71	2.5, 2.92	3.23	2.96, 3.48	3.02
Seafood	3.10	2.51, 3.69	2.24	1.72, 2.74	2.18	1.78, 2.57	2.43	2, 2.84	2.14	1.74, 2.53	2.90	2.14, 3.65	2.86	2.31, 3.4	2.18	1.67, 2.67	2.31
Egg	2.65	2.42, 2.88	2.84	2.56, 3.11	3.06	2.51, 3.6	2.96	2.72, 3.19	3.18	2.92, 3.43	3.10	2.76, 3.43	2.77	2.52, 3.01	3.35	3.02, 3.67	2.82
Others	2.69	2.31, 3.06	2.27	1.91, 2.62	2.46	2.14, 2.77	2.44	2.17, 2.71	2.93	2.42, 3.44	1.99	1.7, 2.27	2.13	1.76, 2.48	2.00	1.75, 2.24	1.61
Fruit	1.66	1.46, 1.86	1.81	1.54, 2.07	1.82	1.61, 2.02	1.53	1.38, 1.68	1.45	1.26, 1.63	2.01	1.73, 2.27	1.58	1.42, 1.73	1.58	1.37, 1.79	2.03
Fastfood	0.56	0.34, 0.78	1.27	0.78, 1.76	1.44	1.08, 1.79	2.43	1.98, 2.87	2.23	1.54, 2.9	3.45	2.64, 4.25	3.04	2.48, 3.6	4.25	3.64, 4.86	6.64
Seed	0.05	0.01, 0.08	0.04	0.01, 0.07	0.03	0.01, 0.04	0.06	0.01, 0.09	0.03	0, 0.05	0.02	0, 0.04	0.03	0.01, 0.03	0.02	0, 0.02	0.03

Supplementary table 11. Relative contribution of GHGE by food group among households with a completed pre, elementary education, 1989, 2020 ENIGHS.

		2008		2010		2012		2014		2016		2018		2020		Difference 2020-1989	
Food group	95% CI	Mean	95% CI	Mean	95% CI	Mean	95% CI	Mean	95% CI	Mean	95% CI	Mean	95% CI	Mean	95% CI		95% CI
Beef	21.11, 23.37	18.98	18.18, 19.76	18.55	17.73, 19.36	15.48	14.27, 16.67	14.45	13.63, 15.26	13.56	13.09, 14.03	13.40	12.93, 13.86	13.81	13.37, 14.25	-2.27	-2.99, -1.55
Dairy	13.32, 14.66	12.84	12.11, 13.55	12.63	11.97, 13.27	12.18	11.21, 13.13	12.33	11.61, 13.04	12.10	11.72, 12.47	11.42	11.06, 11.78	10.45	10.13, 10.76	-3.18	-3.85, -2.51
Corn	5.3, 6.14	8.57	7.9, 9.23	8.31	7.79, 8.82	8.58	7.73, 9.42	9.48	8.8, 10.15	8.79	8.4, 9.17	8.63	8.29, 8.96	8.53	8.18, 8.88	-4.1	-4.75, -3.45
Legume	3.66, 4.3	5.77	5.41, 6.12	6.00	5.62, 6.37	6.18	5.55, 6.81	6.07	5.63, 6.5	5.51	5.27, 5.74	5.46	5.23, 5.69	5.02	4.79, 5.24	-4.16	-4.72, -3.6
Beverages	8.42, 9.45	9.21	8.68, 9.72	9.35	8.78, 9.92	8.63	7.86, 9.39	9.12	8.59, 9.65	9.73	9.37, 10.08	9.49	9.14, 9.82	10.20	9.88, 10.51	2.07	1.52, 2.62
Sweet or salty	4.88, 6.19	4.81	4.37, 5.23	4.67	4.22, 5.1	6.19	5.29, 7.09	5.97	5.31, 6.62	5.58	5.24, 5.9	5.32	5, 5.64	4.93	4.64, 5.22	-0.47	-0.91, -0.03
Chicken	10.01, 11.12	10.74	10.17, 11.31	9.78	9.23, 10.32	8.89	7.97, 9.81	9.30	8.7, 9.9	10.45	10.07, 10.82	10.18	9.84, 10.52	10.03	9.7, 10.35	4.76	4.3, 5.22
Oil	2.89, 3.44	4.23	3.94, 4.51	4.38	4.04, 4.71	3.84	3.45, 4.22	3.70	3.43, 3.96	3.48	3.32, 3.63	3.47	3.33, 3.61	3.37	3.24, 3.5	-3.3	-3.78, -2.82
Vegetables	3.89, 4.44	4.86	4.62, 5.09	4.95	4.7, 5.2	4.88	4.4, 5.34	5.02	4.73, 5.31	4.93	4.75, 5.1	4.72	4.57, 4.86	4.63	4.47, 4.78	-0.07	-0.49, 0.35
Pork	2.77, 3.6	2.82	2.48, 3.14	3.00	2.62, 3.37	3.04	2.57, 3.49	2.86	2.53, 3.18	3.29	3.08, 3.49	3.34	3.14, 3.53	3.78	3.57, 3.98	-0.25	-0.64, 0.14
Grain	2.82, 3.21	3.38	3.19, 3.56	3.63	3.36, 3.9	3.59	3.28, 3.9	3.35	3.16, 3.54	3.26	3.13, 3.38	3.05	2.93, 3.16	2.77	2.67, 2.87	-0.78	-1.14, -0.42
Seafood	1.94, 2.67	2.71	2.37, 3.04	2.54	2.19, 2.87	2.95	2.35, 3.54	2.64	2.2, 3.07	2.94	2.64, 3.22	2.67	2.42, 2.91	2.69	2.47, 2.9	-0.41	-0.75, -0.07
Egg	2.37, 3.25	3.26	3.04, 3.47	3.64	3.41, 3.85	2.62	2.37, 2.87	2.89	2.7, 3.07	3.10	2.97, 3.23	3.24	3.11, 3.37	3.12	2.99, 3.24	0.47	0.15, 0.79
Others	1.39, 1.83	1.81	1.59, 2.02	1.81	1.58, 2.04	2.22	1.81, 2.63	1.58	1.32, 1.82	1.59	1.46, 1.71	1.70	1.56, 1.83	1.87	1.72, 2	-0.82	-1.13, -0.51
Fruit	1.73, 2.33	1.61	1.48, 1.73	1.74	1.58, 1.9	2.09	1.81, 2.36	1.80	1.6, 1.98	1.86	1.75, 1.95	1.79	1.65, 1.91	1.77	1.66, 1.88	0.11	-0.14, 0.36
Fastfood	5.7, 7.56	4.36	3.88, 4.82	4.97	4.41, 5.52	8.58	7.44, 9.71	9.39	8.5, 10.27	9.79	9.28, 10.28	12.06	11.44, 12.66	12.97	12.44, 13.49	12.41	12.15, 12.67
Seed	0.01, 0.04	0.05	0.01, 0.09	0.03	0.01, 0.05	0.05	0.01, 0.07	0.04	0.01, 0.07	0.05	0.03, 0.05	0.05	0.04, 0.05	0.04	0.03, 0.04	-0.01	-0.05, 0.03

Supplementary table 12. Relative contribution of GHGE by food group among households with a completed elementary education, 1989, 2020 ENIGHS.

	1989		1992		1994		1996		1998		2000		2002		2004		2006
Food group	Mean	95% CI	Mean	95% CI	Mean	95% CI	Mean	95% CI	Mean	95% CI	Mean	95% CI	Mean	95% CI	Mean	95% CI	Mean
Beef	22.99	21.71, 24.25	21.97	20.62, 23.31	23.99	22.8, 25.18	22.36	21.29, 23.43	22.93	21.82, 24.04	24.69	23.43, 25.93	24.77	23.59, 25.93	23.10	21.99, 24.21	20.84
Dairy	17.74	16.86, 18.61	18.49	17.31, 19.66	18.66	17.09, 20.22	16.53	15.74, 17.31	17.27	16.29, 18.24	15.62	14.78, 16.46	15.85	15.04, 16.66	15.02	14.17, 15.85	13.72
Corn	7.36	6.64, 8.08	7.37	6.53, 8.19	5.69	5.19, 6.19	8.17	7.39, 8.94	6.74	6.18, 7.3	6.09	5.6, 6.58	6.10	5.54, 6.65	5.92	5.23, 6.6	6.06
Legume	5.58	5.07, 6.09	5.58	4.83, 6.33	4.91	4.51, 5.31	6.26	5.82, 6.69	5.00	4.58, 5.41	4.77	4.34, 5.18	4.78	4.34, 5.22	4.66	4.09, 5.22	4.41
Beverages	6.08	5.58, 6.58	6.72	6.02, 7.41	6.66	6.09, 7.21	6.13	5.66, 6.58	7.52	6.87, 8.17	7.05	6.44, 7.66	7.11	6.66, 7.55	8.02	7.41, 8.63	9.63
Sweet or salty	6.08	5.35, 6.8	4.79	4.08, 5.49	4.76	4.1, 5.41	4.23	3.66, 4.79	4.55	3.95, 5.15	4.51	3.72, 5.29	4.26	3.67, 4.84	5.75	4.75, 6.74	5.23
Chicken	6.98	6.45, 7.5	8.88	8.25, 9.5	9.14	8.26, 10.01	8.03	7.57, 8.48	8.45	7.73, 9.16	8.63	7.97, 9.29	9.71	9.02, 10.39	10.26	9.47, 11.04	10.47
Oil	5.17	4.72, 5.61	4.35	3.96, 4.72	4.52	4.14, 4.89	4.92	4.62, 5.21	4.54	4.19, 4.87	4.26	3.9, 4.61	3.90	3.46, 4.32	3.52	3.23, 3.81	3.49
Vegetables	4.71	4.38, 5.04	4.30	4.04, 4.55	4.33	4.04, 4.62	5.00	4.73, 5.26	4.54	4.3, 4.77	4.69	4.29, 5.07	4.49	4.17, 4.79	4.31	3.95, 4.67	4.27
Pork	4.32	3.8, 4.84	4.01	3.51, 4.5	3.99	3.62, 4.35	3.99	3.58, 4.39	4.37	3.98, 4.75	4.07	3.46, 4.68	3.54	3.15, 3.93	2.84	2.5, 3.18	2.98
Grain	2.95	2.76, 3.13	2.87	2.64, 3.09	2.54	2.32, 2.75	2.69	2.53, 2.84	2.68	2.47, 2.89	2.40	2.23, 2.56	2.49	2.18, 2.79	3.02	2.8, 3.22	3.09
Seafood	3.05	2.57, 3.52	2.33	1.8, 2.84	2.13	1.8, 2.45	2.19	1.86, 2.51	2.26	1.81, 2.69	2.53	1.97, 3.08	2.99	2.56, 3.41	2.05	1.54, 2.54	2.73
Egg	2.60	2.41, 2.78	2.67	2.41, 2.92	2.55	2.36, 2.74	2.90	2.68, 3.11	2.75	2.55, 2.95	2.45	2.24, 2.65	2.46	2.3, 2.62	2.92	2.67, 3.15	2.69
Others	1.82	1.5, 2.13	1.90	1.52, 2.27	1.87	1.61, 2.13	1.91	1.68, 2.14	1.98	1.68, 2.28	2.21	1.8, 2.6	1.82	1.61, 2.02	1.81	1.62, 1.99	1.88
Fruit	1.76	1.61, 1.91	2.19	1.97, 2.4	2.34	2.06, 2.6	1.98	1.77, 2.17	1.82	1.6, 2.03	2.33	2.06, 2.59	1.79	1.41, 2.15	1.77	1.62, 1.91	1.86
Fastfood	0.77	0.54, 0.98	1.59	1.23, 1.93	1.88	1.33, 2.41	2.70	2.2, 3.2	2.57	2.11, 3.02	3.69	3.03, 4.34	3.93	3.27, 4.58	5.00	4.33, 5.65	6.60
Seed	0.03	0, 0.05	0.02	0, 0.02	0.03	0.01, 0.04	0.02	0.01, 0.02	0.01	0, 0.01	0.01	0, 0.01	0.02	0, 0.02	0.02	0.01, 0.03	0.03

Supplementary table 12. Relative contribution of GHGE by food group among households with a completed elementary education, 1989, 2020 ENIGHS.

		2008		2010		2012		2014		2016		2018		2020		Difference 2020-1989	
Food group	95% CI	Mean	95% CI	Mean	95% CI	Mean	95% CI	Mean	95% CI	Mean	95% CI	Mean	95% CI	Mean	95% CI		95% CI
Beef	20.07, 21.6	21.49	20.76, 22.22	22.05	21.26, 22.84	20.57	19.25, 21.88	17.55	16.81, 18.29	16.18	15.73, 16.62	16.27	15.83, 16.71	16.79	16.37, 17.2	-6.2	-7.02, -5.38
Dairy	13.15, 14.28	13.83	13.32, 14.33	13.58	13.02, 14.14	13.71	12.77, 14.64	13.77	13.08, 14.45	12.83	12.47, 13.17	12.21	11.89, 12.52	10.90	10.62, 11.18	-6.84	-7.58, -6.1
Corn	5.72, 6.39	5.97	5.52, 6.41	5.76	5.41, 6.1	6.01	5.46, 6.55	6.69	6.28, 7.09	6.27	6.03, 6.5	6.31	6.08, 6.53	6.43	6.2, 6.66	-0.93	-1.44, -0.42
Legume	4.15, 4.66	4.77	4.47, 5.05	4.60	4.26, 4.92	3.91	3.57, 4.25	4.80	4.48, 5.11	4.14	3.96, 4.31	4.14	3.97, 4.29	3.89	3.74, 4.03	-1.69	-2.14, -1.24
Beverages	9.06, 10.19	9.10	8.65, 9.54	9.20	8.6, 9.79	9.44	8.74, 10.13	9.55	9.04, 10.05	9.54	9.24, 9.84	9.30	9.02, 9.58	9.86	9.58, 10.13	3.78	3.29, 4.27
Sweet or salty	4.77, 5.67	5.32	4.71, 5.92	5.14	4.68, 5.58	6.33	5.53, 7.11	5.81	5.25, 6.36	5.99	5.66, 6.31	5.55	5.17, 5.92	4.75	4.49, 4.99	-1.33	-1.8, -0.86
Chicken	9.94, 10.98	10.48	10, 10.94	9.67	9.2, 10.13	9.01	8.28, 9.72	9.48	8.99, 9.96	10.54	10.21, 10.85	10.64	10.32, 10.94	10.06	9.78, 10.32	3.08	2.57, 3.59
Oil	3.25, 3.72	3.82	3.59, 4.03	3.87	3.63, 4.1	2.94	2.65, 3.22	3.53	3.27, 3.77	3.43	3.29, 3.56	3.48	3.34, 3.6	3.29	3.17, 3.41	-1.88	-2.31, -1.45
Vegetables	3.98, 4.56	4.70	4.5, 4.9	4.74	4.49, 4.97	4.35	3.99, 4.7	4.89	4.65, 5.12	4.89	4.74, 5.03	4.66	4.53, 4.78	4.80	4.66, 4.93	0.09	-0.33, 0.51
Pork	2.71, 3.24	2.96	2.66, 3.24	2.82	2.59, 3.04	2.80	2.41, 3.17	2.79	2.5, 3.07	3.51	3.31, 3.71	3.92	3.7, 4.14	4.19	3.99, 4.38	-0.13	-0.53, 0.27
Grain	2.91, 3.27	3.24	3.08, 3.39	3.12	2.97, 3.27	3.01	2.76, 3.25	3.26	3.08, 3.43	3.07	2.97, 3.15	2.93	2.84, 3.02	2.71	2.62, 2.78	-0.24	-0.57, 0.09
Seafood	2.23, 3.23	2.42	2.15, 2.68	2.31	2.06, 2.55	2.27	1.84, 2.69	2.21	1.95, 2.46	2.39	2.19, 2.58	2.22	2.06, 2.36	2.33	2.17, 2.48	-0.72	-1.06, -0.38
Egg	2.53, 2.85	3.15	2.99, 3.31	3.16	3, 3.31	2.48	2.24, 2.7	2.78	2.63, 2.93	3.01	2.9, 3.11	3.12	3.01, 3.22	3.11	2.99, 3.21	0.51	0.19, 0.83
Others	1.65, 2.11	1.80	1.58, 2.01	1.93	1.65, 2.2	1.97	1.64, 2.3	1.65	1.39, 1.9	1.79	1.63, 1.94	1.78	1.65, 1.9	1.79	1.67, 1.89	-0.03	-0.29, 0.23
Fruit	1.71, 2	2.02	1.87, 2.16	1.84	1.67, 1.99	1.89	1.63, 2.13	1.99	1.8, 2.17	2.03	1.92, 2.13	1.74	1.64, 1.82	1.88	1.78, 1.97	0.12	-0.14, 0.38
Fastfood	6.04, 7.16	4.91	4.48, 5.33	6.19	5.61, 6.76	9.29	8.22, 10.35	9.22	8.51, 9.91	10.36	9.91, 10.81	11.70	11.2, 12.18	13.19	12.69, 13.67	12.42	12.14, 12.7
Seed	0.01, 0.04	0.02	0.01, 0.02	0.03	0.01, 0.03	0.03	0.01, 0.05	0.03	0.01, 0.04	0.04	0.03, 0.04	0.04	0.03, 0.05	0.04	0.02, 0.04	0.01	-0.02, 0.04

Supplementary table 13. Relative contribution of GHGE by food group among households with a completed middle school, 1989, 2020 ENIGHS.

	1989		1992		1994		1996		1998		2000		2002		2004		2006
Food group	Mean	95% CI	Mean	95% CI	Mean	95% CI	Mean	95% CI	Mean	95% CI	Mean	95% CI	Mean	95% CI	Mean	95% CI	Mean
Beef	26.13	24.7, 27.55	25.27	23.74, 26.78	25.40	23.99, 26.79	23.83	22.76, 24.9	25.11	23.88, 26.34	24.50	22.89, 26.09	25.41	24.12, 26.69	24.69	23.72, 25.65	22.49
Dairy	20.09	18.67, 21.5	20.99	19.55, 22.43	20.42	18.96, 21.86	18.89	17.82, 19.94	19.16	18, 20.32	18.44	17, 19.87	17.84	16.85, 18.81	17.45	16.5, 18.39	16.10
Corn	4.89	4.24, 5.53	4.44	3.88, 4.98	4.38	3.91, 4.83	4.72	4.34, 5.09	4.50	4.07, 4.92	4.09	3.67, 4.5	4.08	3.76, 4.38	3.96	3.65, 4.26	3.47
Legume	3.28	2.7, 3.85	3.48	3, 3.95	2.96	2.58, 3.32	4.29	3.86, 4.7	3.55	3.18, 3.91	3.05	2.62, 3.46	3.03	2.75, 3.29	2.89	2.56, 3.21	2.49
Beverages	5.81	5.08, 6.53	6.18	5.41, 6.93	7.10	6.07, 8.12	6.18	5.56, 6.79	7.53	6.7, 8.35	7.32	6.55, 8.08	7.73	7.09, 8.37	8.62	8.05, 9.18	9.49
Sweet or salty	7.84	6.52, 9.15	5.93	5.08, 6.78	5.90	4.88, 6.91	5.88	5, 6.75	5.82	4.97, 6.67	6.65	5.42, 7.88	5.48	4.42, 6.53	6.51	5.42, 7.59	6.41
Chicken	6.71	6.06, 7.36	8.39	7.67, 9.09	9.71	8.76, 10.65	8.30	7.68, 8.91	7.79	7.13, 8.44	8.95	8.05, 9.83	10.12	9.46, 10.77	9.88	9.3, 10.44	9.83
Oil	3.69	3.27, 4.09	3.46	3.06, 3.85	3.51	3.06, 3.94	4.23	3.84, 4.61	3.70	3.36, 4.03	3.63	3.26, 4	3.30	2.87, 3.71	2.94	2.69, 3.18	2.89
Vegetables	4.50	4.13, 4.85	4.19	3.81, 4.55	4.04	3.66, 4.41	4.53	4.21, 4.83	4.24	3.98, 4.5	3.95	3.57, 4.32	4.13	3.81, 4.45	3.89	3.61, 4.16	3.84
Pork	4.34	3.7, 4.98	3.76	3.21, 4.3	3.40	2.91, 3.88	4.19	3.6, 4.77	4.52	4.02, 5.01	3.97	3.36, 4.57	3.14	2.69, 3.58	2.66	2.37, 2.95	2.84
Grain	2.70	2.47, 2.91	2.38	2.16, 2.59	2.20	1.96, 2.43	2.46	2.26, 2.64	2.10	1.95, 2.23	1.97	1.81, 2.11	2.34	2.1, 2.57	2.55	2.4, 2.69	2.32
Seafood	2.96	2.39, 3.51	2.43	1.83, 3.03	2.50	1.97, 3.02	2.08	1.72, 2.43	2.11	1.76, 2.46	2.25	1.89, 2.61	3.03	2.63, 3.42	1.56	1.29, 1.82	2.62
Egg	2.37	2.12, 2.61	2.49	2.14, 2.83	2.16	1.97, 2.35	2.52	2.33, 2.7	2.32	2.14, 2.49	2.23	2, 2.45	2.31	2.13, 2.48	2.42	2.23, 2.6	2.17
Others	1.46	1.22, 1.69	1.97	1.17, 2.76	1.89	1.46, 2.31	1.72	1.46, 1.98	1.98	1.68, 2.26	2.41	1.77, 3.04	1.94	1.64, 2.23	2.00	1.76, 2.22	1.92
Fruit	2.49	2.19, 2.78	2.30	2.02, 2.56	2.79	2.34, 3.23	2.25	2.02, 2.47	2.07	1.79, 2.33	2.45	2.15, 2.74	1.97	1.75, 2.17	2.13	1.87, 2.38	2.29
Fastfood	0.72	0.47, 0.95	2.34	1.66, 3	1.62	1.09, 2.14	3.92	3.18, 4.66	3.47	2.81, 4.13	4.14	3.45, 4.83	4.15	3.53, 4.77	5.85	5.22, 6.47	8.83
Seed	0.03	0, 0.05	0.01	0, 0.01	0.03	0, 0.05	0.01	0, 0.01	0.01	0, 0.02	0.01	0, 0.01	0.01	0, 0.01	0.01	0, 0.01	0.03

Supplementary table 13. Relative contribution of GHGE by food group among households with a completed middle school, 1989, 2020 ENIGHS.

		2008		2010		2012		2014		2016		2018		2020		Difference 2020-1989	
Food group	95% CI	Mean	95% CI	Mean	95% CI	Mean	95% CI	Mean	95% CI	Mean	95% CI	Mean	95% CI	Mean	95% CI		95% CI
Beef	21.29, 23.67	23.59	22.83, 24.34	23.31	22.59, 24.01	21.02	19.82, 22.2	19.55	18.85, 20.24	17.98	17.59, 18.35	18.42	18.02, 18.81	18.88	18.51, 19.24	-7.25	-8.11, -6.39
Dairy	15.27, 16.91	15.09	14.53, 15.63	14.81	14.24, 15.37	14.40	13.59, 15.2	14.49	13.92, 15.05	13.97	13.67, 14.26	13.10	12.82, 13.38	11.51	11.26, 11.74	-8.58	-9.36, -7.8
Corn	3.16, 3.76	4.30	4.05, 4.54	4.39	4.1, 4.66	4.48	4.11, 4.84	4.37	4.16, 4.57	4.60	4.46, 4.73	4.68	4.52, 4.83	4.73	4.59, 4.85	-0.16	-0.59, 0.27
Legume	2.08, 2.89	3.02	2.83, 3.21	2.93	2.73, 3.12	2.81	2.55, 3.06	3.27	3.07, 3.45	3.05	2.95, 3.15	2.99	2.88, 3.08	2.84	2.74, 2.93	-0.44	-0.79, -0.09
Beverages	8.73, 10.23	9.57	9.11, 10.02	9.43	9, 9.85	9.58	8.87, 10.29	9.41	8.98, 9.83	9.77	9.51, 10.03	9.34	9.1, 9.57	9.83	9.61, 10.05	4.02	3.54, 4.5
Sweet or salty	5.17, 7.63	5.21	4.81, 5.6	5.62	5.16, 6.06	6.93	5.99, 7.85	6.83	6.21, 7.43	6.12	5.81, 6.42	5.61	5.34, 5.87	4.73	4.52, 4.94	-3.11	-3.63, -2.59
Chicken	9.08, 10.57	10.65	10.2, 11.08	9.87	9.43, 10.31	9.31	8.66, 9.95	9.29	8.87, 9.69	10.41	10.14, 10.66	10.72	10.45, 10.97	10.46	10.22, 10.68	3.75	3.24, 4.26
Oil	2.59, 3.19	3.15	2.95, 3.34	3.10	2.92, 3.26	2.94	2.67, 3.2	3.33	3.09, 3.57	3.16	3.06, 3.25	3.22	3.11, 3.32	3.15	3.04, 3.26	-0.54	-0.91, -0.17
Vegetables	3.55, 4.12	4.13	3.96, 4.29	4.41	4.21, 4.6	4.35	3.98, 4.71	4.55	4.3, 4.8	4.42	4.3, 4.53	4.29	4.18, 4.39	4.44	4.34, 4.54	-0.06	-0.47, 0.35
Pork	2.44, 3.22	2.97	2.72, 3.22	2.91	2.64, 3.16	3.12	2.69, 3.54	2.90	2.65, 3.14	3.48	3.31, 3.63	3.90	3.73, 4.07	4.25	4.08, 4.41	-0.09	-0.49, 0.31
Grain	2.17, 2.46	2.71	2.59, 2.82	2.81	2.68, 2.93	2.81	2.6, 3	2.93	2.77, 3.07	2.83	2.76, 2.89	2.74	2.64, 2.82	2.46	2.4, 2.52	-0.24	-0.56, 0.08
Seafood	2.22, 3.01	2.50	2.24, 2.75	2.36	2.15, 2.56	2.20	1.88, 2.51	2.17	1.94, 2.38	2.17	2.03, 2.3	2.09	1.97, 2.21	2.12	2, 2.23	-0.84	-1.17, -0.51
Egg	1.96, 2.37	2.71	2.56, 2.85	2.93	2.75, 3.1	2.07	1.91, 2.23	2.57	2.44, 2.68	2.76	2.68, 2.83	2.93	2.83, 3.02	2.93	2.85, 3	0.56	0.26, 0.86
Others	1.6, 2.22	1.93	1.66, 2.2	1.92	1.65, 2.18	1.90	1.61, 2.17	1.82	1.62, 2.02	1.80	1.69, 1.9	1.83	1.72, 1.93	1.96	1.84, 2.06	0.5	0.26, 0.74
Fruit	2.03, 2.55	2.02	1.89, 2.14	1.95	1.8, 2.09	2.22	1.86, 2.57	1.97	1.82, 2.1	1.99	1.89, 2.09	1.85	1.76, 1.94	1.88	1.8, 1.95	-0.61	-0.91, -0.31
Fastfood	7.85, 9.79	6.42	5.88, 6.95	7.24	6.65, 7.82	9.80	8.79, 10.8	10.54	9.83, 11.24	11.46	11.03, 11.87	12.25	11.84, 12.65	13.80	13.39, 14.19	13.08	12.8, 13.36
Seed	0.01, 0.04	0.02	0, 0.03	0.02	0.01, 0.02	0.07	, 0.02, 0.16	0.02	0.01, 0.03	0.04	0.02, 0.04	0.03	0.02, 0.03	0.04	0.02, 0.04	0.01	-0.02, 0.04

Supplementary table 14. Relative contribution of GHGE by food group among households with a completed high school, 1989, 2020 ENIGHS.

	1989		1992		1994		1996		1998		2000		2002		2004		2006
Food group	Mean	95% CI	Mean	95% CI	Mean	95% CI	Mean	95% CI	Mean	95% CI	Mean	95% CI	Mean	95% CI	Mean	95% CI	Mean
Beef	27.15	25.02, 29.27	26.66	24.47, 28.84	28.53	26.38, 30.67	26.64	25.02, 28.25	26.72	25.1, 28.34	26.97	24.75, 29.17	25.19	23.6, 26.77	25.12	23.68, 26.55	22.66
Dairy	22.93	20.71, 25.14	22.72	20.57, 24.87	21.24	19.37, 23.1	21.06	19.56, 22.56	21.47	19.87, 23.06	18.85	17.21, 20.48	19.24	17.75, 20.72	17.63	16.56, 18.68	17.05
Corn	3.62	3.08, 4.16	3.47	2.68, 4.24	2.69	2.31, 3.07	3.79	3.24, 4.33	3.29	2.85, 3.73	3.66	2.72, 4.58	3.33	2.69, 3.96	3.23	2.79, 3.65	2.94
Legume	2.74	2.27, 3.2	2.32	1.88, 2.76	2.61	1.56, 3.65	2.81	2.37, 3.24	2.29	1.98, 2.59	2.34	1.87, 2.8	1.88	1.59, 2.16	2.02	1.75, 2.28	1.53
Beverages	5.75	4.85, 6.64	5.91	4.81, 7.01	6.68	5.63, 7.72	6.19	5.43, 6.93	7.93	6.87, 8.98	7.54	6.6, 8.48	8.55	7.6, 9.5	9.09	8.43, 9.75	9.97
Sweet or salty	7.51	6.01, 8.99	7.00	5.13, 8.87	5.29	4.41, 6.17	4.67	3.7, 5.63	7.18	5.66, 8.7	5.20	4.01, 6.37	5.08	4.2, 5.95	7.00	6.18, 7.82	6.87
Chicken	6.19	5.42, 6.95	7.58	6.53, 8.62	8.01	7.12, 8.9	8.00	7.13, 8.86	7.34	6.52, 8.16	7.71	6.86, 8.55	8.98	8.12, 9.83	9.36	8.63, 10.08	9.81
Oil	2.93	2.47, 3.38	3.37	2.79, 3.95	3.48	2.82, 4.13	3.56	3.13, 3.97	2.88	2.53, 3.22	3.86	3.42, 4.28	2.69	2.22, 3.15	2.39	2.14, 2.64	2.20
Vegetables	4.25	3.81, 4.68	3.62	3.21, 4.03	4.03	3.31, 4.74	4.33	3.92, 4.74	3.57	3.22, 3.9	3.39	2.96, 3.82	3.79	3.35, 4.21	3.61	3.34, 3.88	3.40
Pork	3.72	3.07, 4.37	2.89	2.37, 3.41	2.96	2.31, 3.6	3.19	2.72, 3.64	3.34	2.8, 3.87	3.95	2.54, 5.34	3.10	2.44, 3.75	2.45	2.09, 2.79	2.41
Grain	2.26	1.94, 2.58	2.11	1.79, 2.42	1.81	1.67, 1.94	2.13	1.95, 2.3	2.09	1.85, 2.33	1.93	1.75, 2.1	2.13	1.9, 2.34	2.21	2.04, 2.36	2.33
Seafood	3.19	2.52, 3.86	2.67	1.98, 3.35	2.71	2.13, 3.28	2.35	1.82, 2.87	1.90	1.45, 2.33	2.89	2.12, 3.65	3.46	2.89, 4.02	1.98	1.44, 2.51	2.64
Egg	2.49	2.01, 2.97	2.39	2.04, 2.73	1.87	1.65, 2.08	2.15	1.92, 2.37	2.17	1.82, 2.51	2.07	1.78, 2.35	1.67	1.5, 1.84	2.23	2.03, 2.43	2.17
Others	1.35	1.05, 1.64	1.89	1.34, 2.43	2.19	1.77, 2.6	2.36	1.87, 2.85	1.94	1.47, 2.39	2.10	1.51, 2.68	2.04	1.51, 2.55	1.87	1.54, 2.18	2.31
Fruit	2.71	2.21, 3.19	2.41	2.04, 2.76	3.22	2.75, 3.69	2.72	2.37, 3.07	2.47	2.01, 2.92	3.01	2.57, 3.44	2.68	2.27, 3.07	2.46	2.09, 2.82	2.77
Fastfood	1.18	0.65, 1.71	2.94	1.85, 4.02	2.64	1.64, 3.64	4.02	3.09, 4.94	3.42	2.45, 4.37	4.51	3.31, 5.7	6.18	4.84, 7.52	7.34	6.23, 8.43	8.92
Seed	0.01	0, 0.02	0.04	, 0.02, 0.1	0.02	0, 0.02	0.02	0.01, 0.03	0.01	0, 0.01	0.02	0, 0.03	0.01	0, 0.01	0.01	0, 0.02	0.02

Supplementary table 14. Relative contribution of GHGE by food group among households with a completed high school, 1989, 2020 ENIGHS.

		2008		2010		2012		2014		2016		2018		2020		Difference 2020-1989	
Food group	95% CI	Mean	95% CI	Mean	95% CI	Mean	95% CI	Mean	95% CI	Mean	95% CI	Mean	95% CI	Mean	95% CI		95% CI
Beef	21, 24.32	24.33	23.17, 25.48	23.86	22.8, 24.9	21.83	20.06, 23.6	20.26	19.28, 21.23	18.68	18.11, 19.24	19.19	18.65, 19.73	19.79	19.26, 20.31	-7.36	-8.23, -6.49
Dairy	15.49, 18.6	15.69	14.98, 16.39	15.96	15.13, 16.79	14.87	13.14, 16.58	15.66	14.54, 16.77	14.56	14.09, 15.02	13.90	13.45, 14.34	11.97	11.59, 12.35	-10.96	-11.77, -10.15
Corn	2.55, 3.32	3.16	2.91, 3.41	3.24	2.86, 3.61	3.64	3.01, 4.26	3.17	2.95, 3.39	3.21	3.07, 3.34	3.46	3.28, 3.62	3.48	3.35, 3.61	-0.14	-0.51, 0.23
Legume	1.29, 1.77	2.12	1.92, 2.3	2.21	2, 2.42	1.55	1.31, 1.79	2.13	1.89, 2.35	2.06	1.94, 2.18	2.07	1.95, 2.19	2.00	1.89, 2.11	-0.74	-1.06, -0.42
Beverages	9.13, 10.8	11.14	10.29, 11.99	10.26	9.61, 10.9	11.04	9.86, 12.21	10.29	9.56, 11	10.61	10.19, 11.02	9.91	9.54, 10.27	10.19	9.86, 10.5	4.44	3.96, 4.92
Sweet or salty	5.67, 8.07	5.81	5.07, 6.54	6.40	5.66, 7.13	7.84	6.09, 9.57	6.88	5.97, 7.78	6.26	5.81, 6.7	6.19	5.74, 6.63	5.22	4.89, 5.53	-2.29	-2.8, -1.78
Chicken	8.9, 10.71	10.10	9.49, 10.7	9.13	8.58, 9.66	8.30	7.3, 9.29	9.13	8.54, 9.71	10.09	9.71, 10.46	10.22	9.84, 10.59	10.16	9.86, 10.46	3.97	3.48, 4.46
Oil	1.9, 2.49	2.56	2.34, 2.77	2.47	2.25, 2.67	2.64	2.24, 3.03	2.73	2.49, 2.96	2.85	2.69, 3	2.65	2.51, 2.78	2.58	2.46, 2.68	-0.35	-0.68, -0.02
Vegetables	3.03, 3.77	3.76	3.49, 4.01	3.92	3.68, 4.16	3.40	2.99, 3.81	4.30	3.92, 4.67	3.92	3.75, 4.08	3.96	3.8, 4.1	3.90	3.78, 4.02	-0.35	-0.75, 0.05
Pork	1.91, 2.89	2.53	2.23, 2.82	2.92	2.57, 3.26	2.99	2.42, 3.56	3.11	2.71, 3.51	3.30	3.04, 3.55	3.62	3.35, 3.87	3.87	3.65, 4.07	0.15	-0.23, 0.53
Grain	2.04, 2.62	2.46	2.3, 2.61	2.57	2.41, 2.73	2.25	2.02, 2.48	2.41	2.25, 2.56	2.50	2.37, 2.61	2.49	2.36, 2.62	2.19	2.11, 2.27	-0.07	-0.36, 0.22
Seafood	2.08, 3.19	2.85	2.53, 3.16	3.14	2.74, 3.54	2.33	1.82, 2.82	2.39	2.09, 2.68	2.77	2.55, 2.98	2.43	2.24, 2.61	2.47	2.3, 2.63	-0.72	-1.06, -0.38
Egg	1.84, 2.48	2.36	2.07, 2.65	2.32	2.14, 2.49	1.97	1.65, 2.28	2.24	2.04, 2.43	2.29	2.18, 2.39	2.57	2.42, 2.7	2.54	2.43, 2.64	0.05	-0.26, 0.36
Others	1.73, 2.88	1.72	1.41, 2.03	1.57	1.31, 1.83	2.16	1.64, 2.68	1.78	1.51, 2.04	1.69	1.54, 1.83	1.80	1.65, 1.95	2.00	1.84, 2.15	0.65	0.42, 0.88
Fruit	2.33, 3.19	2.20	1.98, 2.42	2.15	1.94, 2.35	2.62	2.18, 3.06	2.34	2.08, 2.58	2.28	2.13, 2.43	2.19	2.04, 2.32	2.15	2.03, 2.26	-0.56	-0.88, -0.24
Fastfood	7.56, 10.28	7.18	6, 8.34	7.85	6.99, 8.7	10.54	8.78, 12.29	11.15	10.16, 12.14	12.88	12.23, 13.53	13.32	12.71, 13.92	15.45	14.88, 16.02	14.27	13.96, 14.58
Seed	0, 0.02	0.02	0, 0.04	0.03	0.01, 0.03	0.02	0, 0.02	0.03	0.01, 0.04	0.03	0.02, 0.04	0.04	0.02, 0.05	0.03	0.02, 0.03	0.02	0, 0.04

Supplementary table 15. Relative contribution of GHGE by food group among households with a completed college education, 1989, 2020 ENIGHS.

	1989		1992		1994		1996		1998		2000		2002		2004		2006
Food group	Mean	95% CI	Mean	95% CI	Mean	95% CI	Mean	95% CI	Mean	95% CI	Mean	95% CI	Mean	95% CI	Mean	95% CI	Mean
Beef	27.56	25.07, 30.04	29.94	27.22, 32.66	28.61	26, 31.2	27.46	25.6, 29.31	28.30	25.78, 30.81	28.16	25.35, 30.96	26.22	22.71, 29.73	25.70	24.39, 26.99	23.38
Dairy	24.63	22.17, 27.08	22.15	20.26, 24.03	22.14	19.76, 24.5	21.77	20.01, 23.52	22.53	20.36, 24.69	19.47	17.58, 21.34	19.45	17.36, 21.53	18.81	17.8, 19.8	17.88
Corn	2.65	2.19, 3.1	2.25	1.73, 2.76	2.10	1.68, 2.51	2.57	2.04, 3.08	2.52	2.1, 2.93	2.30	1.75, 2.85	4.94	0.22, 9.64	2.01	1.81, 2.2	2.11
Legume	1.72	1.32, 2.11	1.82	1.45, 2.17	1.72	1.32, 2.11	1.78	1.4, 2.15	1.60	1.34, 1.84	1.49	1.17, 1.8	1.28	1.01, 1.54	1.25	1.08, 1.4	1.16
Beverages	5.86	4.7, 7.02	6.91	5.13, 8.68	6.80	5.78, 7.82	7.17	5.86, 8.48	7.94	6.65, 9.21	7.49	5.86, 9.11	8.81	7.24, 10.37	9.65	9.01, 10.28	10.84
Sweet or salty	8.07	6.69, 9.43	5.02	3.87, 6.17	6.70	5.13, 8.27	6.22	4.77, 7.65	6.41	4.97, 7.84	5.95	4.31, 7.58	6.95	5.67, 8.22	7.81	6.89, 8.72	6.67
Chicken	6.30	5.33, 7.25	8.09	6.97, 9.2	7.59	6.41, 8.76	7.21	6.23, 8.17	8.24	7.18, 9.29	7.36	6.6, 8.1	7.41	6.3, 8.52	8.61	7.94, 9.27	8.29
Oil	3.17	2.57, 3.76	3.15	2.57, 3.72	3.49	3.11, 3.87	3.63	3.22, 4.04	3.07	2.68, 3.44	3.91	3.27, 4.53	2.14	1.74, 2.54	2.28	2.04, 2.51	2.11
Vegetables	3.95	3.35, 4.54	3.92	3.24, 4.58	3.32	2.96, 3.68	4.07	3.58, 4.54	3.66	3.24, 4.07	3.64	3.14, 4.12	3.59	3.02, 4.15	3.18	2.97, 3.38	3.42
Pork	2.91	2.16, 3.66	3.14	2.23, 4.05	2.74	2.07, 3.4	2.88	2.3, 3.45	2.74	2.2, 3.27	2.87	2.17, 3.56	2.03	1.37, 2.69	2.37	1.77, 2.96	2.18
Grain	2.28	1.73, 2.81	1.90	1.61, 2.18	1.83	1.65, 2	1.99	1.8, 2.16	1.73	1.55, 1.91	1.86	1.67, 2.04	1.63	1.39, 1.85	2.26	2.07, 2.44	2.10
Seafood	2.23	1.77, 2.68	2.45	1.83, 3.05	3.27	2.43, 4.11	2.74	2.17, 3.3	2.17	1.68, 2.66	3.34	2.52, 4.16	3.52	2.85, 4.17	2.35	1.96, 2.73	3.51
Egg	2.17	1.73, 2.6	2.05	1.67, 2.43	1.61	1.37, 1.85	1.80	1.61, 1.97	1.42	1.23, 1.59	1.56	1.32, 1.78	1.43	1.14, 1.7	1.60	1.45, 1.74	1.67
Others	1.57	1.09, 2.05	1.64	1.24, 2.03	2.08	1.63, 2.53	2.03	1.45, 2.59	2.16	1.66, 2.64	2.46	1.69, 3.22	1.92	1.32, 2.51	2.13	1.75, 2.51	1.87
Fruit	3.44	2.77, 4.1	3.34	2.57, 4.1	3.43	2.97, 3.88	3.35	2.79, 3.9	2.48	2.18, 2.78	3.41	2.88, 3.93	3.66	2.95, 4.35	3.18	2.89, 3.46	3.59
Fastfood	1.46	0.76, 2.16	2.20	1.37, 3.01	2.53	1.42, 3.64	3.33	2.43, 4.23	3.00	2.09, 3.91	4.71	3.38, 6.02	5.02	3.43, 6.59	6.77	5.87, 7.66	9.12
Seed	0.01	0, 0.01	0.02	0, 0.03	0.04	0.01, 0.05	0.02	0, 0.02	0.02	0, 0.03	0.04	0, 0.07	0.02	0, 0.03	0.05	0.02, 0.07	0.09

Supplementary table 15. Relative contribution of GHGE by food group among households with a completed college education, 1989, 2020 ENIGHS.

		2008		2010		2012		2014		2016		2018		2020		Difference 2020-1989	
Food group	95% CI	Mean	95% CI	Mean	95% CI	Mean	95% CI	Mean	95% CI	Mean	95% CI	Mean	95% CI	Mean	95% CI		95% CI
Beef	22.13, 24.61	24.11	23.01, 25.19	23.56	22.53, 24.57	23.04	21.12, 24.94	21.13	19.88, 22.37	19.78	19.08, 20.46	20.70	20.04, 21.35	20.33	19.72, 20.92	-7.23	-8.1, -6.36
Dairy	16.86, 18.89	17.50	16.66, 18.33	16.93	16.1, 17.74	16.95	15.07, 18.82	15.52	14.6, 16.44	14.95	14.38, 15.51	13.96	13.47, 14.43	11.80	11.38, 12.2	-12.83	-13.66, -12.0
Corn	1.8, 2.4	2.23	1.98, 2.46	2.43	2.13, 2.71	2.12	1.82, 2.41	2.08	1.86, 2.29	2.24	2.06, 2.41	2.46	2.3, 2.62	2.41	2.27, 2.55	-0.24	-0.56, 0.08
Legume	0.99, 1.33	1.35	1.19, 1.5	1.21	1.05, 1.37	1.22	0.97, 1.46	1.33	1.13, 1.51	1.36	1.24, 1.47	1.29	1.2, 1.38	1.29	1.2, 1.37	-0.43	-0.68, -0.18
Beverages	9.97, 11.7	10.71	10.08, 11.33	11.11	10.31, 11.9	11.66	10.32, 13	11.62	10.69, 12.53	11.16	10.65, 11.65	10.42	9.95, 10.88	10.76	10.35, 11.16	4.9	4.42, 5.38
Sweet or salty	5.79, 7.53	6.47	5.7, 7.23	6.91	6.14, 7.67	7.78	6.23, 9.32	7.12	5.84, 8.38	7.17	6.6, 7.72	6.53	5.99, 7.06	5.74	5.29, 6.18	-2.33	-2.86, -1.8
Chicken	7.63, 8.95	9.24	8.64, 9.83	8.22	7.71, 8.73	7.31	6.19, 8.43	8.24	7.52, 8.94	8.77	8.37, 9.16	9.08	8.7, 9.46	9.08	8.72, 9.44	2.78	2.29, 3.27
Oil	1.86, 2.35	2.28	2.07, 2.48	2.30	2.07, 2.51	1.93	1.57, 2.29	2.39	2.15, 2.63	2.08	1.9, 2.24	2.28	2.11, 2.44	2.12	2, 2.24	-1.05	-1.39, -0.71
Vegetables	3.15, 3.68	3.57	3.36, 3.77	3.81	3.58, 4.03	3.98	3.44, 4.5	3.90	3.6, 4.19	3.83	3.65, 4.01	3.94	3.76, 4.11	4.39	4.19, 4.58	0.44	0.05, 0.83
Pork	1.84, 2.52	2.44	2.14, 2.73	2.42	2.14, 2.68	2.17	1.65, 2.69	2.37	2.03, 2.7	2.59	2.35, 2.81	2.76	2.51, 3	3.05	2.8, 3.28	0.14	-0.19, 0.47
Grain	1.94, 2.25	2.22	2.07, 2.35	2.35	2.07, 2.62	2.24	1.97, 2.49	2.15	1.97, 2.32	2.21	2.1, 2.31	2.23	2.09, 2.36	2.03	1.93, 2.12	-0.25	-0.54, 0.04
Seafood	3.02, 3.99	3.14	2.81, 3.46	4.08	3.56, 4.59	4.02	3.19, 4.84	3.01	2.66, 3.34	3.39	3.12, 3.65	3.16	2.91, 3.41	3.23	3.02, 3.44	1.0	0.7, 1.3
Egg	1.42, 1.92	1.72	1.57, 1.86	1.88	1.64, 2.11	1.43	1.21, 1.65	1.71	1.54, 1.87	1.92	1.79, 2.04	2.10	1.97, 2.22	2.09	1.96, 2.2	-0.08	-0.37, 0.21
Others	1.51, 2.23	1.81	1.48, 2.12	1.78	1.49, 2.05	2.04	1.49, 2.57	1.88	1.59, 2.16	1.85	1.65, 2.03	1.95	1.75, 2.13	2.05	1.83, 2.25	0.48	0.23, 0.73
Fruit	3.18, 3.99	3.38	3.14, 3.61	3.17	2.89, 3.44	2.74	2.34, 3.13	3.21	2.87, 3.53	3.29	3.09, 3.47	3.18	2.96, 3.38	3.01	2.84, 3.18	-0.43	-0.79, -0.07
Fastfood	8.05, 10.19	7.82	6.61, 9.02	7.83	7.04, 8.6	9.32	7.58, 11.05	12.30	11.18, 13.41	13.37	12.63, 14.1	13.88	13.19, 14.57	16.57	15.86, 17.27	15.11	14.78, 15.44
Seed	, 0.01, 0.2	0.03	0.01, 0.03	0.03	0.01, 0.04	0.05	0, 0.09	0.05	0.03, 0.07	0.07	0.04, 0.08	0.06	0.04, 0.08	0.06	0.03, 0.08	0.05	0.03, 0.07

Supplementary table 16. Relative contribution of GHGE by food group among households living in rural areas, 1992, 2020 ENIGHS.

	1992		1994		1996		1998		2000		2002		2004		2006	
Food group	Mean	95% CI	Mean	95% CI	Mean	95% CI	Mean	95% CI	Mean	95% CI	Mean	95% CI	Mean	95% CI	Mean	95% CI
Beef	8.79	7.34, 10.22	9.49	8.46, 10.52	10.49	9.31, 11.66	11.49	9.99, 12.98	12.58	11.1, 14.05	15.23	13.76, 16.69	16.61	15.32, 17.9	16.69	15.2, 18.17
Dairy	12.11	10.69, 13.51	11.56	10.41, 12.71	11.22	10.09, 12.33	11.47	10.19, 12.74	10.87	9.86, 11.88	11.80	10.66, 12.93	12.13	11.31, 12.95	12.54	11.76, 13.31
Corn	17.29	14.95, 19.62	15.33	13.91, 16.73	18.47	16.61, 20.32	16.90	15.21, 18.57	15.53	13.97, 17.07	13.38	11.97, 14.77	10.89	9.68, 12.09	9.38	8.54, 10.21
Legume	11.67	10.37, 12.95	10.40	9.39, 11.4	11.63	10.54, 12.72	10.40	9.47, 11.33	9.89	8.7, 11.06	8.76	7.67, 9.84	7.36	6.53, 8.18	6.28	5.71, 6.84
Beverages	9.29	7.71, 10.85	9.93	8.89, 10.97	7.47	6.45, 8.47	8.27	7.3, 9.23	7.93	7.02, 8.82	8.39	7.27, 9.51	8.86	7.82, 9.88	9.09	8.14, 10.02
Sweet or salty	4.39	3.24, 5.54	4.76	4.11, 5.4	3.75	3.22, 4.27	4.76	4.15, 5.36	5.03	4.22, 5.82	5.19	4.28, 6.09	5.36	4.59, 6.11	6.03	5.05, 6.99
Chicken	5.70	5.04, 6.35	6.35	5.67, 7.02	6.05	5.31, 6.79	5.19	4.54, 5.84	6.82	6.05, 7.58	7.48	6.69, 8.27	9.06	8.13, 9.98	9.52	8.76, 10.28
Oil	8.22	7.34, 9.1	8.33	7.45, 9.21	7.76	7.07, 8.45	7.50	6.74, 8.25	6.89	6.21, 7.56	6.75	6.1, 7.38	5.54	5.12, 5.95	4.51	4.18, 4.83
Vegetables	4.99	4.31, 5.65	5.09	4.56, 5.61	5.01	4.43, 5.58	5.50	4.87, 6.13	4.73	4.27, 5.19	4.89	4.55, 5.22	4.65	4.26, 5.02	4.90	4.28, 5.51
Pork	2.85	2.23, 3.46	3.21	2.64, 3.77	3.20	2.73, 3.66	3.56	3, 4.11	2.89	2.39, 3.38	2.79	1.99, 3.59	2.43	1.99, 2.86	2.58	2.21, 2.93
Grain	4.40	3.9, 4.88	3.82	3.43, 4.21	3.63	3.23, 4.03	3.80	3.35, 4.24	3.64	2.96, 4.31	3.15	2.79, 3.49	4.05	3.66, 4.42	4.21	3.83, 4.59
Seafood	2.18	1.34, 3.01	2.79	1.97, 3.6	2.63	2.16, 3.1	2.38	1.71, 3.04	3.69	2.03, 5.34	3.42	2.58, 4.24	2.36	1.76, 2.94	3.03	2.25, 3.8
Egg	2.89	2.43, 3.34	3.47	2.39, 4.53	2.79	2.47, 3.1	3.15	2.69, 3.6	3.17	2.75, 3.59	2.88	2.59, 3.15	3.27	2.89, 3.63	2.89	2.46, 3.32
Others	3.53	2.8, 4.25	3.15	2.77, 3.52	3.37	2.95, 3.78	3.21	2.66, 3.75	2.48	2.15, 2.8	2.45	2.1, 2.79	2.25	1.94, 2.55	2.08	1.85, 2.3
Fruit	1.27	0.98, 1.56	1.68	1.42, 1.92	1.37	1.14, 1.6	1.37	1.13, 1.6	1.93	1.6, 2.26	1.59	1.37, 1.79	1.70	1.44, 1.95	2.07	1.6, 2.54
Fastfood	0.38	0.21, 0.54	0.59	0.4, 0.77	1.07	0.76, 1.37	0.98	0.66, 1.28	1.91	1.48, 2.33	1.83	1.11, 2.55	3.48	2.79, 4.16	4.16	3.35, 4.95
Seed	0.06	0.02, 0.09	0.05	0.03, 0.07	0.09	0.03, 0.13	0.07	0.02, 0.1	0.03	0, 0.05	0.03	0.01, 0.03	0.02	0.01, 0.02	0.04	0.02, 0.06

Supplementary table 16. Relative contribution of GHGE by food group among households living in rural areas, 1992, 2020 ENIGHS.

	2008		2010		2012		2014		2016		2018		2020		Difference 2020-1992	
Food group	Mean	95% CI	Mean	95% CI	Mean	95% CI	Mean	95% CI	Mean	95% CI	Mean	95% CI	Mean	95% CI		95% CI
Beef	15.92	14.9, 16.92	15.58	14.58, 16.56	12.26	10.91, 13.6	11.71	10.91, 12.5	11.33	10.95, 11.69	11.70	11.32, 12.07	12.25	11.91, 12.58	3.46	2.89, 4.03
Dairy	12.34	11.57, 13.09	11.72	10.98, 12.44	12.12	11.32, 12.91	11.89	11.1, 12.66	12.24	11.89, 12.59	11.56	11.25, 11.86	10.55	10.29, 10.79	-1.56	-2.2, -0.92
Corn	10.83	9.86, 11.79	10.48	9.64, 11.31	12.49	11.14, 13.84	12.28	11.42, 13.14	11.19	10.67, 11.69	10.89	10.42, 11.36	10.70	10.25, 11.13	-6.59	-7.32, -5.86
Legume	7.74	7.2, 8.28	7.38	6.75, 8.01	6.82	6.12, 7.51	7.42	6.83, 8	6.46	6.14, 6.77	6.18	5.9, 6.45	5.49	5.23, 5.75	-6.18	-6.8, -5.56
Beverages	8.54	7.77, 9.31	8.90	8.13, 9.65	9.28	8.39, 10.17	8.70	8.1, 9.29	8.96	8.62, 9.3	8.56	8.28, 8.83	9.66	9.36, 9.96	0.37	-0.21, 0.95
Sweet or salty	4.81	4.29, 5.31	5.30	4.76, 5.84	6.85	6.08, 7.61	6.52	5.87, 7.16	6.31	6.02, 6.59	5.96	5.68, 6.23	5.21	4.99, 5.42	0.82	0.41, 1.23
Chicken	9.91	9.23, 10.58	9.38	8.55, 10.21	7.43	6.77, 8.08	8.24	7.63, 8.83	9.61	9.27, 9.93	9.59	9.31, 9.86	9.30	9.04, 9.54	3.6	3.13, 4.07
Oil	5.22	4.86, 5.56	5.16	4.68, 5.63	4.23	3.85, 4.61	4.55	4.13, 4.95	3.93	3.79, 4.07	3.99	3.86, 4.11	3.68	3.58, 3.78	-4.54	-5.07, -4.01
Vegetables	4.82	4.54, 5.08	5.31	4.91, 5.7	4.77	4.37, 5.15	4.96	4.61, 5.31	4.99	4.83, 5.14	4.77	4.65, 4.88	4.63	4.51, 4.73	-0.36	-0.79, 0.07
Pork	2.49	2.07, 2.9	2.47	2.16, 2.77	2.40	1.98, 2.81	2.17	1.81, 2.52	2.96	2.8, 3.11	3.20	3.04, 3.35	3.61	3.44, 3.76	0.76	0.43, 1.09
Grain	4.19	3.86, 4.51	4.21	3.82, 4.58	4.05	3.66, 4.43	3.91	3.64, 4.17	3.63	3.49, 3.75	3.44	3.31, 3.55	3.00	2.9, 3.09	-1.4	-1.8, -1
Seafood	3.27	2.76, 3.78	2.84	2.4, 3.27	3.40	2.5, 4.3	2.82	2.29, 3.35	3.15	2.74, 3.55	3.04	2.75, 3.33	2.91	2.65, 3.16	0.73	0.44, 1.02
Egg	3.38	3.13, 3.62	3.83	3.53, 4.11	2.79	2.55, 3.03	2.89	2.7, 3.07	3.27	3.14, 3.4	3.20	3.09, 3.29	3.12	3.02, 3.21	0.23	-0.1, 0.56
Others	1.97	1.71, 2.21	1.93	1.67, 2.19	2.23	1.88, 2.58	1.58	1.38, 1.76	1.63	1.54, 1.7	1.57	1.48, 1.64	1.68	1.59, 1.76	-1.85	-2.2, -1.5
Fruit	1.73	1.57, 1.87	1.64	1.41, 1.87	1.87	1.61, 2.12	1.72	1.54, 1.89	1.87	1.76, 1.96	1.69	1.61, 1.76	1.68	1.61, 1.75	0.41	0.18, 0.64
Fastfood	2.83	2.46, 3.18	3.83	3.32, 4.33	6.95	6.14, 7.74	8.61	7.69, 9.51	8.42	7.97, 8.87	10.60	10.12, 11.08	12.49	11.99, 12.99	12.11	11.86, 12.36
Seed	0.03	0.01, 0.04	0.03	0.01, 0.04	0.05	0.02, 0.06	0.04	0.02, 0.05	0.06	0.04, 0.06	0.06	0.04, 0.06	0.05	0.04, 0.05	-0.01	-0.06, 0.04

Supplementary table 17. Relative contribution of GHGE by food group among households living in small areas, 1992, 2020 ENIGHS.

	1992		1994		1996		1998		2000		2002		2004		2006	
Food group	Mean	95% CI	Mean	95% CI	Mean	95% CI	Mean	95% CI	Mean	95% CI	Mean	95% CI	Mean	95% CI	Mean	95% CI
Beef	16.72	14.22, 19.21	17.70	14.66, 20.73	17.57	15.32, 19.82	17.83	15.19, 20.47	21.54	19.09, 23.99	19.73	17.06, 22.39	19.76	16.75, 22.76	17.23	14.84, 19.61
Dairy	17.40	15.27, 19.52	17.20	14.3, 20.1	15.03	13.11, 16.94	16.19	14.14, 18.22	17.07	14.63, 19.5	15.45	13.45, 17.44	14.13	11.58, 16.68	12.50	11.04, 13.96
Corn	10.90	8.81, 12.98	9.25	7.81, 10.67	11.60	9.94, 13.25	9.44	7.04, 11.83	7.50	6.18, 8.81	8.82	6.45, 11.18	7.85	6.39, 9.29	7.71	6.61, 8.79
Legume	6.74	5.47, 8	7.81	6.1, 9.51	8.01	7.1, 8.92	6.65	5.66, 7.64	5.49	4.64, 6.33	5.76	5.05, 6.46	6.73	5.31, 8.14	5.95	5.16, 6.73
Beverages	6.89	5.43, 8.33	7.34	6.04, 8.64	6.15	5.39, 6.9	7.21	5.76, 8.65	6.01	4.86, 7.15	6.52	5.64, 7.4	7.84	6.83, 8.84	9.04	7.34, 10.74
Sweet or salty	5.30	3.77, 6.82	4.43	3.02, 5.83	4.88	3.9, 5.85	6.43	4.94, 7.91	4.44	3.45, 5.42	4.66	3.79, 5.51	6.34	4.56, 8.11	6.18	4.25, 8.1
Chicken	8.18	6.55, 9.8	8.39	6.92, 9.86	8.21	7.34, 9.07	7.85	6.85, 8.85	9.42	8.19, 10.64	9.69	8.25, 11.12	10.14	8.17, 12.1	11.12	9.29, 12.95
Oil	5.72	4.77, 6.65	5.25	4.14, 6.36	5.54	4.85, 6.21	4.67	4.03, 5.3	4.02	3.49, 4.53	4.46	3.92, 4.99	3.54	2.82, 4.26	4.01	3.32, 4.69
Vegetables	3.90	3.23, 4.57	4.09	3.62, 4.55	4.75	4.28, 5.21	4.51	3.88, 5.13	4.52	3.7, 5.33	4.99	4.38, 5.58	5.26	4.54, 5.97	4.55	3.69, 5.4
Pork	4.45	3.45, 5.44	3.81	3.17, 4.44	4.57	3.56, 5.57	4.69	3.74, 5.63	3.82	2.88, 4.76	3.74	3.14, 4.32	3.03	2.18, 3.87	3.36	2.53, 4.18
Grain	2.68	2.32, 3.03	2.60	2.19, 3	2.62	2.28, 2.94	2.49	2.14, 2.83	2.20	1.9, 2.5	2.69	2.17, 3.2	2.79	2.43, 3.15	2.73	2.39, 3.06
Seafood	2.60	1.33, 3.85	2.82	1.21, 4.43	2.63	2.05, 3.2	2.41	1.35, 3.46	3.63	1.93, 5.32	3.14	2.44, 3.82	2.03	1.05, 3.01	3.62	1.81, 5.42
Egg	2.85	2.34, 3.36	2.24	1.91, 2.56	2.73	2.34, 3.1	2.74	2.39, 3.07	2.62	2.3, 2.92	2.69	2.15, 3.23	3.31	2.6, 4	2.95	2.54, 3.35
Others	2.14	1.59, 2.67	2.71	2.14, 3.26	2.26	1.79, 2.72	2.33	1.62, 3.03	1.87	1.37, 2.37	1.58	1.21, 1.94	1.68	1.34, 2.01	1.90	1.3, 2.48
Fruit	1.86	1.31, 2.4	2.05	1.39, 2.69	1.51	1.22, 1.78	1.49	1.24, 1.73	2.47	1.98, 2.95	2.11	1.41, 2.8	1.93	1.49, 2.35	1.53	1.24, 1.81
Fastfood	1.61	0.85, 2.35	2.27	0.8, 3.73	1.95	1.31, 2.57	3.05	1.25, 4.84	3.36	1.49, 5.23	3.96	2.25, 5.65	3.60	2.5, 4.69	5.57	3.86, 7.27
Seed	0.07	, 0.03, 0.18	0.02	0, 0.03	0.02	0, 0.02	0.02	0, 0.04	0.01	0, 0	0.01	0, 0.02	0.04	0, 0.07	0.04	0.01, 0.05

Supplementary table 17. Relative contribution of GHGE by food group among households living in small areas, 1992, 2020 ENIGHS.

	2008		2010		2012		2014		2016		2018		2020		Difference 2020-1992	
Food group	Mean	95% CI	Mean	95% CI	Mean	95% CI	Mean	95% CI	Mean	95% CI	Mean	95% CI	Mean	95% CI		95% CI
Beef	19.51	18.23, 20.78	20.19	18.63, 21.73	17.57	16.04, 19.08	15.83	14.66, 16.99	14.47	13.91, 15.03	15.16	14.47, 15.85	15.64	15.12, 16.14	-1.08	-1.82, -0.34
Dairy	14.25	13.23, 15.26	13.72	12.8, 14.64	12.77	11.58, 13.95	13.78	12.77, 14.79	13.65	13.09, 14.21	11.76	11.28, 12.23	10.98	10.55, 11.4	-6.42	-7.16, -5.68
Corn	6.94	5.91, 7.95	6.93	6.11, 7.73	6.09	4.67, 7.5	7.00	6.08, 7.91	6.23	5.89, 6.56	5.66	5.33, 5.98	6.26	5.9, 6.61	-4.64	-5.24, -4.04
Legume	5.00	4.53, 5.46	5.16	4.64, 5.67	4.48	3.8, 5.14	4.97	4.49, 5.44	4.44	4.15, 4.72	3.96	3.69, 4.22	3.94	3.63, 4.24	-2.8	-3.28, -2.32
Beverages	8.33	7.55, 9.11	9.03	7.9, 10.15	8.56	7.56, 9.54	9.48	8.43, 10.52	9.42	8.99, 9.85	8.43	8.03, 8.82	9.00	8.63, 9.35	2.11	1.6, 2.62
Sweet or salty	5.47	4.65, 6.28	4.92	4.38, 5.46	6.64	5.54, 7.72	6.09	5.33, 6.84	6.15	5.74, 6.56	5.81	5.39, 6.21	4.89	4.56, 5.21	-0.41	-0.85, 0.03
Chicken	10.86	9.87, 11.85	9.86	9.06, 10.65	9.55	8.35, 10.75	9.38	8.79, 9.96	10.65	10.18, 11.11	11.26	10.86, 11.65	10.76	10.34, 11.18	2.58	2.03, 3.13
Oil	4.11	3.58, 4.62	3.99	3.68, 4.3	3.28	2.79, 3.75	3.11	2.83, 3.37	3.20	3.02, 3.36	3.12	2.96, 3.27	3.18	3, 3.34	-2.54	-2.99, -2.09
Vegetables	5.03	4.62, 5.43	5.06	4.71, 5.41	4.97	4.21, 5.72	5.17	4.7, 5.64	5.12	4.83, 5.39	4.85	4.67, 5.02	4.93	4.7, 5.14	1.03	0.64, 1.42
Pork	3.38	2.72, 4.04	3.33	2.85, 3.8	3.68	2.99, 4.37	2.91	2.51, 3.3	3.58	3.28, 3.87	4.14	3.81, 4.46	4.50	4.23, 4.75	0.05	-0.36, 0.46
Grain	2.93	2.68, 3.17	3.18	2.83, 3.52	3.19	2.71, 3.66	3.01	2.71, 3.29	3.03	2.9, 3.16	2.72	2.61, 2.81	2.55	2.41, 2.68	-0.13	-0.45, 0.19
Seafood	2.91	2.14, 3.68	2.62	2.13, 3.1	2.71	1.92, 3.5	2.67	2.04, 3.28	2.66	2.37, 2.93	2.63	2.29, 2.95	2.67	2.34, 2.98	0.07	-0.25, 0.39
Egg	2.92	2.64, 3.18	3.00	2.73, 3.26	2.23	1.94, 2.5	2.49	2.27, 2.69	2.76	2.62, 2.89	2.80	2.62, 2.96	2.87	2.75, 2.98	0.02	-0.31, 0.35
Others	1.32	1.09, 1.54	1.61	1.32, 1.88	1.62	1.21, 2.03	1.37	1.15, 1.57	1.58	1.4, 1.76	1.51	1.36, 1.65	1.73	1.55, 1.9	-0.41	-0.69, -0.13
Fruit	1.71	1.52, 1.9	1.69	1.47, 1.89	1.88	1.41, 2.35	2.01	1.72, 2.3	1.90	1.77, 2.02	1.73	1.63, 1.83	1.72	1.59, 1.85	-0.14	-0.41, 0.13
Fastfood	5.28	4.15, 6.41	5.66	4.87, 6.45	10.65	8.9, 12.4	10.68	9.13, 12.22	11.10	10.44, 11.76	14.43	13.54, 15.31	14.36	13.62, 15.09	12.75	12.42, 13.08
Seed	0.03	0, 0.05	0.05	0.02, 0.06	0.13	, 0.06, 0.32	0.05	0.01, 0.09	0.04	0.03, 0.05	0.05	0.03, 0.05	0.04	0.02, 0.04	-0.03	-0.08, 0.02

Supplementary table 18. Relative contribution of GHGE by food group among households living in medium areas, 1992, 2020 ENIGHS.

	1992		1994		1996		1998		2000		2002		2004		2006	
Food group	Mean	95% CI	Mean	95% CI	Mean	95% CI	Mean	95% CI	Mean	95% CI	Mean	95% CI	Mean	95% CI	Mean	95% CI
Beef	20.21	17.28, 23.14	22.61	20.6, 24.61	20.29	18.66, 21.91	24.22	21.99, 26.43	23.22	21.56, 24.87	25.47	23.83, 27.09	21.81	20.81, 22.81	22.40	21.21, 23.59
Dairy	19.43	17.84, 21.01	18.56	16.32, 20.79	17.72	16.08, 19.34	16.84	14.69, 18.98	16.62	15.19, 18.04	15.81	14.64, 16.97	15.92	14.93, 16.91	15.24	14.16, 16.31
Corn	7.55	5.44, 9.64	6.44	5.18, 7.69	7.34	6.13, 8.53	5.98	5.18, 6.78	5.45	4.52, 6.36	5.42	4.75, 6.07	5.60	5.02, 6.16	4.44	4.09, 4.77
Legume	7.14	5.58, 8.69	4.93	4.07, 5.77	6.67	6.03, 7.3	5.57	4.41, 6.72	4.43	3.88, 4.97	4.28	3.82, 4.72	4.51	4.07, 4.95	3.32	2.97, 3.66
Beverages	5.36	4.26, 6.45	6.85	5.52, 8.17	6.26	5.51, 7.01	8.10	6.83, 9.35	7.76	6.14, 9.37	8.42	7.72, 9.1	9.45	8.78, 10.11	9.70	8.97, 10.43
Sweet or salty	4.94	3.48, 6.39	5.75	4.31, 7.19	5.11	3.93, 6.28	4.86	4.03, 5.68	5.48	4.35, 6.61	4.83	4.07, 5.58	5.29	4.68, 5.88	4.75	4.17, 5.33
Chicken	7.08	6.18, 7.96	8.92	8.04, 9.79	8.24	6.89, 9.58	8.11	6.37, 9.85	8.43	7.61, 9.24	9.34	8.3, 10.36	9.75	9.07, 10.43	10.61	9.82, 11.4
Oil	4.93	4.1, 5.75	4.70	4.19, 5.19	4.56	4.01, 5.1	3.66	3.1, 4.21	3.77	3.35, 4.18	3.47	3.1, 3.84	3.29	2.98, 3.59	2.95	2.75, 3.14
Vegetables	4.34	3.7, 4.96	4.30	3.65, 4.94	4.85	4.01, 5.67	4.11	3.64, 4.58	3.96	3.37, 4.54	4.32	3.89, 4.75	3.84	3.39, 4.29	3.86	3.47, 4.25
Pork	4.79	3.89, 5.69	3.78	3.08, 4.48	4.35	3.88, 4.81	4.31	3.65, 4.97	5.14	3.7, 6.58	3.10	2.65, 3.54	3.20	2.71, 3.68	3.08	2.71, 3.44
Grain	2.57	2.11, 3.02	2.24	2.02, 2.45	2.43	2.22, 2.63	2.37	2.01, 2.71	1.96	1.77, 2.14	2.15	2.01, 2.28	2.46	2.28, 2.64	2.42	2.28, 2.55
Seafood	3.14	2.21, 4.05	2.15	1.51, 2.77	2.56	1.72, 3.38	2.25	1.62, 2.87	2.91	2.26, 3.55	2.79	2.32, 3.25	2.27	1.82, 2.72	2.76	2.35, 3.16
Egg	2.86	2.46, 3.25	2.45	2.2, 2.7	2.68	2.49, 2.87	2.71	2.36, 3.05	2.26	2.06, 2.46	2.30	2.07, 2.52	2.53	2.28, 2.78	2.46	2.25, 2.67
Others	1.53	1.17, 1.88	1.97	1.44, 2.48	1.91	1.58, 2.23	2.35	1.76, 2.94	2.12	1.69, 2.53	1.61	1.32, 1.89	1.61	1.37, 1.85	1.61	1.38, 1.84
Fruit	1.99	1.6, 2.38	2.78	2.14, 3.41	1.99	1.71, 2.26	1.68	1.38, 1.97	2.17	1.85, 2.47	1.84	1.5, 2.17	1.51	1.35, 1.65	2.04	1.83, 2.24
Fastfood	2.14	0.97, 3.3	1.54	0.86, 2.21	3.02	2.33, 3.69	2.86	1.95, 3.75	4.31	3.41, 5.2	4.84	3.72, 5.95	6.93	5.87, 7.98	8.30	7.22, 9.38
Seed	0.01	0, 0.02	0.02	0.01, 0.03	0.02	0.01, 0.02	0.01	0, 0.01	0.01	0, 0.01	0.02	0, 0.02	0.02	0, 0.02	0.03	0, 0.06

Supplementary table 18. Relative contribution of GHGE by food group among households living in medium areas, 1992, 2020 ENIGHS.

	2008		2010		2012		2014		2016		2018		2020		Difference 2020-1992	
Food group	Mean	95% CI	Mean	95% CI	Mean	95% CI	Mean	95% CI	Mean	95% CI	Mean	95% CI	Mean	95% CI		95% CI
Beef	22.15	21.08, 23.22	22.80	21.94, 23.64	19.38	18.07, 20.67	18.13	17.01, 19.23	17.37	16.77, 17.97	17.48	16.84, 18.12	18.33	17.75, 18.9	-1.88	-2.67, -1.09
Dairy	14.64	13.88, 15.38	14.36	13.65, 15.07	13.76	12.53, 14.98	14.09	13.27, 14.9	13.17	12.69, 13.63	12.50	12.11, 12.87	11.12	10.73, 11.5	-8.31	-9.08, -7.54
Corn	4.77	4.48, 5.05	5.07	4.71, 5.42	4.95	4.38, 5.52	4.82	4.32, 5.31	4.54	4.31, 4.76	4.61	4.35, 4.86	4.36	4.17, 4.55	-3.19	-3.7, -2.68
Legume	3.84	3.53, 4.14	3.79	3.44, 4.13	3.48	2.95, 4	3.72	3.31, 4.12	3.36	3.11, 3.6	3.14	2.97, 3.31	2.90	2.72, 3.07	-4.24	-4.73, -3.75
Beverages	9.93	9.32, 10.53	9.23	8.66, 9.8	9.59	8.58, 10.59	9.47	8.8, 10.14	10.17	9.73, 10.59	9.91	9.53, 10.28	10.15	9.78, 10.51	4.79	4.33, 5.25
Sweet or salty	5.27	4.68, 5.85	5.23	4.74, 5.72	6.55	5.37, 7.72	6.42	5.69, 7.13	5.85	5.45, 6.24	5.41	4.97, 5.85	4.88	4.53, 5.22	-0.06	-0.49, 0.37
Chicken	10.54	9.96, 11.12	9.90	9.32, 10.46	9.78	8.78, 10.77	9.39	8.66, 10.11	10.14	9.74, 10.53	10.78	10.35, 11.2	10.18	9.85, 10.5	3.1	2.58, 3.62
Oil	3.15	2.88, 3.41	3.06	2.82, 3.3	2.71	2.29, 3.12	3.07	2.79, 3.33	2.99	2.82, 3.16	2.91	2.74, 3.06	2.82	2.67, 2.96	-2.11	-2.53, -1.69
Vegetables	4.17	3.96, 4.38	4.39	4, 4.76	4.09	3.61, 4.55	4.67	4.25, 5.07	4.35	4.17, 4.53	4.39	4.22, 4.55	4.37	4.19, 4.54	0.03	-0.37, 0.43
Pork	3.25	2.92, 3.57	3.50	3.04, 3.94	2.83	2.25, 3.39	3.19	2.73, 3.65	3.85	3.57, 4.12	4.03	3.72, 4.32	4.38	4.11, 4.63	-0.41	-0.83, 0.01
Grain	2.83	2.64, 3	2.73	2.54, 2.9	2.66	2.19, 3.11	2.65	2.42, 2.87	2.71	2.53, 2.89	2.45	2.33, 2.56	2.37	2.26, 2.47	-0.2	-0.51, 0.11
Seafood	2.56	2.22, 2.89	2.56	2.19, 2.92	2.38	1.64, 3.1	2.27	1.94, 2.59	2.52	2.28, 2.75	2.23	2.03, 2.42	2.25	2.05, 2.43	-0.89	-1.23, -0.55
Egg	2.95	2.68, 3.21	2.80	2.59, 3	2.22	1.97, 2.45	2.49	2.29, 2.68	2.57	2.45, 2.67	2.82	2.68, 2.94	2.65	2.51, 2.78	-0.21	-0.54, 0.12
Others	1.95	1.62, 2.28	1.49	1.29, 1.68	2.08	1.6, 2.54	1.72	1.37, 2.06	1.70	1.54, 1.85	1.61	1.46, 1.75	1.73	1.58, 1.87	0.2	-0.04, 0.44
Fruit	1.78	1.62, 1.92	1.81	1.61, 2	1.82	1.49, 2.14	1.97	1.73, 2.2	2.07	1.92, 2.21	1.97	1.8, 2.13	1.98	1.87, 2.08	-0.01	-0.29, 0.27
Fastfood	6.19	5.48, 6.89	7.25	6.45, 8.05	11.72	10, 13.43	11.91	10.38, 13.44	12.59	11.81, 13.36	13.71	12.91, 14.5	15.48	14.77, 16.18	13.34	12.98, 13.7
Seed	0.03	0.01, 0.04	0.02	0.01, 0.03	0.03	0, 0.06	0.03	0.01, 0.03	0.04	0.02, 0.04	0.05	0.03, 0.06	0.04	0.02, 0.05	0.03	0.01, 0.05

Supplementary table 19. Relative contribution of GHGE by food group among households living in metropolitan areas, 1992, 2020 ENIGHS.

	1992		1994		1996		1998		2000		2002		2004		2006	
Food group	Mean	95% CI	Mean	95% CI	Mean	95% CI	Mean	95% CI	Mean	95% CI	Mean	95% CI	Mean	95% CI	Mean	95% CI
Beef	26.17	25.17, 27.17	27.35	26.44, 28.24	25.68	24.94, 26.41	25.72	24.86, 26.56	26.80	25.75, 27.83	26.66	25.85, 27.47	25.55	25.02, 26.07	23.80	23.2, 24.38
Dairy	19.97	19.16, 20.76	19.51	18.5, 20.5	18.69	17.98, 19.4	19.49	18.81, 20.17	17.78	16.86, 18.69	17.89	17.23, 18.54	17.45	17.01, 17.89	15.69	15.25, 16.13
Corn	4.32	3.94, 4.68	3.72	3.41, 4.03	4.55	4.18, 4.92	4.08	3.85, 4.29	3.53	3.28, 3.77	3.73	3.44, 4.01	3.56	3.38, 3.73	3.32	3.16, 3.47
Legume	3.83	3.43, 4.21	3.38	3.04, 3.71	4.50	4.06, 4.92	3.52	3.31, 3.73	2.93	2.65, 3.2	2.83	2.59, 3.05	2.58	2.44, 2.71	2.25	2.11, 2.37
Beverages	6.86	6.37, 7.35	7.03	6.58, 7.46	6.63	6.19, 7.06	7.97	7.48, 8.45	7.73	7.22, 8.23	7.85	7.43, 8.26	8.87	8.59, 9.15	10.05	9.68, 10.41
Sweet or salty	5.22	4.69, 5.74	5.03	4.42, 5.62	4.42	3.97, 4.85	5.36	4.79, 5.92	5.26	4.66, 5.85	4.99	4.44, 5.53	6.12	5.77, 6.45	5.79	5.39, 6.18
Chicken	9.11	8.63, 9.58	9.33	8.46, 10.19	7.96	7.6, 8.31	8.30	7.91, 8.67	8.29	7.82, 8.76	10.03	9.58, 10.48	9.81	9.52, 10.08	10.04	9.69, 10.37
Oil	3.42	3.15, 3.69	3.58	3.32, 3.82	4.02	3.8, 4.24	3.51	3.32, 3.69	3.94	3.7, 4.18	2.80	2.61, 2.97	2.81	2.69, 2.93	2.51	2.38, 2.62
Vegetables	4.20	3.98, 4.41	4.04	3.75, 4.33	4.82	4.61, 5.02	4.18	4.02, 4.34	3.99	3.71, 4.26	4.04	3.85, 4.22	3.68	3.57, 3.79	3.68	3.55, 3.8
Pork	3.68	3.35, 4	3.76	3.41, 4.1	3.48	3.2, 3.76	3.83	3.54, 4.1	3.70	3.3, 4.1	2.90	2.63, 3.16	2.63	2.45, 2.8	2.77	2.58, 2.95
Grain	2.48	2.3, 2.64	2.09	1.98, 2.19	2.49	2.34, 2.63	2.19	2.09, 2.28	2.13	2, 2.25	2.13	2, 2.24	2.46	2.37, 2.53	2.44	2.35, 2.52
Seafood	1.99	1.76, 2.21	2.28	2.03, 2.53	2.01	1.77, 2.25	1.93	1.7, 2.15	2.02	1.71, 2.32	3.02	2.75, 3.27	1.68	1.51, 1.84	2.36	2.18, 2.52
Egg	2.40	2.25, 2.53	2.30	2.17, 2.43	2.67	2.51, 2.83	2.48	2.34, 2.62	2.28	2.1, 2.46	2.20	2.07, 2.32	2.48	2.38, 2.58	2.25	2.14, 2.35
Others	1.76	1.47, 2.04	1.78	1.56, 1.98	1.83	1.67, 1.99	2.00	1.79, 2.21	2.31	1.94, 2.67	1.99	1.78, 2.19	2.06	1.9, 2.21	1.89	1.7, 2.06
Fruit	2.51	2.33, 2.67	2.57	2.4, 2.74	2.44	2.25, 2.62	2.17	2.01, 2.33	2.64	2.41, 2.85	2.19	2.01, 2.37	2.27	2.15, 2.38	2.50	2.35, 2.63
Fastfood	2.08	1.71, 2.44	2.23	1.88, 2.57	3.78	3.26, 4.3	3.26	2.86, 3.65	4.65	4.12, 5.17	4.74	4.18, 5.29	5.97	5.56, 6.37	8.64	8.13, 9.15
Seed	0.01	0, 0.01	0.02	0.01, 0.03	0.01	0, 0.01	0.01	0, 0.01	0.02	0, 0.02	0.01	0, 0.01	0.02	0.01, 0.02	0.04	0.01, 0.06

Supplementary table 19. Relative contribution of GHGE by food group among households living in metropolitan areas, 1992, 2020 ENIGHS.

	2008		2010		2012		2014		2016		2018		2020		Difference 2020-1992	
Food group	Mean	95% CI	Mean	95% CI	Mean	95% CI	Mean	95% CI	Mean	95% CI	Mean	95% CI	Mean	95% CI		95% CI
Beef	24.38	23.88, 24.87	24.13	23.61, 24.63	23.17	22.19, 24.14	21.24	20.65, 21.83	19.60	19.25, 19.94	20.28	19.93, 20.63	20.39	20.04, 20.73	-5.78	-6.64, -4.92
Dairy	15.06	14.7, 15.4	15.24	14.85, 15.61	14.98	14.15, 15.81	14.82	14.27, 15.36	13.95	13.69, 14.21	13.62	13.34, 13.88	11.59	11.36, 11.81	-8.38	-9.15, -7.61
Corn	3.64	3.44, 3.83	3.49	3.35, 3.62	3.27	3.01, 3.51	3.23	3.09, 3.36	3.20	3.11, 3.28	3.26	3.16, 3.35	3.28	3.2, 3.36	-1.04	-1.44, -0.64
Legume	2.53	2.41, 2.65	2.45	2.33, 2.56	2.28	2.04, 2.5	2.47	2.33, 2.6	2.16	2.08, 2.22	2.15	2.07, 2.22	2.11	2.04, 2.17	-1.72	-2.09, -1.35
Beverages	10.45	10.15, 10.75	10.18	9.84, 10.51	10.33	9.71, 10.94	10.49	10.08, 10.88	10.65	10.4, 10.88	10.36	10.12, 10.6	10.56	10.34, 10.77	3.7	3.19, 4.21
Sweet or salty	5.39	5.05, 5.72	5.75	5.41, 6.09	6.82	6.04, 7.59	6.33	5.77, 6.87	6.11	5.84, 6.37	5.71	5.44, 5.97	4.94	4.72, 5.15	-0.28	-0.72, 0.16
Chicken	10.47	10.19, 10.74	9.39	9.12, 9.66	8.75	8.19, 9.31	9.36	9, 9.71	10.24	10.02, 10.46	10.07	9.83, 10.3	10.13	9.93, 10.32	1.02	0.45, 1.59
Oil	2.73	2.62, 2.82	2.84	2.71, 2.96	2.52	2.31, 2.72	2.81	2.68, 2.94	2.77	2.67, 2.85	2.77	2.67, 2.85	2.69	2.61, 2.77	-0.73	-1.09, -0.37
Vegetables	4.12	4, 4.23	4.14	4.02, 4.24	4.09	3.85, 4.32	4.36	4.18, 4.52	4.13	4.03, 4.22	4.04	3.94, 4.13	4.30	4.21, 4.39	0.1	-0.3, 0.5
Pork	2.62	2.46, 2.76	2.63	2.48, 2.76	2.79	2.49, 3.08	2.87	2.66, 3.08	3.17	3.03, 3.29	3.42	3.27, 3.56	3.69	3.56, 3.82	0.01	-0.36, 0.38
Grain	2.61	2.52, 2.68	2.62	2.51, 2.71	2.51	2.37, 2.63	2.62	2.53, 2.71	2.55	2.48, 2.6	2.53	2.45, 2.6	2.27	2.22, 2.32	-0.21	-0.52, 0.1
Seafood	2.37	2.23, 2.49	2.65	2.47, 2.82	2.37	2.08, 2.66	2.22	2.05, 2.38	2.43	2.31, 2.54	2.17	2.06, 2.27	2.33	2.23, 2.42	0.34	0.06, 0.62
Egg	2.62	2.51, 2.72	2.70	2.58, 2.81	2.02	1.86, 2.18	2.42	2.3, 2.52	2.52	2.45, 2.58	2.73	2.64, 2.8	2.75	2.67, 2.82	0.35	0.04, 0.66
Others	1.90	1.74, 2.05	2.02	1.8, 2.23	2.12	1.84, 2.39	1.90	1.74, 2.06	1.88	1.78, 1.97	2.09	1.98, 2.19	2.11	2.01, 2.21	0.35	0.09, 0.61
Fruit	2.40	2.3, 2.49	2.31	2.2, 2.42	2.52	2.28, 2.75	2.42	2.27, 2.56	2.44	2.35, 2.53	2.34	2.24, 2.43	2.34	2.25, 2.41	-0.17	-0.48, 0.14
Fastfood	6.70	6.31, 7.08	7.44	7.01, 7.86	9.43	8.58, 10.27	10.40	9.87, 10.92	12.16	11.77, 12.55	12.43	12.04, 12.81	14.46	14.06, 14.84	12.38	12.03, 12.73
Seed	0.03	0.01, 0.04	0.02	0.01, 0.02	0.03	0.01, 0.04	0.03	0.02, 0.03	0.04	0.03, 0.04	0.03	0.02, 0.03	0.04	0.02, 0.04	0.03	0.01, 0.05

References
1. Guzmán, Soria D, Taboada, González P, Aguilar, Virgen Q, Baltierra, Trejo E, Marquez, Benavides L. Environmental impact of corn tortilla production: a case study. Appl Sci. 2019;9(22):4852.

2. Your Ethical Guide To Buy Better [Internet]. Disponible en: https://www.healabel.com

3. Clune S, Crossin E, Verghese K. Systematic review of greenhouse gas emissions for different fresh food categories. Eco, Effic Agric Food Syst Sel Pap Addressing Glob Chall Food Syst Those Present Conf "LCA Feed Planet Energy Life" 6, 8 Oct 2015 Stresa Milan Expo Italy. 1 de enero de 2017;140:766, 83.

4. Vergel, Rangel GA, Escamilla, García PE, Camarillo, López RH, Esquivel, Guzmán JA, Pérez, Soto F. The environmental impact of nopal (Opuntia ficus, indica) production in Mexico City, Mexico through a life cycle assessment (LCA). Environ Dev Sustain. 2021;1, 28.
